# Ticks and Tick-Borne Microorganisms in Australian Wildlife: A Scoping One Health Evidence Synthesis of Reported Associations and Knowledge Gaps

**DOI:** 10.3390/pathogens15060646

**Published:** 2026-06-18

**Authors:** Kabir Brar, Bahar E. Mustafa, Ian Beveridge, Charles Gauci, Abdul Jabbar, Abdul Ghafar

**Affiliations:** Department of Veterinary Biosciences, Melbourne Veterinary School, University of Melbourne, Werribee, VIC 3030, Australia; kabirbrar99@gmail.com (K.B.); bahare.mustafa@student.unimelb.edu.au (B.E.M.); ibeve@unimelb.edu.au (I.B.); charlesg@unimelb.edu.au (C.G.)

**Keywords:** emerging infectious diseases, One Health, parasite–host interactions, ticks, tick-borne pathogens, wildlife

## Abstract

Ticks are haematophagous ectoparasites and vectors of a diverse range of pathogens, exerting substantial impacts on wildlife, domestic animals and public health. In Australia, despite the country’s rich and unique biodiversity, a comprehensive understanding of ticks and tick-borne pathogens associated with wildlife remains limited. Environmental change, urban expansion and climate variability are increasingly disrupting wildlife habitats, potentially intensifying interactions between wildlife hosts, ticks and humans. A broad evidence synthesis of studies published between January 1940 and March 2024 was conducted, retrieving 133 eligible records from Web of Science, CABI Abstracts and PubMed databases. Fifty tick species parasitising 160 wildlife species were identified, predominantly from the genera *Ixodes*, *Amblyomma* and *Haemaphysalis*. The most commonly reported hosts included marsupials, particularly bandicoots, wallabies and possums, with notable tick species being *Ixodes tasmani*, *Ixodes holocyclus* and *Amblyomma triguttatum*. Microorganism records were relatively limited and mostly represented molecular detections or reported associations, including *Babesia*, *Borrelia*, *Coxiella*, *Rickettsia* and *Theileria* species, rather than confirmed vector competence, reservoir status or pathogenicity. Key limitations included geographic sampling biases towards eastern Australia, limited molecular identification of ticks and infrequent pathogen screening, particularly regarding the ecology, epidemiology and molecular diversity of host–vector–microorganism interactions. Improved surveillance, expanded molecular characterisation, and integrated One Health investigations are required to better understand the ecological and public health significance of these host–vector–microorganism interactions.

## 1. Introduction

Ticks are obligate haematophagous parasites of vertebrates worldwide and are found throughout Australia [1,2,3,4,5,6,7,8]. Their effects on humans and domestic animals have been studied extensively and are relatively well-understood [1,2,3,4,5,6], reflecting both their direct pathology and their role as vectors. Blood-feeding is essential for tick survival, development, and reproduction, and underpins their ability to transmit microorganisms and cause direct pathogenic effects in hosts [7]. Ticks can cause direct harm to their hosts through anaemia in heavy infestations and through the secretion of (neuro)toxins resulting in paralysis (as in *Ixodes holocyclus*) and allergies such as alpha-gal syndrome [3,8]. However, the primary significance of ticks is due to their capacity to carry and transmit a diverse array of pathogens to animals and humans [9].

Ticks are among the most important arthropod vectors of human and animal pathogens globally [10,11], and many of the diseases they transmit, including Lyme disease, tick-borne encephalitis, and Rocky Mountain spotted fever, are maintained in wildlife reservoirs [12]. Australia supports a distinctive vertebrate fauna of over 8000 described species, including approximately (400) mammals, (850) birds, (917) reptiles, (~5000) fishes and (240) amphibians, an increasing number of which are affected by habitat modification, urban expansion and climate change [13,14,15,16,17,18]. Environmental change and human encroachment into wildlife habitats can alter host–vector–environment interactions, affect wildlife health and movement, and increase contact among wildlife, domestic animals and people, thereby creating opportunities for tick exposure and potential spillover [18,19,20,21,22]. The most recent emerging diseases in domestic animals in Australia are tick-borne ehrlichiosis (dogs) and theileriosis (cattle), but their eco-epidemiology is not yet fully understood [23].

Notably, there has been increasing public and clinical attention surrounding tick-associated illnesses in Australia, partly due to improved awareness and surveillance [24]. Some affected individuals report symptom complexes that overlap partially with those described for Lyme borreliosis; however, there is currently no evidence supporting the endemic presence of *Borrelia burgdorferi sensu lato*, the causative agent of Lyme disease in North America and Europe, in Australia [24,25]. Consequently, the term ‘debilitating symptom complex attributed to ticks’ (DSCATT) has been adopted in Australia to describe persistent tick-associated symptom presentations where a definitive infectious cause has not been established [24]. The aetiology of DSCATT remains unresolved and is likely multifactorial. These ongoing uncertainties highlight the importance of investigating the diversity and ecology of Australian ticks and their associated microorganisms within a One Health framework.

Despite Australia’s unique wildlife fauna and diverse climatic regions, current knowledge of wildlife-associated ticks and their microorganisms remains fragmented across host species, geographic regions, and pathogen groups and findings from other regions are not always transferable to the Australian context. This review therefore aims to synthesise current knowledge on ticks and tick-borne microorganisms associated with Australian wildlife, with emphasis on host associations, geographic distribution, diagnostic approaches, pathogen detection, and One Health implications. By collating available evidence and identifying methodological and ecological knowledge gaps, this review provides a foundation for future surveillance, molecular characterisation, and risk-based investigations of wildlife-associated ticks and their microorganisms in Australia.

## 2. Materials and Methods

### 2.1. Literature Search

This review was conducted as a broad scoping evidence synthesis guided by PRISMA (Preferred Reporting Items for Systematic Reviews and Meta-Analyses) search principles (File S1). Accordingly, a broad literature search was undertaken to identify studies investigating ticks and tick-borne pathogens (TBPs) associated with Australian wildlife using three databases: Web of Science, CABI Abstracts, and PubMed. The search strategy employed different combinations of key terms, including “tick(s)” or “tick-borne” combined with pathogen-related terms such as “pathogen(s),” “zoono*” (zoonotic, zoonoses, zoonosis), “commensal(s),” “parasitism,” “virus(es),” “arbovirus(es),” “flavivirus(es),” “reovirus(es),” “orbivirus(es),” “bacteria,” “microorganism(s),” “microbe(s),” *Rickettsia*, *Bartonella*, *Coxiella*, *Ehrlichia*, *Neoehrlichia*, *Borrelia*, and *Anaplasma*. Protozoan and parasitic terms were also incorporated, including “protozoa,” “piroplasm(s),” “apicomplexa,” *Babesia*, *Theileria*, *Hepatozoon*, and *Trypanosoma*. To ensure comprehensive coverage, wildlife-related terms such as “wildlife,” “wild animal(s),” “undomesticated,” and “reservoir host” were included, along with geographical terms “Australia,” “Australian,” “Oceania,” and “Australasia.” In addition to database searches, the bibliographies of relevant articles were screened to identify additional studies for assessment.

### 2.2. Literature Selection and Assessment

**Figure 1:**
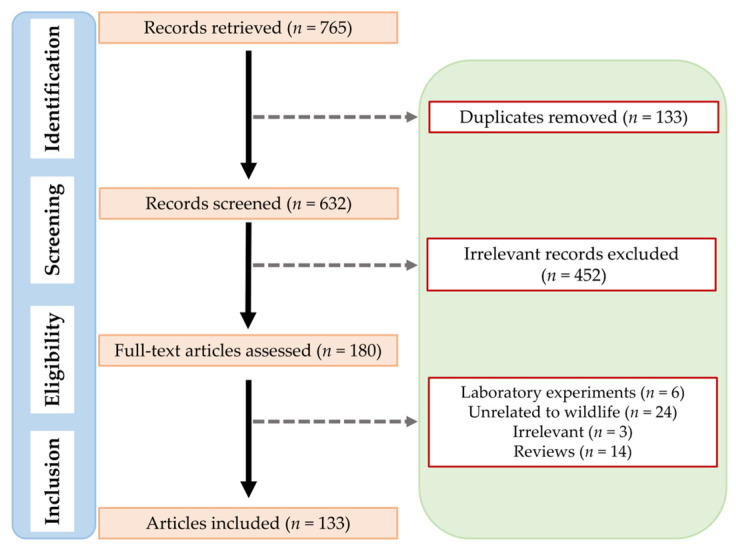
Process of literature identification, screening and inclusion for the review of tick and tick-borne microorganisms in wildlife in Australia.

The study selection process, guided by PRISMA principles, is illustrated in Figure 1. Searches were conducted in PubMed, CABI Abstracts, and Web of Science, covering English-language studies published from January 1940 to March 2024. This review was not registered, and no formal protocol was published; the methods followed established scoping-review guidance.

Eligible records included full-text peer-reviewed original research articles, postgraduate theses, conference proceedings and case reports, with original journal articles preferred where duplicate data were available. Titles and abstracts were first screened for relevance to ticks, tick-borne microorganisms and Australian wildlife, followed by full-text assessment against predefined eligibility criteria. Screening and study selection were performed by one reviewer and independently verified by two others, with any discrepancies resolved by discussion and consensus. Records were excluded if they were books or review articles, experimental or laboratory infection studies, studies of ticks not collected from wildlife or wildlife habitats, or studies in which ticks or wildlife samples were not of Australian origin. In total, 133 records were included 64 focused exclusively on ticks, 20 focused on microorganisms, and 49 contained data relevant to both and were therefore included in both sections.

Given the breadth and heterogeneity of the available literature, this review was designed as a scoping evidence synthesis rather than a meta-analysis or exhaustive taxonomic revision. Differences in study design, sampling approach, diagnostic method, reporting standard and historical nomenclature limited direct comparison across studies. Therefore, the synthesis focused on identifying reported host–tick–microorganism associations, broad ecological and methodological patterns, and major knowledge gaps relevant to wildlife, veterinary and public health contexts.

### 2.3. Data Extraction

Following article selection, the data were extracted on wildlife host species, tick species and life stage where reported microorganisms were detected, and geographical location, sample type, diagnostic method and occurrence data where available. Marsupial nomenclature followed Jackson and Groves [26] with subsequent changes for bandicoots following Baker and Gynther [27]. Geographical data was reported where possible; if not provided, the data was reported at the state level using standard Australian abbreviations: New South Wales (NSW), Queensland (QLD), South Australia (SA), Tasmania (TAS), Victoria (VIC), Western Australia (WA) and the Northern Territory (NT).

Because true prevalence is rarely estimable from heterogeneous wildlife studies, occurrence was reported instead. Tick occurrence was defined as the number of hosts infested with at least one tick divided by the total number of hosts examined, following Bush et al. [28]. Where possible, species-specific tick occurrence was recorded (Table 1 and Table S1). Where studies did not provide tick-level or host-level detail, occurrence was interpreted cautiously and summarised based on the presence of at least one tick species per host or host group. Similarly, microorganism occurrence was defined as the number of positive samples divided by the number of samples tested (Supplementary Table S2). Where multiple diagnostic methods were used, results were extracted separately where available to retain information on method-specific detection patterns.

The included studies employed a wide range of diagnostic approaches, from microscopy and serology to conventional and quantitative PCR, with sequencing and metagenomic methods. As these differ substantially in sensitivity, specificity, taxonomic resolution and detection thresholds, direct comparisons among studies should be interpreted with caution.

## 3. Results and Discussion

### 3.1. Studies on Ticks

A total of 113 studies investigated ticks associated with Australian wildlife, comprising 64 studies focused exclusively on ticks and 49 studies that included data on both ticks and tick-borne microorganisms. These studies examined wildlife hosts, including mammals (studies = 73), reptiles (studies = 24), and birds (studies = 16), with several studies involving multiple host species. Extracted data on wildlife hosts, tick species and life stages, geographical location, identification method, and occurrence are summarised in Supplementary Table S1. Because study designs, sampling methods and reporting detail varied substantially, the results below emphasise broad host–tick patterns and evidence gaps rather than pooled prevalence estimates.

#### 3.1.1. Species of Wildlife

A total of 160 different wildlife species have been examined for tick parasitism in the reviewed studies (Supplementary Table S1). Among these, mammals represented the largest group (*n* = 72), predominantly marsupials, followed by birds (*n* = 67), and reptiles (*n* = 21). Given that Australia is estimated to host over 8000 wildlife species, the current data compiled herein represents only a small fraction of the country’s wildlife diversity. The limited host range reported likely reflects the sampling methods used. Most studies relied on one or more of the following: opportunistic sampling (e.g., zoos, wildlife sanctuaries, or animals presented to veterinary clinics), collection of dead animals (e.g., road-kills), targeted trapping or baiting, and live sampling [29,30,31,32,33]. These approaches introduce constraints that probably contribute to the limited number of species represented. In addition, the purpose of sampling varied between studies—ranging from general health assessments to focused investigations of parasitic burdens, contributing further to variation in host representation [32,34,35,36,37,38].

Additionally, sampling location (i.e., host habitat) would have likely influenced the number of individuals examined in different studies. For instance, trap-based sampling is inherently selective, as animals must be small enough to be trapped, excluding the large wildlife hosts from the population. These factors highlight the challenges associated with estimating prevalence across wildlife populations.

Notably, some host species were reported to harbour a greater diversity of tick species, including short-beaked echidna [39,40,41], brush-tailed possum [35,36,42], and shingleback skink [43,44,45]. However, this apparent diversity may reflect a sampling bias, as these species were more frequently sampled relative to others. More importantly, it may also underscore a potential bias arising from more intensive sampling in certain geographic regions, further reinforcing the need to interpret host–tick associations within the context of spatial distribution. Therefore, the wildlife hosts most frequently reported in the literature should be interpreted as the best-documented hosts rather than necessarily the most important ecological hosts for tick maintenance or microorganism transmission.

#### 3.1.2. Species of Ticks

**Figure 2:**
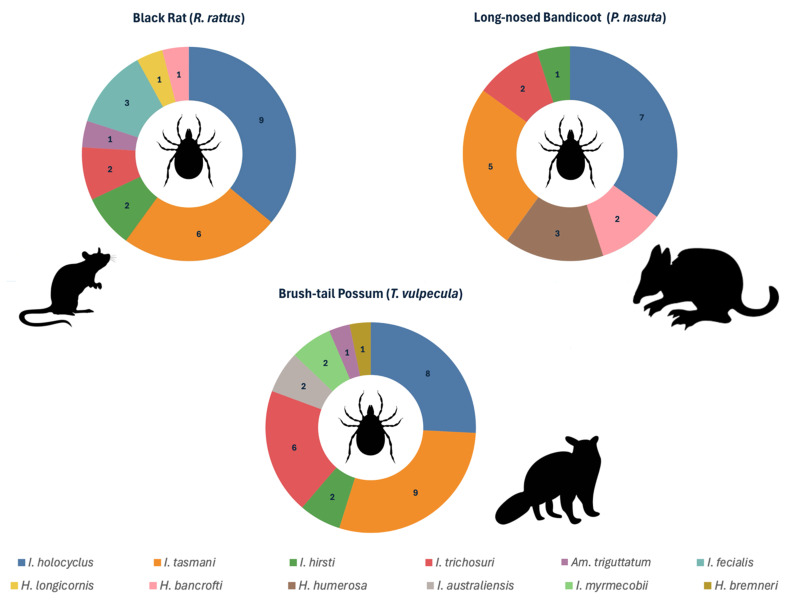
Donut charts illustrating the diversity of tick species reported on key wildlife hosts at the urban wildlife interface. Each colour segment represents a distinct tick species, while the number within each segment indicates how many studies reported that tick species on the corresponding host.

**Figure 3:**
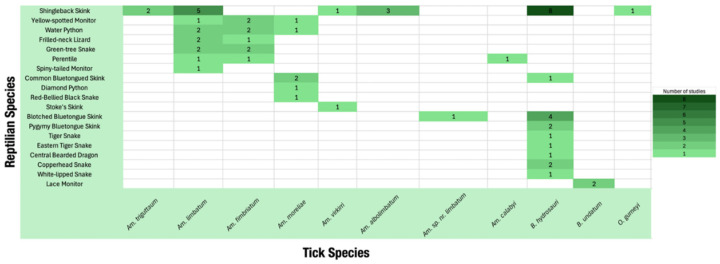
Heatmap illustrating the tick–host associations for reptile species.

Ticks reported on wildlife in the reviewed studies belonged to both the hard (family Ixodidae) and soft (family Argasidae) ticks (Table 1) [36,41,42,46,47,48,49,50,51,52]. Of the 50 species parasitising wildlife, the majority (*n* = 45) were hard ticks, spanning the genera Ixodes, *Amblyomma*, *Haemaphysalis*, *Bothriocroton* (formerly *Aponomma*) and *Rhipicephalus* (Table 1) [36,41,42,46,50,51,52,53,54]; only five soft-tick species, in *Ornithodoros* and *Argas*, were reported (Table 1) [45,55,56,57]. These 50 species represent two-thirds of the 74 currently recognised in Australia [2]. As with the underrepresentation of wildlife hosts, the absence of the remaining tick species may be merely due to limitations in sampling scope, methods used, and geographical area covered. Moreover, certain wildlife species are restricted to distinct ecological niches, resulting in tick species being restricted to highly specific environments, particularly in the case of host-specific ticks. Notable patterns were observed regarding host–tick associations (Table 1, Table S1, Figure 2 and Figure 3).

**Table 1:** Tick species reported on Australian wildlife, organised by genus and ranked by the number of wildlife host species parasitised (from highest to lowest). Tick–host associations are colour-coded to indicate vertebrate host class: mammals [red], birds [blue], and reptiles [green]. The number in brackets represents the number of confirmed host species of each vertebrate class.

Genus	Species	Selected Reference(s)
*Ixodes*	*I. holocyclus* [31, 23], *I. tasmani* [35], *I. hirsti* [9, 22], *I. fecialis* [15], *I. trichosuri* [12], *I. australiensis* [11], *I. cornuatus* [5, 1], *I. eudyptidis* [4], *I. laridis* [4], *I. antechini* [3], *I. myrmecobii* [3], *I. victoriensis* [3], *I. woyliei* [3], *I. kohlsi* [3], *I. confusus* [2], *I. simplex* [2], *I. heathi* [1], *I. cordifer* [1], *I. ornithorhynchi* [1], *I. barkeri* [1]	[41,42,47,48,52,53]
*Amblyomma*	*Am. triguttatum* [18, 1], *Am. limbatum* [7], *Am. fimbriatum* [5], *Am. moreliae* [5], *Am. vikirri* [2], *Am. albolimbatum* [1], *Am. australiense* [1], *Am. echidnae* [1], *Am. cyprium cyprium* [1], *Am.* sp*. nr. limbatum* [1], *Am. calabyi* [1]	[46,54,58,59]
*Bothriocroton*	*B. hydrosauri* [1,9], *B. tachyglossi* [2], *B. concolor* [1], *B. auruginans* [1], *B. undatum* [1]	[50]
*Haemaphysalis*	*H. bancrofti* [16], *H. humerosa* [8], *H. longicornis* [6], *H. ratti* [5], *H. petrogalis* [2], *H. lagostrophi* [2], *H. bremneri* [2]	[50,60]
*Rhipicephalus*	*Rh.* (*sanguineus*) *linnaei* [1]	[36]
*Ornithodoros*	*O. gurneyi* [1], *O. capensis* [1]	[45,55]
*Argas*	*Ar. robertsi* [10], *Ar. dewae* [2], *Ar. lagenoplastis* [1]	[56,57,61,62]

For example, mammalian species were most commonly infested with *Ixodes tasmani* and *I. holocyclus* (Table 1), birds were mostly associated with *I. hirsti* and *I. holocyclus* (Table 1), whereas *Amblyomma limbatum* and *Bothriocroton hydrosauri* were mainly associated with reptiles (Table 1 and Figure 3) [32,36,38,46,50,51,63,64].

Many of the species described have been recognised for decades. However, several species have been either recently described—such as *I. barkeri*, *I. woyliei*, *I. heathi*, *Amblyomma* sp. *near limbatum*, *I. laridis*—or recently redescribed, including *I. confusus* and *I. victoriensis* [41,42,47,48,49,52,65]. As a result, the host ranges and ecological roles of these species are only beginning to be characterised. Given the ongoing taxonomic revision and increasing application of molecular methods, it is likely that future research will identify new tick species or describe novel host–tick associations in Australian wildlife.

Some studies were able to determine and report tick occurrence at the level of individual hosts (Supplementary Table S1). However, many reported only the total number of ticks collected across all sampled wildlife, without linking those counts to individual hosts—thereby making it challenging to calculate the prevalence (Supplementary Table S1). Although information on total tick counts can be helpful for estimating sample size requirements and studies on microorganism testing, they are of very limited use for estimates of potential prevalence or burden variability within host populations. Therefore, where practical, future studies should aim to estimate overall tick counts with individual-level infestation data, thus allowing for more accurate assessments of occurrence/prevalence. Accordingly, the host–tick associations summarised here should be interpreted as reported records rather than complete host ranges, particularly for under-sampled wildlife groups and regions.

Across the reviewed studies, the recorded tick fauna was dominated by the genus *Ixodes*, which accounted for roughly half of all tick species reported on wildlife (around 20 species), followed by *Amblyomma* (11), *Haemaphysalis* (7) and *Bothriocroton* (5), with the soft-tick genera *Argas* (3) and *Ornithodoros* (2) and a single *Rhipicephalus* species making up the remainder. Host breadth was similarly uneven: a small number of generalist species accounted for a disproportionate share of host records, with *I. holocyclus*, *I. tasmani* and *I. hirsti* reported from the widest range of wildlife hosts (Table 1), while many species were each recorded from only one or two hosts. Most tick species were reported from a single vertebrate class; a few generalists, notably *I. holocyclus*, spanned mammals and birds, whereas reptile-associated records were dominated by *Amblyomma* and *Bothriocroton*. These distributions should be read as reflecting research effort and host accessibility rather than true host specificity, as the most frequently reported ticks were also those recovered from the most intensively sampled hosts and regions.

#### 3.1.3. Identification of Ticks

**Figure 4:**
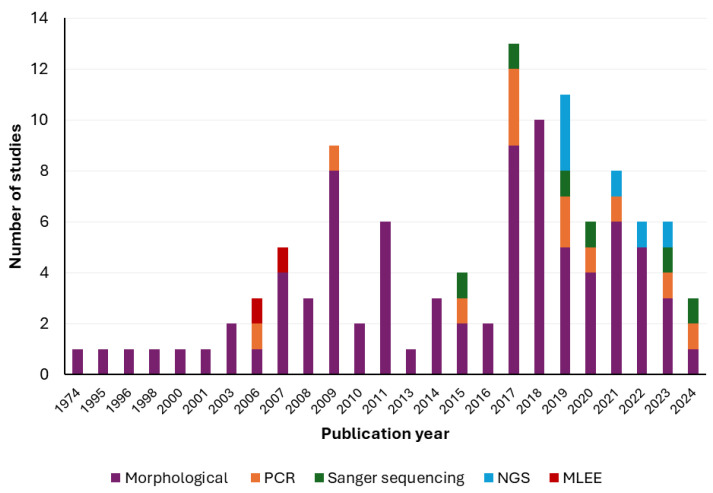
Stacked bar chart illustrating the overall timeline of diagnostic techniques used to identify ticks found on wildlife species. MLEE (Multi-locus Enzyme Electrophoresis).

Morphology remains the principal method for identifying tick species, used in 85 of the 113 studies reviewed (Supplementary Table S1, Figure 4). The most commonly used approach involved microscopy (stereomicroscopy or light microscopy) using F.H.S. Roberts dichotomous keys [66]. This had remained consistent for several years, until a revision of the dichotomous keys was published by Barker and Walker [4,5]. These were also used in the more recent studies [31,32,34,36,37,38,51,55,67,68,69]. Morphological identification has several limitations. Morphological characteristics can be very similar amongst tick species (e.g., within the genus *Haemaphysalis*) making accurate identification difficult. Also, immature stages of ticks (nymphs, larvae) have been difficult to identify to species-level [35,43,46,50,55,69,70,71,72,73,74,75,76], leaving many researchers often reporting to genus level. Furthermore, the availability of intact specimens is a prerequisite for accurate morphological identification; thus, any damage to specimens can significantly compromise this approach [77]. In addition, expertise in classical parasitology is also becoming limited and although many of these studies used the services of microscopy experts, identification errors remain possible, particularly for untrained personnel.

Due to these limitations, molecular methods are increasingly being used, albeit in a smaller proportion of studies reviewed herein (15 of 113) (Supplementary Table S1, Figure 4). A number of techniques have been used including conventional PCR (cPCR) [32,42,51,78,79,80,81,82,83,84], Sanger sequencing [32,37,42,80,84,85], and next-generation sequencing (NGS) [33,51,76,83,85,86]. These methods typically use mitochondrial or ribosomal markers such as cytochrome *c* oxidase I subunit (COI) and 16S ribosomal RNA (rRNA); although many other genetic markers (e.g., 16S rRNA gene, 12S rRNA gene, ITS-1, ITS-2) are also available and have been explored in recent studies in other parts of the world [87]. Mitochondrial markers, particularly COI and 16S rRNA loci, have been widely used for species-level identification and phylogenetic investigations of ticks. More recently, complete mitochondrial genome sequencing has helped resolve taxonomic uncertainty among morphologically similar species, offering improved resolution over shorter loci alone. Barker and colleagues [1,2,4,5,41,52] have contributed extensively to this work, clarifying the evolutionary relationships, taxonomy and biogeography of Australian ticks and highlighting the value of integrated morphological and genomic approaches.

Molecular approaches are particularly useful for identifying immature tick stages, including larvae and nymphs, which are often difficult to reliably distinguish morphologically. For example, nymphs of *I. hirsti* and *I. trichosuri* cannot be distinguished through morphological techniques. Moreover, a key advantage of molecular techniques is their ability to detect both tick species and potential microorganisms simultaneously, as demonstrated in studies using NGS [86]. Furthermore, molecular data permit the identification of cryptic tick species—species which are morphologically indistinguishable yet genetically distinct [88]. For instance, McCann et al. [85] identified potential cryptic *Ixodes* species on bush rats. Other advantages of molecular identification include species-level identification, establishing evolutionary relationships and the demonstration of novel species, such as the identification of a novel *Cryptocroton* species [89]. Taxonomic revision has been most consequential for morphologically conserved or immature specimens: the genus *Haemaphysalis* is difficult to separate on morphology alone, nymphs of *I. hirsti* and *I. trichosuri* are morphologically indistinguishable, and molecular data have revealed cryptic Ixodes diversity on bush rats [85], as well as supporting the recent description or redescription of *I. barkeri*, *I. woyliei*, *I. heathi*, *I. confusus* and *I. victoriensis* [41,42,47,48,49,52]. These cases illustrate where reliance on morphology alone would have understated Australian tick diversity.

Despite these strengths, molecular methods present challenges. For instance, they require optimised primers and reference sequence databases. Despite becoming increasingly accessible, molecular approaches still require specialised laboratory infrastructure, bioinformatic expertise, and curated reference databases for reliable interpretation [90,91].

Only two studies [53,71] used multi-locus enzyme electrophoresis for tick identification (Supplementary Table S1, Figure 4). This technique, although rarely used, has been previously used for ticks and other ectoparasites, and has demonstrated success in identifying immature stages of ticks, genotypic diversity, as well as serving as an aid to identify morphologically similar ticks [92,93].

In addition to identification methods, the collection site on the host can also affect tick detection. Ticks may exhibit host-specific attachment preferences, and, if not thoroughly examined, can lead to underreporting. For example, *I. holocyclus* was more commonly found on the heads of black rats and on the limbs of long-nosed bandicoots [94,95]. Additionally, different tick species may also differ, where they attach on a single host species [94]. For example, male ticks of *Bothriocroton* (*Aponomma*) *hydrosauri* and *Am. albolimbatum* had different preferences, strongly influenced for breeding purposes [94]. A thorough, systematic examination of hosts is therefore essential for accurate tick collection data. Because ticks act as disease vectors, identification errors can misrepresent host–parasite relationships and mislead transmission and surveillance inferences, which is a particular risk given the limited wildlife data available in Australia.

Given the dynamic aspects of tick taxonomy with frequent re-classifications, reference specimens should be archived in reference collections such as those held in museums. This process will facilitate any future verifications, support phylogenetic analyses, and maintain updated host records, particularly with advancements in molecular technologies [40,49,52,58]. Since there are advantages and limitations of both morphological and molecular approaches, an integrated strategy—combining both methods where feasible—should be employed for robust and reliable tick identification in Australian wildlife investigations.

Overall, the increasing integration of molecular tools has substantially improved the detection of cryptic tick diversity, clarified host–tick associations, and enhanced the identification of microorganisms within wildlife-associated ticks. However, considerable variation remains in the genetic markers, sequencing platforms, and analytical approaches used across studies, limiting direct comparisons between datasets. Future studies would benefit from standardised molecular workflows, expanded reference databases, and the integration of morphological and genomic approaches to strengthen the taxonomy and epidemiology of Australian ticks and their associated microorganisms.

#### 3.1.4. Pathogenic Effects of Ticks

The majority of the pathogenic significance of ticks is indirect and relates to their ability to transmit pathogenic microorganisms. However, ticks can also elicit direct pathogenic effects. In wildlife contexts, only a limited number of studies have reported such direct effects caused by ticks themselves [30,38,50,96,97,98,99,100]. For example, anaemia has been reported in wombats due to tick burden [50]. Experimental studies have also demonstrated anaemia from heavy tick infestation in northern brown bandicoots and koalas, with consistent outcomes between captive and wild animals [101,102]. Such findings are unsurprising, as anaemia, irritation and dermatitis are well-recognised consequences of tick infestation.

In addition, tick paralysis caused by *I. holocyclus* has been reported in several wildlife species, including birds, flying foxes and koalas, with mortality documented in some cases [38,96,98,100]. From a veterinary perspective, tick paralysis is a medical emergency requiring immediate intervention, as clinical signs often advanced by the time animals are found. Signs in wildlife resemble those in domestic animals, supporting thorough examination for paralysis ticks and prompting supportive treatment as used in companion animals [38,98,103]. There are several reasons why direct pathogenicity is reported infrequently in wildlife. Many wildlife and associated tick species exist in endemic cycles, and repeated exposure may sometimes contribute to some level of acquired immunity, thus resulting in reduced tick infestations [104]. Moreover, host defence behaviours such as grooming and scratching may also contribute to reducing tick burdens in some wildlife species. Despite these adaptations, there is limited understanding of the true extent of the direct pathogenic effects of ticks in Australian wildlife and it requires thorough systematic health assessments along with longitudinal studies.

Overall, available evidence suggests that direct pathogenic effects of ticks on Australian wildlife are currently poorly characterised, partly due to difficulties associated with monitoring free-ranging wildlife populations and distinguishing primary tick-associated pathology from concurrent disease processes or environmental stressors. Most reviewed studies have focused on heavily parasitised or clinically affected animals encountered opportunistically through wildlife rehabilitation or veterinary investigations, potentially biassing current understanding toward severe presentations. Consequently, the broader ecological and population-level impacts of tick parasitism on Australian wildlife remain poorly understood and warrant further investigation using longitudinal and multidisciplinary approaches.

#### 3.1.5. Wildlife Tick Epidemiology and Ecology

Much of the ecology of Australian tick species remains poorly understood. As a result, much of the available information is either presumed or extrapolated from related tick species [2]. Numerous factors may influence a given tick species and its population dynamics. These include abiotic factors (e.g., temperature, humidity, moisture) and biotic factors (e.g., vegetation, hosts), which interact in complex ways. Each factor can exert a distinct influence, and it is often challenging to estimate the cumulative impact of all factors on tick burdens of wildlife hosts [105].

Some studies have investigated seasonal variation in tick populations [37,53,106,107,108,109]. These studies suggest that tick life stage predominance varies by season and geographical region [37,53,106,107,108,109]. For example, in north–east and eastern Australia, larvae were predominant in autumn and adults in winter [37,107]. Such patterns are likely attributable to diverse climatic zones within Australia which influence the abiotic factors in conjunction with varied requirements of different tick life stages. A more recent study reported nymphs and larvae of various *Ixodes* spp. as the predominant stages found in black rats during winter season [68]. However, most studies on seasonality were conducted in the early 2000s, highlighting the need for further investigations under current climatic conditions.

Because ticks cannot travel far unaided, dispersal depends largely on passive transport by hosts during blood-feeding, and may be most pronounced on flying hosts that cover long distances quickly. Several studies documented tick presence on flying hosts, including flying foxes [36,64,98,100,110] and birds [38,39,48,55,56,61,63,78,79,82,111,112,113]. Differences in feeding behaviour, host association, attachment duration, and host-seeking ecology may all influence the dispersal potential of different tick species [114]. For example, some hard ticks remain attached to hosts for prolonged periods during feeding, potentially facilitating passive dispersal over greater distances, whereas other tick species exhibit more nidicolous behaviours and may disperse less extensively. The relative contribution of flying vs. terrestrial hosts in tick dispersal and ecology in Australia needs further investigations. Additionally, migratory birds that travel to Australia from the Northern Hemisphere may also be carrying new tick species and pathogens to this country.

Several factors have been proposed to explain variation in tick burden among individual hosts. Lydecker et al. [68] found that black rats with a higher body condition were associated with a greater tick burden, compared to those with a lower body condition. Similarly, differences in tick burden between male and female hosts have also been reported as in the case of male rabbits which have comparatively a higher tick burden, although no such sex-based differences were observed in black rats [37]. These patterns may reflect behavioural differences, such as increased movement or mate-seeking in males, leading to greater exposure to ticks. Additionally, larger hosts logically provide greater surface area and blood volume, supporting higher tick loads. Identifying individual- and population-level risk factors for elevated tick burdens may enhance understanding of tick dynamics in wildlife populations.

Most tick species recorded on wildlife were hard ticks, many of which exhibit a three-host life cycle. However, host specificity varies considerably among Australian tick species, with some exhibiting relatively broad host associations whereas others appear more host- or habitat-associated. The role of each host in tick population maintenance and transmission is yet to be fully understood. Previously established tick–host relationships (e.g., *I. holocyclus* and bandicoots) have been challenged, with suggestions that bandicoots may not be the primary hosts [115]. As research progresses, it will be essential to map host distributions, identify the life stages most commonly associated with each host, and clarify whether hosts act as dispersal vehicles, maintenance reservoirs or pathogen carriers. Notably, due to the broad host associations reported for some tick species, host roles cannot be generalised without considering local environmental and ecological contexts [115].

Collectively, wildlife-associated tick ecology in Australia reflects complex interactions among host availability, habitat, climate and tick life-history traits, but interpretation is constrained by variation in sampling intensity, geographic coverage, hosts examined and methods across studies. Most available data originate from eastern and south-eastern Australia, while large regions of northern and central Australia remain comparatively underrepresented. In addition, many studies relied on opportunistic or cross-sectional sampling designs, limiting inference regarding seasonal dynamics, long-term ecological trends, and vector–host interactions. Consequently, current understanding of Australian wildlife tick ecology remains fragmented, highlighting the need for longitudinal, standardised, and geographically representative investigations integrating ecological, molecular, and epidemiological approaches.

#### 3.1.6. Public Health and Companion Animal Considerations

Currently, 23 of the 74 tick species identified in Australia are known to bite humans [1,4,6,116,117,118]. In recent years, there has been increasing recognition and reporting of a broader diversity of Australian tick species biting humans, including *Amblyomma limbatum*, *Bothriocroton hydrosauri*, *Amblyomma albolimbatum*, and *Ixodes australiensis* [116,117,118]. However, the majority of reported human tick bites in Australia continue to be associated with *Amblyomma triguttatum* and *Ixodes holocyclus* [1,4,6]. Pathogenic effects in humans resulting from tick bites include localised reaction at the bite site (irritation, pruritus), tick paralysis (especially in children), alpha-gal syndrome (mammalian meat allergy), and potential transmission of tick-borne pathogens [4,6,119]. Exposure typically occurs during outdoor activities in areas where wildlife and ticks are found; however, urban environments are not exempt as wildlife species often encroach into these areas, bringing ticks to people as well as domestic animals.

Urban expansion encroaches on wildlife habitats and increases contact between people, pets and urban-adapted wildlife [120,121]. Such interactions can harm wildlife (e.g., pet attacks, vehicle collisions) [16], yet cities also offer abundant food that allows some species to persist or thrive [121,122].

Eight studies investigated tick infestation in wildlife found in or near urban environments [33,35,37,51,64,123,124,125]. Sampling has concentrated in eastern and south-eastern states, with northern and central regions relatively underrepresented. These studies consistently identified the black rat, brush-tailed possum, and long-nosed bandicoot as common urban hosts. Furthermore, a diverse array of tick species was detected on these hosts, many of which are known to bite humans and companion animals, thus highlighting their public and veterinary health importance [1,4,6].

Patterns across urban and peri-urban environments were inconsistent. Webster et al. [123] found a higher prevalence of *I.* *trichosuri* on urban than bushland brush-tailed possums in New South Wales, while *I. tasmani* occurrence was similar across both, though the larger urban sample may have influenced these estimates [123]. By contrast, Hillman et al. [124] found no ticks on urban possums in Western Australia—possibly a seasonal trapping artefact—and higher tick burdens in quendas from bushland than urban areas. Together these findings point to strong geographical variation in urban tick–wildlife dynamics [124].

Further studies are warranted to demonstrate how geographical and anthropogenic factors influence wildlife–tick interactions and their ecology. Taylor et al. [33] demonstrated that maintaining backyards (e.g., vegetation trimming) and the presence of outdoor pets may deter wildlife, such as bandicoots, from entering residential areas-thereby reducing tick exposure. Consequently, future research should also examine the role of human modifications, urban planning, and resource availability in shaping wildlife movements and the translocation of ticks, ultimately influencing the risk of tick encounters for humans and domestic animals.

Collectively, these studies suggest that urban and peri-urban wildlife may contribute to maintaining tick populations at the human–domestic and animal–wildlife interface, particularly in regions where adaptable wildlife species persist within modified environments. However, substantial geographic variation exists in reported tick burdens and host associations, and relatively few studies have systematically compared urban, peri-urban, and natural ecosystems using standardised methodologies. In addition, the extent to which wildlife-associated ticks contribute to pathogen transmission within urban environments remains incompletely understood. Further integrated ecological and epidemiological investigations are required to better characterise how urbanisation, habitat modification, wildlife movement, and human activity influence tick ecology and potential public health exposure in Australia.

### 3.2. Studies of Tick-Borne Microorganisms in Australian Wildlife

**Figure 5:**
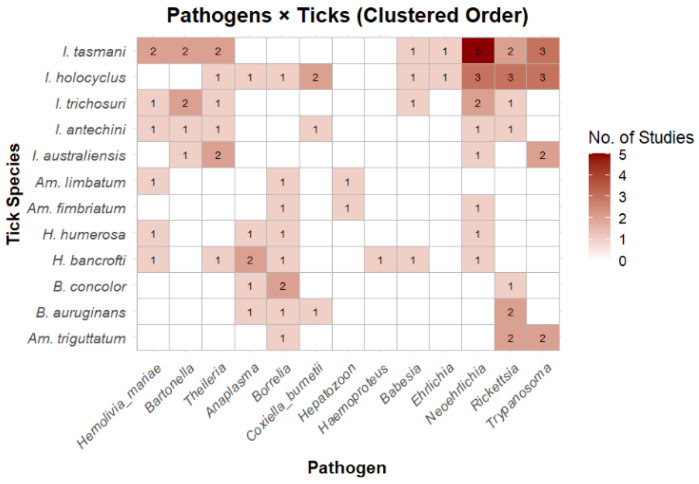
Heatmap demonstrating tick-borne microorganism associations of tick species.

**Figure 6:**
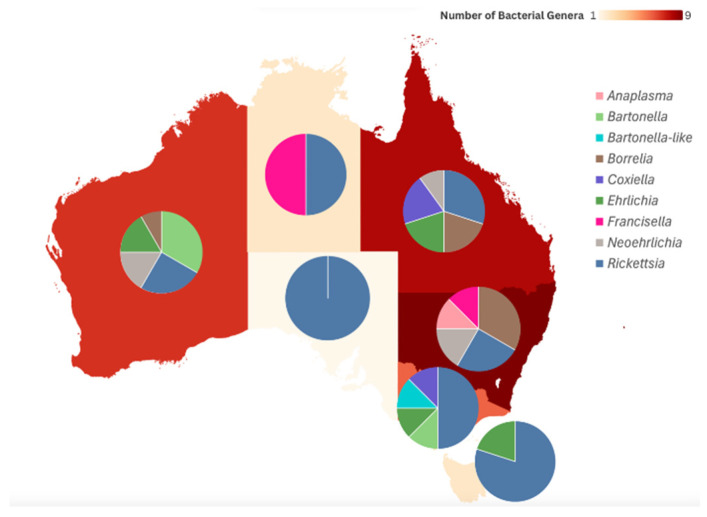
Map of Australia comparing the diversity of bacterial genera. For each Australian state, a pie chart is present illustrating the relative proportion of the most commonly reported genera (up to five different genera).

As of March 2024, a total of 69 studies have investigated the presence of microorganisms within various tick species parasitising Australian wildlife, with relevance to both human and domestic animal health (Supplementary Table S2, Figure 5, Table 2, Table 3, Table 4, Table 5, Table 6 and Table 7). These microorganisms include bacteria, protozoa, viruses, or combinations thereof, and have been detected across diverse tick taxa. Additionally, some studies have reported microorganisms such as *Babesia* and *Theileria*, which are known to be obligately transmitted by ticks, even when a specific tick vector was not identified in the study (Table S2, Table 2, Table 3, Table 4, Table 5, Table 6 and Table 7, Figure 5 and Figure 6). Of note, *Trypanosoma* species have also been included in this review. Although ticks have not been definitively confirmed as vectors of *Trypanosoma* in Australia, accumulating evidence suggests a potential role in their transmission.

The reviewed evidence was unevenly distributed across microorganism groups and host taxa. Bacteria were the most frequently investigated group (31 studies), followed by protozoa (24) and viruses (10); these totals exceed the number of microorganism studies overall because several studies reported more than one group. Among bacteria, *Rickettsia* was by far the most frequently reported genus (20 studies), while among protozoa *Theileria* (12 studies) and *Babesia* (10) predominated. Microorganism diversity was concentrated in the most intensively studied ticks and hosts: at the genus level, *Ixodes* harboured the widest range of microorganism genera, followed by *Haemaphysalis* and *Amblyomma*, with soft ticks yielding the fewest. By host group, mammalian wildlife—particularly bandicoots, possums, rodents and macropods—accounted for the greatest microorganism diversity, whereas avian records were dominated by tick-associated viruses and reptilian records by *Rickettsia*, *Borrelia* and *Hepatozoon*. As with the tick data, these patterns track sampling intensity and diagnostic effort and should not be interpreted as measures of true infection prevalence or vector importance.

#### 3.2.1. Bacteria

Thirty-one studies have reported bacterial species detected in ticks associated with Australian wildlife (Figure 6). This section provides an overview of the key bacterial genera identified, with emphasis on those of recognised veterinary and/or public health importance within the Australian context.

##### Anaplasmataceae

*Anaplasma* species are tick-borne intracellular bacteria that infect haematopoietic cells of animals and humans, causing anaplasmosis [126]. In Australia the disease is of veterinary significance in cattle and dogs. *Anaplasma marginale*, transmitted by *Rh. australis* (formerly *Boophilus microplus*), causes ‘tick fever’ in cattle in Northern regions of Australia. Anaplasmosis in cattle is a significant disease from an economic and welfare perspective as it can cause severe clinical diseases (e.g., anaemia, jaundice) and decreased production (milk, reduced weight gain) [127].

*Anaplasma platys* (formerly *Ehrlichia platys*) is responsible for canine infectious cyclic thrombocytopaenia [128]. Fortunately, subclinical and mild clinical disease has only been seen in dogs in Northern regions of Australia, particularly near indigenous communities [128,129]. *A. platys* may also pose a risk for indigenous communities as a potential cause for mild illness (e.g., headaches) in people but has not been proven to cause disease in humans yet [130]. The vector for *A. platys* has not been firmly established, but there is reasonably strong suspicion that *Rh.* (*sanguineus*) *linnaei* may be responsible [128].

Five studies included in this review reported *Anaplasma* species present either in ticks associated with wildlife or within wildlife host tissues (Table 2). Notably, studies testing ticks did not examine host blood or tissue; consequently, these are detections in ticks only—neither host infection nor vector competence was established. Further research is needed to investigate transmission dynamics and the pathogenic potential of these *Anaplasma* species, particularly as a novel *Anaplasma* genotype was identified in *Amblyomma triguttatum* removed from a human patient [131].

Gofton et al. [31] detected *Anaplasma bovis* within a questing *H. bancrofti* in Kioloa, NSW, a region also inhabited by wildlife species. The same research group previously reported *A. bovis* in *Am. triguttatum* in Western Australia (Table 2) [132]. Although *A. bovis* causes bovine anaplasmosis in Africa, Asia, and Europe, clinical disease has not yet been documented in Australian livestock [133]. Importantly, *A. bovis* has also been identified in the tissues of a horse [134], and *A. bovis*-like sequences have been detected in *Haemaphysalis* spp. (Table 2). While the zoonotic potential remains unknown, reports of disease potentially attributed to *A. bovis*-like species were found in USA [135]. Further studies will be needed to determine whether *A. bovis* is a threat to the health and welfare of Australian cattle, and whether *H. bancrofti* is a competent vector.

**Table 2:** *Anaplasma* spp. detected in Australian tick and/or wildlife sample.

Species	Host/Vector	Reference
*Anaplasma* sp.	Echidna (*Bothriocroton concolor*)	[36]
*A. bovis*-like sp.	Long-nosed Bandicoot (*Haemaphysalis bancrofti*, *H. humerosa*)	
	Red-necked Wallaby (*H. bancrofti*)	
*Anaplasma* sp.	Bare-nosed wombat (*B. auruginans*)	[50]
*Anaplasma* sp.	Unspecified (*Ixodes holocyclus*)	[39]
*A. platys*	Deer (tissue)	[51]
*A. bovis*	Questing (*H. bancrofti*)	[31]

*Anaplasma platys* was also found in the tissues of wild deer in Conondale, QLD (Table 2) [51]. The epidemiological role of wild deer in *A. platys* transmission is currently unclear. They may act as reservoir hosts or may represent the incidental spillover infections. Although *Rh.* (*sanguineus*) *linnaei* is the presumed vector of *A. platys*, it has not been reported on wild deer, and none of the reviewed studies included tick sampling from these animals. Thus, the involvement of other tick species in the transmission cycle remains plausible. Further studies are required to clarify the identity of the vector(s), their host ranges, and the role wild deer may play in the ecology and epidemiology of *A. platys* in Australia.

In addition to *Anaplasma* species, members of the genera *Ehrlichia* and *Neoehrlichia* have also been detected in Australian wildlife ticks. Gofton et al. [132] identified novel *Ehrlichia* and *Anaplasma* genotypes in *Amblyomma triguttatum* collected from geographically distinct regions of Australia, highlighting the diversity of tick-associated Anaplasmataceae present within Australian ecosystems. Subsequent molecular investigations have also identified *Neoehrlichia*-like organisms and additional novel Anaplasmataceae taxa in wildlife-associated ticks using 16S rRNA gene sequencing and next-generation sequencing approaches [36,131]. However, the ecology, host associations, vector competence, and pathogenic significance of many of these organisms remain poorly understood, and most have not yet been formally characterised beyond molecular detection.

##### *Borrelia*  

The genus *Borrelia* comprises diverse spirochaetal bacteria associated with arthropod vectors, including ticks and lice, and contains species of considerable medical and veterinary importance worldwide [136]. Members of the *Borrelia burgdorferi sensu lato* complex are responsible for Lyme borreliosis in North America, Europe, and parts of Asia, whereas relapsing fever group borreliae are associated with recurrent febrile illness in humans and animals [136]. In Australia, *Borrelia* has attracted considerable attention due to ongoing discussion surrounding tick-associated illness and DSCATT; however, there remains no evidence supporting the endemic presence of Lyme borreliosis-causing *Borrelia burgdorferi sensu lato* in Australia [24,25]. Nevertheless, several genetically distinct indigenous *Borrelia* lineages have been identified in Australian wildlife and wildlife-associated ticks, highlighting the ecological diversity of this genus within Australian ecosystems. From a veterinary perspective, *Borrelia anserina* and *Borrelia theileri* are historically recognised exotic species associated with domestic poultry and cattle, respectively [136].

Eight studies detected *Borrelia* spp. in wildlife tissues or their ticks (Table 3); none demonstrated tick-to-host transmission or associated pathology. Further work will be needed to determine the vector potential of the ticks for these *Borrelia* species and whether any of these wildlife species show disease or serve as reservoir hosts.

**Table 3:** *Borrelia* spp. detected in Australian tick and/or wildlife (ticks in parentheses).

Species	Host/Vector/Tissue	Reference
*Ca.* Borrelia tachyglossi	Echidna (*B. concolor*)	[137]
	Northern brown Bandicoot (*Haemaphysalis humerosa*)	[36]
*Borrelia* sp.	No host (*H. bancrofti*)	[31]
*Borrelia* sp.	Black Rat (tissue)	[51]
	Bush Rat (tissue)	
	Swamp Rat (tissue)	
*Borrelia* sp. closely related *to Ca.* Borrelia ivorensis	Bare-nosed Wombat (*B. auruginans*)	[50]
*Ca.* Borrelia undatum	Lace Monitor (*B. undatum*)	[54]
*Borrelia* spp.		[81]
*Ca.* Borrelia rubricentralis	Perentie (*Amblyomma calabyi*)	[54]
*Borrelia* sp.	Perentie (*Am. limbatum*, *Am. fimbriatum*)	[54]
*Borrelia* sp.	Echidna (*Ixodes holocyclus*)	[39]

Importantly, none of the *Borrelia* species discovered were genetically related to the Lyme borreliosis group. However, there was a *Borrelia* species within the relapsing fever group detected in a questing *H. bancrofti* in Sydney, NSW [31]. The study had also simultaneously collected tissues of rodents and brush-tailed possums in the same area but was unable to find the same *Borrelia* species within the tissues [31]. An additional *Borrelia* species found within the relapsing fever group was in *I. holocyclus* ticks which had originated from an echidna [39]. The relapsing fever group of *Borrelia* has been reported to cause flu-like symptoms in humans and has been reported to be a diagnostic challenge for medical doctors [138]. Further studies will be needed to determine their pathogenic significance.

*Borrelia queenslandica* was first reported in the long-haired rat (*Rattus villosissimus*) in Queensland in 1956 [139]; however, there have been no further reports of this species in any of the studies reviewed herein. Moreover, none of the three known species of *Borrelia* in Australia nor *Bo. burgdorferi sensu lato* were found in these studies. As a result, the role of Australian wildlife in the epidemiology of *Bo. theileri* or *Bo. anserina* remains unresolved.

Collectively, available evidence indicates that Australian wildlife and wildlife-associated ticks harbour a genetically diverse assemblage of indigenous *Borrelia* lineages distinct from the classical Lyme borreliosis group. However, the ecology, transmission dynamics, host specificity, and pathogenic significance of many Australian *Borrelia* species remain poorly understood. Most reviewed studies have relied on the molecular detection of relatively short genetic loci, limiting taxonomic resolution and phylogenetic interpretation. Furthermore, the detection of *Borrelia* DNA within ticks or wildlife hosts alone does not confirm vector competence or reservoir host status. Recent advances in genomics and phylogenetics are increasingly improving the understanding of Australian *Borrelia* diversity, although further studies integrating genomics, wildlife ecology, vector competence investigations, and pathogen isolation are required to clarify their ecological and potential public health significance.

##### *Coxiella*  

The genus *Coxiella* contains a single recognised species, *Coxiella burnetii*, an obligate intracellular bacterium responsible for Q fever in humans [140]. Q fever can lead to clinical disease in people, causing flu-like symptoms, abortion in pregnant women, meningitis, and/or myocarditis [141]. Human infections typically occur through the inhalation of contaminated aerosols, particularly among high-risk groups such as abattoir workers, veterinarians, and farm personnel exposed to infected domestic animals [140]. *Coxiella burnetii* can also cause coxiellosis in cattle, sheep, and goats which can lead to abortion, but many infections remain subclinical. Although many infections with *Coxiella burnetii* remain subclinical in animals, wildlife species and ticks may contribute to the maintenance and environmental dissemination of this organism [140]. Consequently, wildlife-associated ticks have been investigated as potential components of *C. burnetii* transmission cycles in Australia.

Seven studies have reported the presence of *C. burnetii* and other *Coxiella*-like species associated with wildlife or ticks (Table 4). Cooper et al. [142] was the predominant study which focused on detecting *C. burnetii* in a number of wildlife species and their ticks. The authors found a large number of samples that contained *C. burnetii* in the blood, sera, or tick species pertaining to individual wildlife hosts [142]. Of note, Northern Brown Bandicoots and Eastern Grey Kangaroos both had positive samples from ticks and blood samples. The authors interpreted concurrent tick and blood positivity as suggestive of transmission by I. holocyclus and *Am. triguttatum*, though such detections do not establish vector competence. I. holocyclus has been proposed as a Q fever vector on the basis of *C. burnetii* antibodies in known tick-bite cases [143]; however, seropositivity indicates only prior exposure and does not confirm tick-borne transmission, which may occur by other routes. Given the significance of both *I. holocyclus* and *Am. triguttatum* as human-biting ticks, further studies are needed to clarify their vector potential for Q fever and coxiellosis. Moreover, studies should also be conducted to determine the vector potential of other reported tick species (Table 4).

Several studies also identified the presence of *Coxiella*-like endosymbionts in ticks (Table 4). Advances in molecular diagnostics have facilitated their detection, yet the clinical significance of these endosymbionts remains unclear. Given that *C. burnetii* likely evolved from an ancestral *Coxiella*-like organism [144], it is important to genetically characterise these related species. performing so would not only help clarify their evolutionary relationships but also assess any potential pathogenicity. Furthermore, accurate differentiation between *Coxiella*-like species and *C. burnetii* is crucial, as misidentification could lead to overestimation of disease risk [145]. Additional studies comparing these *Coxiella*-like organisms with *C. burnetii* are necessary to establish whether they pose any threat to animal or human health [145].

**Table 4:** *Coxiella burnetii* and other *Coxiella*-like species detected in Australian tick and/or wildlife (ticks in parentheses).

Species	Host/Vector/Tissue	Reference
*C. burnetii*	Brush-tailed Possum (blood)	[142]
	Northern Brown Bandicoot (*Ixodes holocyclus,* blood, sera)	
	Agile Wallaby (blood, sera)	
	Black-striped Wallaby (blood, sera)	
	Red Kangaroo (blood)	
	Eastern Grey Kangaroo (*Amblyomma triguttatum*, blood, sera)	
	Rufous Bettong (sera)	
	Common Wallaroo (blood)	
*Coxiella* sp.	Bare-nosed Wombat (*B. auruginans*)	[72]
*C. burnetii*		[50]
*Coxiella-*like sp.		[32]
*C. burnetii*	Western-barred Bandicoot (*Haemaphysalis humerosa*)	[60]
*Coxiella-*like sp.	Echidna (*B. concolor*)	[36]
*Coxiella* sp.	Red Fox (*H. longicornis*)	
*Coxiella*-like sp.	Mountain Brush-tailed Possum (*I. trichosuri*)	[32]
*Coxiella-*like sp.	Mixed Black Noddy (*Ornithodoros capensis*)	[55]
*C. burnetii*	Kookaburra (*I. holocyclus*)	

##### *Rickettsia*  

*Rickettsia* are obligately intracellular, arthropod-transmitted bacteria; of 27 described species, 17 are human pathogens, typically causing fever, headache and eschar or papule formation [146]. Dogs are the only veterinary species affected by the same species as humans, most notably *R. rickettsii*, the agent of Rocky Mountain Spotted Fever [146].

In Australia, three notable *Rickettsia* species are *R. honei*, *R. honei* subsp. *marmionii*, and *R. australis*, which cause Flinders Island Spotted Fever, Australian Spotted Fever, and Queensland Tick Typhus, respectively. Queensland Tick Typhus is vectored by *Ixodes holocyclus*, *I. tasmani*, and *I. cornuatus*, while *B. hydrosauri* is considered a reservoir and probable vector for Flinders Island Spotted Fever [147,148,149]. Australian Spotted Fever was detected in *H. novaeguineae*, but whether this tick species is a vector and/or reservoir host is yet to be determined [150].

Twenty studies have reported the presence of *Rickettsia* species in wildlife hosts and/or their ticks (Table 5). Tick-borne rickettsial diseases generally belong to the spotted-fever group (SFG) of *Rickettsia* [151]. SFG *Rickettsia* were detected in several tick species parasitising wildlife, including *Am. albolimbatum*, *Am. triguttatum*, *Am. moreliae*, *I. tasmani* and *Ar. lagenoplastis* (Table 5).

**Table 5:** *Rickettsia* spp. detected in Australian tick and/or wildlife samples (ticks in parentheses).

Species	Host/Vector/Tissue	References
*Rickettsia australis*	Brush-tailed possum, Black Rat, Bush Rat, Bare-nosed Wombat, Swamp Antechinus (antibodies)	[148]
*Rickettsia honei*	Reptiles (*B. hydrosauri*)	[149]
*Rickettsia* sp. [SFG]	Shingleback skink (*Amblyomma albolimbatum*)	[44]
*Rickettsia* sp.	No host (*B. concolor*, *Haemaphysalis bancrofti*, *H. humerosa*, *Ixodes antechini*, *I. australiensis*, *I. holocyclus*, *I. tasmani*)	[36]
	Brush-tailed possum (*Am. triguttatum*)	[51]
	No Host (*H. bancrofti*)	[31]
*Rickettsia australis*	No Host (*I. holocyclus*)	
*Rickettsia* sp.	Northern brown Bandicoot (*I. tasmani*)	[75]
	Northern Bettong (*Amblyomma* sp.)	
	Northern Bettong (*Am. triguttatum*)	
	Mixed Black Noddy (*Ornithodoros capensis*)	[55]
*Rickettsia* sp. *777c*	Echidna (*B. tachyglossi*)	
*Rickettsia monacensis*		
*Rickettsia* sp. 777b/774e		
*Rickettsia* sp.	‘Bandicoot’ [unspecified] (*H. humerosa*)	
*Rickettsia fournieri* [SFG]	Fairy Martin (*Argas lagenoplastis*)	[56]
*Rickettsia* sp.	Yellow-spotted monitor (*Am. fimbriatum*)	[152]
	Water Python (*Am. fimbriatum*)	
	Green-tree snake (*Am. fimbriatum*)	
*Rickettsia gravesii* [SFG]	Feral Pig (*Am. triguttatum*)	[153]
*Rickettsia bellii*	Echidna (*B. concolor*)	[72]
*Rickettsia massiliae*	Bare-nosed Wombat (*B. auruginans*)	
*Ca.* Rickettsia tasmanensis [SFG]	Tasmanian devil (*I. tasmani*)	[154]
*Rickettsia* sp.	Bare-nosed Wombat (*B. auruginans*), Mountain Brushtail possum (*I. trichosuri*), Southern brown Bandicoot (*I. trichosuri*, *I. tasmani*)	[32]
*Rickettsia* [SFG]	Eastern Blue-tongue lizard (*Am. moreliae*)	[84]
*Rickettsia* spp.	Shingleback Skink (*B. hydrosauri*)	[155]
*Rickettsia* cf. *tamurae*	Lace Monitor (*B. undatum*)	[81]
*Rickettsia* sp.	Unspecified (*I. holocyclus*)	[39]
*Rickettsia* sp. (“*Rickettsia tasmanensis*”) [SFG]	Tasmanian Devil (*I. tasmani*)	[156]
*Rickettsia* (“Koala *Rickettsia*”) [SFG]	Koala (*I. tasmani*)	[157]
*Rickettsia japonica str. argasii*	Gould’s Wattled Bat (*Ar. dewae*), Unidentified Bat sp. (*Ar. dewae*)	[57]

*Ixodes tasmani* was associated with koalas and Tasmanian devils [156,157], whereas *Am. albolimbatum* and *Am. moreliae* were associated with reptile hosts. Furthermore, *B. hydrosauri* has also been implicated with a few different *Rickettsia* species and may serve to be a vector for a greater number of rickettsial diseases in addition to Australian Spotted Fever [155].

Of note, *Am. triguttatum* collected from wild pigs in Western Australia were found to carry *R. gravesii* [153]. Interestingly, *Am. triguttatum* ticks found on horses, in Rockhampton, QLD, were also positive for *R. gravesii* [55]. Given the vast distance between WA and QLD, it may be possible that *R. gravesii* is very widespread within Australia and may be present in other states as well. While the pathogenicity of *R. gravesii* remains unknown, rickettsial-like disease was reported in people in WA that could have been exposed to *Am. triguttatum* infected with *R. gravesii* [158]. These repeated detections suggest a close association between *R. gravesii* and *Am. triguttatum*, although vector competence remains to be demonstrated.

Because many *rickettsiae* are tick endosymbionts, further screening will likely reveal additional species. However, 16S rRNA-based NGS of wildlife ticks [36] and human-biting ticks [131] could not resolve *Rickettsia* to species-level, owing to the conserved nature of this locus [36,129,130]. A *Rickettsia*-specific NGS assay targeting citrate synthase has since improved resolution, enabling the detection of novel species and co-infections within a single host [159]. As a result, any studies to uncover *Rickettsia* may benefit from use of this sequencing technique to delineate *Rickettsia* which may not be possible using other techniques.

The epidemiology of many Australian *Rickettsia*, including endemic species, remains poorly understood [160]. Given the emerging status of rickettsial diseases worldwide, further work should characterise the species detected, assess pathogenic risk to humans and the often-neglected domestic animals, and clarify the vector competence, reservoir status and wildlife maintenance roles underlying their transmission.

#### 3.2.2. Protozoa

Twenty-four studies have identified protozoan species associated with ticks and/or within the blood or tissues of wildlife hosts (Table 6 and Table 7). This section provides an overview of the protozoa with known or suspected public and veterinary health significance in Australia. The genera of greatest relevance are *Babesia* and *Theileria*, both of which include species that are obligate tick-borne haemoparasites. In addition, other potentially important protozoan genera include *Trypanosoma* and *Hepatozoon.*

##### *Babesia*  

The genus *Babesia* consists of tick-borne protists which are obligate intracellular haemoparasites that infect erythrocytes [161]. Members are responsible for causing babesiosis in clinically affected hosts [161]. *Babesia* has significant impacts from a veterinary perspective. *Babesia bigemina* and *Babesia bovis* (along with *A. marginale*) are responsible for causing ‘tick fever’ [bovine babesiosis] in Australian cattle, which are vectored by *Rh. australis*. Tick fever can lead to severe clinical disease due to intraerythrocytic merogony, leading to severe intravascular haemolysis (e.g., associated signs—haemoglobinuria, mortality), fever, abortion. Bovine babesiosis is of extreme importance to the Australian cattle industry due to massive economic costs associated with tick control, loss of production, and the ‘tick-worry’ behaviour of cattle [161]. Dogs are affected by two species—the more pathogenic *Babesia gibsoni* (vectored by *H. longicornis* and *Rh.* (*sanguineus*) *linnaei*) and *Babesia vogeli* (vectored by *Rh.* (*sanguineus*) *linnaei*)—producing anaemia-associated signs (e.g., haemoglobinuria, pallor) that may be worsened by secondary immune-mediated haemolytic anaemia. From a human health perspective, babesiosis is an emerging tick-borne disease. In Australia, this is exemplified by the detection of *Babesia microti* in an individual who ultimately succumbed to infection [162]. Interestingly, the individual did not have any travel history to endemic regions where exposure may have occurred, nor did they have a recent blood transfusion, suggesting that infection was acquired within Australia [162].

There were 10 studies in this review which had reported *Babesia* associated with wildlife tissue and/or their ticks (Table 6). Previous reports of pathogenicity of *Babesia* in wildlife have been seen in experimental studies [163]. In the male brown antechinus, reproduction is associated with a loss of lymphoid tissue, which in turn causes immunosuppression, allowing *Babesia* to cause clinical disease (e.g., anaemia, haemoglobinuria) [163].

**Table 6:** *Babesia* spp. detected in Australian tick and/or wildlife (ticks in parentheses).

Species	Host/Vector/Tissue	References
*Babesia macropus*	Eastern Grey Kangaroo (*Haemaphysalis* sp.)	[164]
	Eastern Grey Kangaroo (tissue, blood), Agile Wallaby (tissue, blood)	[165]
*Babesia* sp.	Northern Brown Bandicoot (blood)	[166]
*Babesia lohae*	Brush-tailed Possum (blood)	[31]
	Questing (*Ixodes trichosuri*)	
*Babesia mackerrasorum*	Questing (*Haemaphysalis humerosa*)	
*Babesia* sp.	Brush-tailed Possum (*I. holocyclus*, *I. tasmani*)	[64]
	Brush-tailed Bettong (blood)	[167]
		[168]
	Brush-tailed Possum (blood)	[125]
	Tasmanian Devil (blood)	[169]
	Little Penguin (blood)	[170]
*Babesia thylacis*	Northern Quoll (blood)	[171]

Pathogenicity in wildlife was seen with *Babesia macropus* in eastern grey kangaroos [164,165] and agile wallabies [165]. Donahoe et al. [165] were able to describe clinical signs of anaemia, lethargy, neurological signs and ultimately death in eastern grey kangaroos and agile wallabies affected by babesiosis. Another study had reported that a caretaker was able to find *Haemaphysalis* species containing *Babesia macropus* but lack of expertise prevented further identification to a species-level [164]. *Babesia macropus* therefore poses a clear threat to eastern grey kangaroos and agile wallabies, and warrants consideration as a differential diagnosis in macropods presenting with anaemia, lethargy or neurological signs. Further investigation of its vectors and the susceptibility of other macropod species would be valuable.

*Babesia lohae* was found in the brush-tailed possum in a blood sample [31]. Additionally, *Babesia mackerrasorum* was found in a *H. humerosa* tick near wildlife habitats [31]. Greay et al. [172], screening ticks from domestic dogs, cats and horses for apicomplexan protozoa, first described both species—*Ba. lohae* in *I. holocyclus* from a cat and *Ba. mackerrasorum* in a cf. *Haemaphysalis* sp. from a horse. The epidemiology for these *Babesia* species remains unknown; it is unclear what the vector species are, and whether the brush-tailed possum is considered a reservoir host for *Babesia lohae*. Neither the *Babesia* species affecting domestic animals nor *Ba. microti* were detected in wildlife or their ticks (Table 6); however, many detected species were novel (Table 6). Characterising these warrants priority, given the possibility that some may infect humans or domestic animals if pathogenic.

##### *Theileria*  

The genus *Theileria*, much like *Babesia*, also consists of tick-borne protists that are obligatory intracellular and haemoparasitic, which leads to the infection of erythrocytes and leukocytes [173]. Members are responsible for causing theileriosis in clinically affected hosts [173]. Benign theileriosis is a serious condition affecting cattle in Australia, as schizogonous stages of replication are responsible for intravascular haemolysis and subsequent anaemia. Clinical disease seen in cattle is attributed to the development of anaemia, with signs being pallor, lethargy, and haematuria, and mortality is possible [173]. The agent responsible for benign theileriosis in Australia is *T. orientalis*, which is vectored by *H. longicornis*.

A total of 12 different studies in this review was able to report *Theileria* species in wildlife tissue or their ticks (Table 7). Much of the detection of *Theileria* was using blood of the selected wildlife hosts (Table 7). *Theileria lupei*, detected across three hosts in the ACT, appears to have a relatively broad host range, possibly reflecting a shared tick vector among infected species [174]. In contrast, *T. penicillata* was found in brush-tailed bettongs (woylie) in three separate studies, with no suggestion of pathogenicity associated with infection [167,174,175]. This suggests that the woylie may be the primary reservoir host. Greay et al. [172] detected *T. paparinii*, *T. apogeana* and *T. worthingtonorum* in *I. tasmani* from dogs. As *I. tasmani* parasitises more mammalian hosts than any other Australian tick, it may also transmit these piroplasms to wildlife, though this requires confirmation [172].

Most of the studies did not report clinical disease due to *Theileria* in the wildlife hosts. However, without blood testing, it is not possible to deduce whether any of the animals were suffering from subclinical anaemia. Only one reviewed study which investigated *Theileria* in the platypus had reported death, which could have been attributed to the high levels of *T. ornithorhynchi* parasitaemia detected [175]. Furthermore, a case of severe disease (e.g., immune-mediated haemolytic anaemia) was reported in a juvenile platypus, which ultimately succumbed due to infection with *T. ornithorhynchi* [176]. The vector is unconfirmed but suspected to be *I. ornithorhynchi*, given its strong host specificity for the platypus; confirming this relationship requires further investigation [177].

Further investigation would be required to confirm the relationship between *I. ornithorhynchi* and *T. ornithorhynchi*. As discussed above, immunosuppression may also be a critical aspect in terms of whether clinical disease due to piroplasmosis occurs in wildlife. All of the platypuses which succumbed were juvenile [175,176]. As a result, the naïve immune system present in younger animals is likely to be a contributing factor.

*Theileria orientalis*, the agent of benign bovine theileriosis, comprises several genotypes, of which Ikeda (and to a lesser extent Chitose and Buffeli) drive disease [173]. Loh et al. [64] detected the DNA of *T. orientalis* in *H. longicornis* tick from a red fox. However, the genotype was not explored [64], and, as a result, the pathogenic potential of the *T. orientalis* found remains unknown for cattle. Further investigations of red foxes and their ticks may prove useful in an attempt to understand the epidemiology of *T. orientalis*. In addition, there was detection of *T.* orientalis genotype Ikeda in *H. longicornis* collected from dogs in NSW [172]. Since the dogs themselves were not directly tested, it is unknown whether *T. orientalis* can be transmitted to dogs, and what their potential role in the epidemiology may be. Additionally, as mentioned above, *H. longicornis* was also found to be present on black rats, swamp rats, bush rats, bare-nosed wombats, and red-necked wallabies; therefore, it may be useful to determine whether any of these species are implicated with *T. orientalis* in any capacity.

**Table 7:** *Theileria* spp. detected in Australian tick and/or wildlife (ticks in parentheses).

Species	Host/Vector/Tissue	References
*Theileria ornithorhynchi*	Platypus (*Ixodes ornithorhynchi*)	[97]
*Theileria* sp.	Platypus (blood)	[178]
*T. ornithorhynchi*		[175]
*Theileria* sp.	Western Grey Kangaroo (*I. australiensis*)	[179]
	Long-nosed Bandicoot (*I. tasmani*)	[64]
*T. orientalis*	Red Fox (*Haemaphysalis longicornis*)	
*T. fuliginosa*	Western Grey Kangaroo (blood)	[180]
*T. penicillata*	Brush-tailed Bettong (blood)	
*T. brachyuri*	Quokka (blood)	[174]
*Theileria* spp.		
*Theileria* sp.	*Theileria* sp. nov	
*T. lupei*	Eastern Quoll (blood)	
	Eastern Bettong (blood)	
	Swamp Wallaby (blood)	
*T.* c.f. *peramelis*	Long-nosed Bandicoot (blood)	[31]
*Theileria* sp. nov	Multiple marsupials (blood)	[174]
*T. penicillata*	Brush-tailed Bettong (blood)	[168]
*T. apogeana*		
*T. penicillata*	Brush-tailed Bettong (blood)	[181]
*T. c.f peramelis*	Black Rat (blood)	[125]
*Theileria* sp.	Brush-tailed Possum (blood)	
	Burrowing Bettong (blood)	[167]
	Eastern Quoll (*I. fecialis*)	[182]
*T. paparinii*	Eastern Quoll (*I. cornuatus* [misidentified as *I. holocyclus*])	
*Theileria* sp.	Koala (*I. tasmani*)	[32]
	Mountain Brush-tailed Possum (*I. trichosuri*)	
	Agile Antechinus (*I. tasmani*, *I. antechini*)	
	Southern Brown Bandicoot (*I. tasmani*)	
*T. gilberti*	Gilbert’s Potoroo (*I. australiensis*, *I. fecialis*, blood)	[183]
*T. worthingtonorum*	Quokka (blood)	[174]
*T. tachyglossus*	Echidna (blood)	[175]

Collectively, studies investigating piroplasms in Australian wildlife and wildlife-associated ticks have revealed substantial genetic diversity, including several apparently endemic and potentially host-adapted lineages. However, the epidemiology, pathogenicity, host specificity, and vector competence of many detected piroplasms remain incompletely understood. Most reviewed studies have relied on the molecular detection of partial genetic loci, and comparatively few investigations have integrated phylogenetics, ecology, and clinical significance. Continued application of genomic approaches, expanded wildlife surveillance, and vector competence studies will be important for clarifying the evolutionary relationships and epidemiological significance of Australian piroplasms.

### 3.3. Other Tick-Borne Microorganisms (Trypanosoma, Hepatozoon, Arboviruses)

The genus *Trypanosoma* consists of flagellated protists that disseminate through blood and infiltrate various body organs once present within a host [184]. *Trypanosoma* is responsible for causing various diseases in different parts of the world. For example, the African continent is associated with severe trypanosomiasis. Transmission is mediated with dipteran vectors, notably tsetse flies responsible for transmitting *T. brucei gambiense*/*T. brucei rhodesiense* causing sleeping sickness in humans and *T. brucei brucei* causing Nagana in a number of domestic animals [184].

A total of nine studies had reported *Trypanosoma* species that were attributed to ticks affecting wildlife, e.g. [31,67,69,125,168,169,178,185] (Supplementary Table S2). Many of the studies were able to demonstrate the presence of *Trypanosoma* species within ticks. However, no study has demonstrated vector competence. Austen et al. [185] suggested that *I. australiensis* was responsible for the transmission of *T. copemani* through tick faeces. However, it was counter-argued that tick faeces are not suitable for the survivability of *Trypanosoma*; therefore, suggesting their methodology was not appropriate [186].

Tick-borne transmission of trypanosomes in Australia remains unconfirmed [186]. Given that trypanosomiasis has been implicated in clinical disease in an Australian flying fox [187], further work should clarify the pathogenic and zoonotic significance of these parasites and, critically, establish whether Australian ticks are competent vectors—which would be unusual relative to *Trypanosoma* transmission elsewhere.

The genus *Hepatozoon* consists of apicomplexan tick-borne protists which infect the blood and tissues of hosts [188,189]. The transmission of *Hepatozoon* species occurs via tick ingestion rather than typical inoculation through blood-feeding [189]. Clinical disease is referred to as hepatozoonosis and can be seen in domestic dogs and occasionally cats [189]. While infections in dogs and cats are most commonly subclinical or mild, severe signs that can be seen if clinical disease occurs include anaemia, immunosuppression, hepatitis, glomerulonephritis, and pneumonia depending on levels of parasitaemia and/or the presence of co-infection [188,189]. Transplacental transmission can also occur, leading to infection in new-born puppies [189,190].

Several agents of canine and feline hepatozoonosis exist, but the primary agent responsible for hepatozoonosis in dogs worldwide is *Hepatozoon canis* (*Hep. canis*) [189]. *Hepatozoon canis* is also capable of causing infection in cats [189]. While *Hep. canis* was previously deemed exotic to Australia, *I. holocyclus* collected from a dog was found to be infected with *Hep. canis* [172,188].

A total of five studies has found *Hepatozoon* species associated in wildlife and tick samples [31,46,125,166,182] (Supplementary Table S2). The vector potential of ticks found to be infected is unknown, and the ability to cause clinical disease in wildlife was unexplored. In other parts of the world, *Hepatozoon* species in general do not result in clinical disease in wildlife [191]. However, severe clinical disease has been reported in wild canids [191]. The significance of these wildlife *Hepatozoon* species for domestic animals is unexplored, and none of the studies detected *Hep. canis*; further surveillance should establish its status in Australia. *Hepatozoon canis* may prove to be a significant threat to wild canids (e.g., foxes, dingoes) and risk subsequent spillover events to domestic dogs and cats [188]. Additionally, it is also important that *I. holocyclus* be explored as a possible vector for *Hep. canis*.

Globally, tick-borne arboviruses cause severe human diseases (e.g., Crimean–Congo haemorrhagic fever, tick-borne encephalitis, Powassan virus disease) and veterinary diseases (e.g., Nairobi sheep disease, African swine fever), which are all currently exotic to Australia [192].

Ten studies in this review had reported various viruses within ticks associated with wildlife [31,61,62,86,111,112,193,194,195,196] (Supplementary Table S2). NGS techniques have proven very helpful in the detection of viruses within tick samples [31,86]. A majority of the viruses found was previously undiscovered and were named after the area in which they were found (e.g., Collins Beach virus) [31,86]. Many of these viruses have unknown pathogenicity and transmissibility, with the exception of reports of seroconversion of similar tick arboviruses found in seabirds [193]. *Ixodes holocyclus* was found to have the greatest number of novel viruses [31,86,195,196]. Other ticks implicated with viruses were *H. bancrofti* [31], *Ar. robertsi* [61,62], *Am. moreliae* [86], *I. trichosuri* [86,182,196], *I. uriae* [194], and *I. eudyptidis* [111,112]. For most viruses detected in Australian ticks, vector competence and transmission dynamics remain unresolved. Nevertheless, the recognised capacity of tick-borne arboviruses to cause severe disease internationally highlights the importance of continued surveillance and characterisation of tick-associated viruses in Australia.

Compared with bacterial and protozoan microorganisms, viruses associated with Australian wildlife ticks remain comparatively under-investigated. Most available studies have relied on molecular screening approaches or opportunistic detection, with limited information regarding viral pathogenicity, host range, transmission dynamics, or zoonotic potential. Advances in next-generation sequencing and metagenomic approaches have substantially expanded the discovery of novel viral sequences in ticks; however, the biological and epidemiological significance of many detected viruses remains unclear. Furthermore, few studies have experimentally assessed vector competence or reservoir host status in Australian wildlife systems. Given increasing recognition of the ecological and public health significance of wildlife-associated arboviruses and emerging zoonotic threats globally, further investigations integrating genomics, virus isolation, vector competence studies, and wildlife surveillance are required to better understand the diversity and public health relevance of tick-associated viruses in Australia.

### 3.4. Identification Methods of Tick-Borne Microorganisms

Tick-borne microorganisms in Australia have been identified predominantly by molecular methods (*n* = 60/69; Supplementary Table S2), typically PCR (conventional, nested or real-time) followed by sequencing (Sanger or NGS), though some studies combined approaches—for example, NGS followed by PCR and further sequencing. In addition, there were some studies which elected to use NGS exclusively for all [50,86] or select samples [51,197]. In particular, NGS has proven extremely useful for wildlife sampling, allowing for very extensive detection of several different species of bacteria, protozoa, and viruses associated with ticks [31,36,39,50,51,54,67,81,86,111,175,195,196]. Regardless of technique, many of these molecular techniques have enabled us to detect pathogens which have never been discovered before. Ultimately, this proves there is considerable diversity present within Australian ticks, and there are likely many more discoveries as research continues. Additional advantages from the use of molecular techniques include the exploration of potential host ranges, detection of endosymbionts, and identification of pathogens which may look very similar (e.g., *Babesia*, *Theileria*) [159].

In addition to the method of detection, several other factors may influence the tick-borne microorganisms that are detected and should be considered when aiming to identify tick-borne microorganisms. It was demonstrated the DNA extraction technique can play a role in influencing the abundance and diversity of bacterial species within ticks [81]. Additionally, Gofton et al. identified a prominent endosymbiont of *I. holocyclus* being *Ca.* Midichloria mitochondrii and developed a blocking primer against this endosymbiont [39]. Despite use of the primer, the endosymbiont was still the predominant bacterium detected [39]. However, the use of the blocking primer enabled additional bacterial species to be uncovered [39]. Further studies aiming to investigate additional TBPs should be cognizant of their DNA extraction technique, including any available blocking primers for endosymbionts to enhance detection. Additionally, further investigation of other endosymbionts, and the development of primers against those species may prove useful as well.

Detection within ticks is complicated by blood-meal effects: engorged ticks carry host blood and skin at collection, making it difficult to determine whether a detected organism originates from the tick or the host. To address this, some authors sampled ticks alongside host blood and tissue to test for concurrent detection [51,125]. It was partially successful, as they confirmed the concurrent infection of *Bartonella* sp., *Ca.* Neoehrlichia arcana, *Ca.* Neoehrlichia australis within *Ixodes* ticks from black rats [51] as other microorganisms were only detected either in tick or host samples independently (Supplementary Table S2).

Research in the northern hemisphere has demonstrated the ability of host blood-meal influences on the tick microbiome [198,199]. In particular, it was found that *Ixodes pacificus* ticks feeding on western fence lizards showed significantly reduced diversity in the tick microbiome and later reduced the capacity of acquisition and transmission of Lyme disease [198]. Additional research has shown that host blood meal may play a role in *Ixodes scapularis* microbiomes, and that individual differences may also exist [199]. No studies in this review attempted to compare the relative differences in microbiomes for any of the tick species feeding on multiple hosts. Future studies should be conducted to explore this possibility in the Australian context, especially regarding host generalist ticks (e.g., *I. holocyclus*, *I. tasmani*) as different hosts may serve an important role for influencing tick microbiomes and ultimately altering the potential pathogen transmission of ticks. This would require identifying prior host blood meals—difficult given that ticks feed once per life stage with long intervals between feeds. Blood-meal identification techniques used elsewhere (PCR, blotting, high-resolution melt analysis, mass spectrometry, retrotransposon-based PCR) [200] have not been applied in the Australian studies reviewed here. Overall, as greater understanding of tick microbiomes advances, it will allow us to understand what potential pathogens may exist within various tick species.

### 3.5. Genetic Diversity and Phylogeography of Wildlife Ticks and Tick-Borne Microorganisms

Molecular tools have substantially enhanced our understanding of Australian wildlife ticks and their associated tick-borne microorganisms, yet genetic and phylogeographic data remain fragmented and geographically biassed. Most genetic studies to date have used mitochondrial and ribosomal markers such as COI, 16S rRNA, 12S rRNA and ITS regions to support species identification, explore cryptic diversity and infer relationships among Australian ticks and tick-borne microorganisms [32,42,51,80,84,85]. More recently, NGS approaches, including amplicon-based metabarcoding and untargeted metagenomics, have begun to reveal the broader microbiomes of ticks and the diversity of microorganisms they harbour [31,54,86].

For ticks, COI and 16S rRNA sequences have confirmed the validity of several described species and helped clarify relationships within genera such as *Ixodes*, *Haemaphysalis*, *Bothriocroton* and *Amblyomma* [2,5]. Molecular data have also highlighted cases where morphology alone is insufficient, particularly for immature stages and morphologically similar species. For example, genetic analyses of *Ixodes* spp. associated with small mammals have identified putative cryptic species that are difficult or impossible to distinguish morphologically [85], indicating that tick diversity in Australian wildlife is underestimated. Similar approaches have supported the recent description or redescription of several taxa (e.g., *I. barkeri*, *I. woyliei*, *I. heathi*, and a novel *Cryptocroton* species) and have begun to delineate their host ranges and ecological niches [42,47,48,52,89]. However, comprehensive phylogeographic studies remain rare; for most tick species, there are only scattered COI haplotypes rather than dense sampling suitable for reconstructing population structure, dispersal routes or historical range shifts.

Genetic data for tick-borne bacteria are in some respects more advanced than for the ticks themselves, particularly for *Anaplasma*, *Borrelia*, *Coxiella* and *Rickettsia* [31,36,39,50]. Housekeeping genes (16S rRNA, *groEL*, *gltA*) have shown that many lineages in wildlife ticks—including novel *Anaplasma* genotypes, multiple *Borrelia* lineages, *Coxiella*-like endosymbionts and spotted-fever-group *Rickettsia*—are genetically distinct from recognised human and livestock pathogens, often forming novel clades or ‘*Candidatus*’ species [31,36,39,44,50,52,54,56,72,132,137,156]. These findings imply a long co-evolutionary history among ticks, endosymbionts and wildlife hosts, though the phylogeography and host specificity of most lineages remain poorly understood.

The use of NGS-based metabarcoding and metagenomics has further expanded the catalogue of bacterial and viral diversity in Australian wildlife ticks [31,86]. High-throughput sequencing of 16S rRNA amplicons has shown that the tick microbiome is dominated by endosymbionts in some species, such as *Candidatus* Midichloria mitochondrii in *Ixodes holocyclus* [39], and that blocking primers may be required to uncover less abundant but epidemiologically relevant bacteria. Metagenomic sequencing of tick homogenates has revealed numerous novel RNA viruses, many of which are currently only known from sequence data and whose pathogenic potential is unknown [31,195,196]. These genomic approaches provide a powerful way to survey wildlife ticks for emergent pathogens but are often limited to small numbers of sites or tick species; broad-scale comparative datasets across biomes and host communities are still lacking.

For protozoa, genetic studies have focused mainly on piroplasms (*Babesia*, *Theileria*) and, less so, *Trypanosoma* and *Hepatozoon* [31,125,164,167,174,175]. Piroplasm studies have used 18S rRNA, sometimes with additional loci, to describe novel species, infer host specificity (e.g., *T. ornithorhynchi* in platypus, *T. penicillata* in woylies) and explore links to livestock pathogens such as *T. orientalis* [64,172]. Detection of *T. orientalis* genotypes, including Ikeda, in *Haemaphysalis longicornis* removed from wildlife or companion animals highlights the potential for wildlife–domestic animal interfaces to influence the epidemiology of endemic livestock diseases. However, few studies have combined detailed host, tick and pathogen genotyping within the same system, so the direction and frequency of cross-species transmission remain unclear.

Genetic data for *Trypanosoma* and *Hepatozoon* spp. in Australian wildlife are also emerging, mainly from 18S rRNA sequencing of blood or tissue samples and occasionally from ticks [31,125,166,182,185]. These analyses have revealed multiple distinct lineages, some clustering with known pathogenic species and others representing novel wildlife-associated clades. Yet, the vectors and transmission cycles of these lineages are often unresolved; in particular, the role of ticks in transmitting trypanosomes in Australia remains controversial [186]. More integrative genetic studies that couple parasite genotyping with tick and host sampling are required to clarify whether these lineages pose a genuine spillover risk to domestic animals or humans.

Overall, the available genetic data point to the substantial—and probably still underestimated—diversity of both ticks and tick-borne microorganisms in Australian wildlife. However, most studies have been opportunistic, geographically restricted and reliant on single-locus markers. Future work would benefit from (i) coordinated sampling across underrepresented regions and host taxa; (ii) the use of multi-locus genotyping, whole-mitochondrial genomes or genomic approaches for key tick species; (iii) integrated host–tick–pathogen genotyping to resolve transmission networks; and (iv) explicit phylogeographic analyses to test hypotheses about climate-driven range shifts, host movements and the emergence of new wildlife–livestock–human interfaces [32,51,125,201]. Embedding such genetic and genomic studies within long-term ecological and epidemiological frameworks will be essential to understand how wildlife ticks and their pathogens respond to environmental change and to anticipate emerging infectious disease risks.

## 4. Conclusions and Future Implications

Much remains to be discovered about Australian wildlife ticks, as the detection of hybrids and newly described species attests. Identification will increasingly depend on integrating updated morphological keys, molecular tools and emerging approaches such as the matrix-assisted laser desorption ionisation–time of flight mass spectrometry (MALDI-TOF MS), which has proven useful elsewhere [91,202].

Tick host ranges are also expected to shift with climate change. Models project an initial expansion followed by contraction in the distribution of *Ixodes holocyclus* [201], although their accuracy is limited by weather unpredictability and the omission of host movement. As sampling has been concentrated in eastern and south-eastern Australia, routine tick and host surveillance across underrepresented regions may reveal unrecognised sylvatic cycles.

As human encroachment on wildlife habitats increases, so does the risk of pathogen spillover. Although many sampled wildlife appeared clinically healthy—possibly through co-evolution and acquired immunity—and none of the novel organisms detected to date have confirmed pathogenicity, their potential to affect immunologically naïve humans or domestic animals warrants continued surveillance and characterisation.

A critical gap in most pathogen-focused studies was the failure to establish tick–pathogen relationships and vector competence; several did not identify the tick species or localities involved, and none determined true vector potential. In some instances, tick species have been described as ‘vectors’ on the basis of detection alone, without experimental transmission evidence (e.g., [148,185]); such terminology should be applied cautiously. Tick vector competence should be determined through experimental trials. Trials must demonstrate acquisition in immature life stages, replication and/or persistence, where applicable, trans-stadial maintenance, and the transmission of the pathogen to a susceptible host [203]. During these trials, it may also be possible to determine the feeding time or attachment duration required to complete these individual steps. While the risk of transmission generally increases with longer feeding time, different pathogens can be transmitted at different times [204,205]. Collectively, the additional studies identified through this review broaden the evidence base for Australian wildlife–tick associations and tick-associated microorganisms across diverse host taxa and ecosystems [206,207,208,209,210,211,212,213,214,215,216,217,218,219,220,221,222,223,224,225,226,227,228,229,230,231,232,233]. Together, these records reinforce the ecological complexity of wildlife-associated tick systems in Australia and highlight the continuing need to clarify transmission dynamics, vector competence and disease significance.

Tick ecology in Australia also remains poorly understood. In the well-studied Northern Hemisphere Lyme disease system, the white-footed mouse acts as an amplification host, transmitting *Bo. burgdorferi* sensu lato efficiently to larvae, whereas deer support tick reproduction but rarely transmit the bacterium and therefore act as dilution hosts. In Australia, similar roles have been hypothesised for small and large mammals, but this has not been formally tested [51]. Because Australian tick and wildlife fauna are distinct, Northern Hemisphere patterns cannot be assumed to apply directly. Clarifying which hosts drive pathogen transmission and tick maintenance would allow amplification and dilution hosts to be identified, supporting strategic disease prevention. This is particularly important for generalist species such as *I. holocyclus* and *I. tasmani*, which parasitise multiple wildlife hosts and are associated with several detected microorganisms.

Because tick taxonomy, molecular diagnostics and pathogen discovery are evolving rapidly, knowledge in this field continues to expand; nonetheless, the evidence collated here provides a current synthesis to guide future research and surveillance. Several limitations of the available literature should be acknowledged. Much of the evidence reviewed was derived from opportunistic or cross-sectional sampling, often with substantial variation in host species examined, geographic coverage, sample sizes and diagnostic methodologies. Many earlier studies relied exclusively on morphological identification, while molecular investigations frequently targeted short genetic loci with variable taxonomic resolution. In addition, relatively few studies incorporated longitudinal sampling, experimental vector competence assessments or concurrent host health evaluations, limiting the interpretation of the ecological and epidemiological significance of detected microorganisms. Geographic bias toward eastern and south-eastern Australia was also evident throughout the literature. Consequently, direct comparisons among studies should be interpreted cautiously, and current understanding of wildlife-associated ticks and microorganisms in Australia remains incomplete.

Consistent with its design as a scoping evidence synthesis, this review did not include a formal assessment of methodological quality or risk of bias for the included studies. The findings should therefore be read as a map of the available evidence rather than a quality-weighted appraisal. A further challenge in interpreting this literature is its methodological heterogeneity over time: earlier studies relied largely on microscopy and serology, whereas more recent work has applied molecular and metagenomic approaches. The resulting differences in diagnostic sensitivity and specificity confound temporal and geographical comparisons and may influence apparent patterns of microbial presence and diversity.

Interpreted through a One Health lens, these patterns reflect more than sampling artefact. Habitat modification, urban expansion and climate change are reshaping where wildlife, domestic animals and people overlap, and with them the distribution of ticks and the microorganisms they carry. The wildlife–domestic animal–human interface is therefore central to anticipating tick-borne disease risk in Australia, and identifying which associations represent genuine spillover potential will require surveillance that spans these sectors rather than treating One Health as a framing concept alone.

Overall, this review highlights the considerable diversity of ticks and tick-borne microorganisms associated with Australian wildlife alongside substantial ecological, epidemiological and molecular knowledge gaps. Evidence is biassed toward particular hosts, regions and pathogen groups, and for many detected organisms, vector competence, reservoir status, pathogenicity and transmission dynamics remain unresolved. While advances in genomics and metagenomics are improving characterisation, integrating ecological, veterinary, wildlife and public health research within a One Health framework—through standardised, geographically expanded and longitudinal surveillance—is required to clarify their significance.

## Data Availability

The original contributions presented in this study are included in the article/Supplementary Material. Further inquiries can be directed to the corresponding authors.

## Supplementary Materials

The following supporting information can be downloaded at https://www.mdpi.com/article/10.3390/pathogens15060646/s1, Table S1. Wildlife hosts and their associated tick species, as reported in the reviewed studies. Each entry includes tick species (with life stages where reported), study location(s), identification method(s), and tick occurrence data (where available).; Table S2. Tick-borne microorganism studies in Australian wildlife, organised by host species and sample type.; File S1. PRISMA-ScR checklist [234].

## References

[1] Barker S.C., Barker D. (2018). Ticks in Australia: Endemics; exotics; which ticks bite humans?. Microbiol. Aust..

[2] Barker S.C., Barker D. (2023). Ticks of Australasia: 125 species of ticks in and around Australia. Zootaxa.

[3] Hall-Mendelin S., Craig S.B., Hall R.A., O’Donoghue P., Atwell R.B., Tulsiani S.M., Graham G.C. (2011). Tick paralysis in Australia caused by *Ixodes holocyclus* Neumann. Ann. Trop. Med. Parasitol..

[4] Barker S.C., Walker A.R. (2014). Ticks of Australia: The species that infest domestic animals and humans. Zootaxa.

[5] Barker S.C., Walker A.R., Campelo D. (2014). A list of the 70 species of Australian ticks; diagnostic guides to and species accounts of *Ixodes holocyclus* (paralysis tick), *Ixodes cornuatus* (southern paralysis tick) and *Rhipicephalus australis* (Australian cattle tick); and consideration of the place of Australia in the evolution of ticks with comments on four controversial ideas. Int. J. Parasitol..

[6] Dehhaghi M., Kazemi Shariat Panahi H., Holmes E.C., Hudson B.J., Schloeffel R., Guillemin G.J. (2019). Human tick-borne diseases in Australia. Front. Cell. Infect. Microbiol..

[7] Anderson J.F., Magnarelli L.A. (2008). Biology of ticks. Infect. Dis. Clin. N. Am..

[8] Estrada-Peña A. (2015). Ticks as vectors: Taxonomy, biology and ecology. Rev. Sci. Tech..

[9] Estrada-Peña A., Venzal J.M., Kocan K.M., Sonenshine D.E. (2008). Overview: Ticks as vectors of pathogens that cause disease in humans and animals. Front. Biosci..

[10] Jongejan F., Uilenberg G. (2004). The global importance of ticks. Parasitology.

[11] Gilbert L. (2021). The impacts of climate change on ticks and tick-borne disease risk. Annu. Rev. Entomol..

[12] Tsao J.I., Hamer S.A., Han S., Sidge J.L., Hickling G.J. (2021). The contribution of wildlife hosts to the rise of ticks and tick-borne diseases in North America. J. Med. Entomol..

[13] Chapman A.D. (2009). Numbers of Living Species in Australia and the World.

[14] Cresswell I., Murphy H. (2017). Australia State of the Environment 2016: Biodiversity.

[15] Dielenberg J., Bekessy S., Cumming G.S., Dean A.J., Fitzsimons J.A., Garnett S., Goolmeer T., Hughes L., Kingsford R.T., Legge S. (2023). Australia’s biodiversity crisis and the need for the Biodiversity Council. Ecol. Manag. Restor..

[16] Taylor-Brown A., Booth R., Gillett A., Mealy E., Ogbourne S.M., Polkinghorne A., Conroy G.C. (2019). The impact of human activities on Australian wildlife. PLoS ONE.

[17] Scanes C.G., Scanes C.G., Toukhsati S.R. (2018). Human activity and habitat loss: Destruction, fragmentation, and degradation. Animals and Human Society.

[18] Driga A.M., Drigas A.S. (2019). Climate change 101: How everyday activities contribute to the ever-growing issue. Int. J. Recent Contrib. Eng. Sci. IT.

[19] Onyango M.G., Ciota A.T., Kramer L.D. (2020). The vector-host-pathogen interface: The next frontier in the battle against mosquito-borne viral diseases?. Front. Cell. Infect. Microbiol..

[20] Stephen C., Duncan C. (2022). Climate Change and Animal Health.

[21] Jones K.E., Patel N.G., Levy M.A., Storeygard A., Balk D., Gittleman J.L., Daszak P. (2008). Global trends in emerging infectious diseases. Nature.

[22] McArthur D.B. (2019). Emerging infectious diseases. Nurs. Clin. N. Am..

[23] Oskam C., Ronai I., Irwin P. (2021). The emergence of tick-borne diseases in domestic animals in Australia. Climate, Ticks and Disease.

[24] Schnall J., Oliver G., Braat S., Macdonell R., Gibney K.B., Kanaan R.A. (2021). Characterising DSCATT: A case series of Australian patients with debilitating symptom complexes attributed to ticks. Aust. N. Z. J. Psychiatry.

[25] Irwin P.J., Robertson I.D., Westman M.E., Perkins M., Straubinger R.K. (2017). Searching for Lyme borreliosis in Australia: Results of a canine sentinel study. Parasites Vectors.

[26] Jackson S., Groves C. (2015). Taxonomy of Australian Mammals.

[27] Baker A.M., Gynther I. (2023). Strahan’s Mammals of Australia.

[28] Bush A.O., Lafferty K.D., Lotz J.M., Shostak A.W. (1997). Parasitology meets ecology on its own terms: Margolis et al. revisited. J. Parasitol..

[29] Fenner A.L., Bull C.M. (2007). *Bothriocroton hydrosauri* (formerly *Aponomma hydrosauri*) (Denny, 1843) (Acari: Ixodidae), new parasite record for the endangered pygmy bluetongue lizard, *Tiliqua adelaidensis* (Scincidae) from Australia. Comp. Parasitol..

[30] Vilcins I.M., Old J.M., Koertner G., Deane E.M. (2008). Ectoparasites and skin lesions in wild-caught spotted-tailed quoll (*Dasyurus maculatus*) (Marsupialia: Dasyuridae). Comp. Parasitol..

[31] Gofton A.W., Blasdell K.R., Taylor C., Banks P.B., Michie M., Roy-Dufresne E., Poldy J., Wang J., Dunn M., Tachedjian M. (2022). Metatranscriptomic profiling reveals diverse tick-borne bacteria, protozoans and viruses in ticks and wildlife from Australia. Transbound. Emerg. Dis..

[32] Ghafar A., Davies N., Tadepalli M., Breidahl A., Death C., Haros P., Li Y., Dann P., Cabezas-Cruz A., Moutailler S. (2023). Unravelling the diversity of microorganisms in ticks from Australian wildlife. Pathogens.

[33] Taylor C.L.L., Egan S.L.L., Gofton A.W., Irwin P.J., Oskam C.L.L., Hochuli D.F., Banks P.B. (2023). An invasive human commensal and a native marsupial maintain tick populations at the urban fringe. Med. Vet. Entomol..

[34] Burnard D., Weaver H., Gillett A., Loader J., Flanagan C., Polkinghorne A. (2017). Novel *Chlamydiales* genotypes identified in ticks from Australian wildlife. Parasites Vectors.

[35] Hillman A.E., Lymbery A.J., Elliot A.D., Ash A.L., Thompson R.C.A. (2018). Parasitic infections of brushtail possums *Trichosurus vulpecula* in urbanised environments and bushland in the greater Perth region, Western Australia. Wildl. Biol..

[36] Egan S.L., Loh S.M., Banks P.B., Gillett A., Ahlstrom L., Ryan U.M., Irwin P.J., Oskam C.L. (2020). Bacterial community profiling highlights complex diversity and novel organisms in wildlife ticks. Ticks Tick-Borne Dis..

[37] Taylor C.L., Lydecker H.W., Lo N., Hochuli D.F., Banks P.B. (2020). Invasive rabbits host immature *Ixodes* ticks at the urban-forest interface. Ticks Tick-Borne Dis..

[38] Hill A.G., Clark N.J., Tokonami F. (2021). Tick paralysis in Australian birds caused by *Ixodes holocyclus*. Aust. Vet. J..

[39] Gofton A.W., Oskam C.L., Lo N., Beninati T., Wei H., McCarl V., Murray D.C., Paparini A., Greay T.L., Holmes A.J. (2015). Inhibition of the endosymbiont “*Candidatus* Midichloria mitochondrii” during 16S rRNA gene profiling reveals potential pathogens in *Ixodes* ticks from Australia. Parasites Vectors.

[40] Krige A.S., Loh S.M., Oskam C.L. (2017). New host records for ticks (Acari: Ixodidae) from the echidna (*Tachyglossus aculeatus*) revealed in Australian museum survey. Aust. J. Zool..

[41] Barker D., Kelava S., Shao R., Seeman O.D., Jones M.K., Nakao R., Barker S.C., Apanaskevich D.A. (2022). Description of the female, nymph and larva and mitochondrial genome, and redescription of the male of *Ixodes barkeri* Barker, 2019 (Acari: Ixodidae), from the short-beaked echidna, *Tachyglossus aculeatus*, with a consideration of the most suitable subgenus for this tick. Parasites Vectors.

[42] Ash A., Elliot A., Godfrey S., Burmej H., Abdad M.Y., Northover A., Wayne A., Morris K., Clode P., Lymbery A. (2017). Morphological and molecular description of *Ixodes woyliei* n. sp. (Ixodidae) with consideration for co-extinction with its critically endangered marsupial host. Parasites Vectors.

[43] Payne E., Sinn D.L., Spiegel O., Leu S.T., Wohlfeil C., Godfrey S.S., Gardner M., Sih A. (2020). Consistent individual differences in ecto-parasitism of a long-lived lizard host. Oikos.

[44] Tadepalli M., Vincent G., Hii S.F., Watharow S., Graves S., Stenos J. (2021). Molecular evidence of novel spotted fever group *Rickettsia* species in *Amblyomma albolimbatum* ticks from the shingleback skink (*Tiliqua rugosa*) in southern Western Australia. Pathogens.

[45] Norval G., Sharrad R.D., Gardner M.G. (2022). A mammal tick with a taste for lizard blood: Parasitism by the kangaroo soft tick, *Ornithodoros gurneyi* on sleepy lizards (*Tiliqua rugosa*). Ticks Tick-Borne Dis..

[46] Vilcins I.M.E., Ujvari B., Old J.M., Deane E. (2009). Molecular and morphological description of a *Hepatozoon* species in reptiles and their ticks in the Northern Territory, Australia. J. Parasitol..

[47] Weaver H.J. (2016). Redescription of *Ixodes victoriensis* Nuttall, 1916 (Ixodida: Ixodidae) from marsupials in Victoria, Australia. Syst. Appl. Acarol..

[48] Heath A.C.G., Palma R.L. (2017). A new species of tick (Acari: Ixodidae) from seabirds in New Zealand and Australia, previously misidentified as *Ixodes eudyptidis*. Zootaxa.

[49] Kwak M.L., Madden C., Wicker L. (2018). *Ixodes heathi* n. sp. (Acari: Ixodidae), a co-endangered tick from the critically endangered mountain pygmy possum (*Burramys parvus*), with notes on its biology and conservation. Exp. Appl. Acarol..

[50] Beard D., Stannard H.J., Old J.M. (2021). Morphological identification of ticks and molecular detection of tick-borne pathogens from bare-nosed wombats (*Vombatus ursinus*). Parasites Vectors.

[51] Egan S.L., Taylor C.L., Banks P.B., Northover A.S., Ahlstrom L.A., Ryan U.M., Irwin P.J., Oskam C.L. (2021). The bacterial biome of ticks and their wildlife hosts at the urban-wildland interface. Microb. Genom..

[52] Barker D., Kelava S., Seeman O.D., Shao R., Seaniger J.R., Jones M.K., Apanaskevich M.A., Nakao R., Apanaskevich D.A., Barker S.C. (2022). Rediscovery of *Ixodes confusus* in Australia with the first description of the male from Australia, a redescription of the female and the mitochondrial (mt) genomes of five species of *Ixodes*. Int. J. Parasitol. Parasites Wildl..

[53] Jackson J., Beveridge I., Chilton N.B., Andrews R.H. (2007). Distributions of the paralysis ticks *Ixodes cornuatus* and *Ixodes holocyclus* in south-eastern Australia. Aust. Vet. J..

[54] Gofton A.W., Popa-Baez A., Takano A., Soennichsen K., Michie M., Short M., Supriyono S., Pascoe J., Cusbert S., Mulley R. (2023). Characterisation and comparative genomics of three new *Varanus*-associated *Borrelia* spp. from Indonesia and Australia. Parasites Vectors.

[55] Chalada M.J., Stenos J., Vincent G., Barker D., Bradbury R.S. (2018). A molecular survey of tick-borne pathogens from ticks collected in Central Queensland, Australia. Vector Borne Zoonotic Dis..

[56] Diop A., Barker S.C., Eberhard M., Barker D., Thi Tien N., Di Pinto F., Raoult D., Mediannikov O. (2018). *Rickettsia fournieri* sp. nov., a novel spotted fever group rickettsia from *Argas lagenoplastis* ticks in Australia. Int. J. Syst. Evol. Microbiol..

[57] Izzard L., Chung M., Dunning Hotopp J.C., Vincent G., Paris D., Graves S., Stenos J. (2018). Isolation of a divergent strain of *Rickettsia japonica* from Dew’s Australian bat argasid ticks (*Argas (Carios) dewae*) in Victoria, Australia. Ticks Tick-Borne Dis..

[58] Keirans J.E., Bull C.M., Duffield G.A. (1996). *Amblyomma vikirri* n. sp. (Acari: Ixodida: Ixodidae), a parasite of the gidgee skink *Egernia stokesii* (Reptilia: Scincidae) from South Australia. Syst. Parasitol..

[59] Waudby H.P., Petit S., Dixon B., Andrews R.H. (2007). Hosts of the exotic ornate kangaroo tick, *Amblyomma triguttatum triguttatum* Koch, on southern Yorke Peninsula, South Australia. Parasitol. Res..

[60] Bennett M.D., Woolford L., Banazis M.J., O’Hara A.J., Warren K.S., Nicholls P.K., Sims C., Fenwick S.G. (2011). *Coxiella burnetii* in western barred bandicoots (*Perameles bougainville*) from Bernier and Dorre Islands in Western Australia. Ecohealth.

[61] Hoogstraal H., Kaiser M.N., McClure H.E. (1974). Subgenus *Persicargas* (Ixodoidea: Argasidae: *Argas*). 20. *A. (P.) robertsi* parasitizing nesting wading birds and domestic chickens in Australian and Oriental regions, viral infections, and host migration. J. Med. Entomol..

[62] St George T.D., Cybinski D.H., Main A.J., McKilligan N., Kemp D.H. (1984). Isolation of a new arbovirus from the tick *Argas robertsi* from a cattle egret (*Bubulcus ibis coromandus*) colony in Australia. Aust. J. Biol. Sci..

[63] Oorebeek M., Kleindorfer S. (2009). The prevalence and intensity of tick infestation in passerines from South Australia. Emu.

[64] Loh S.M., Egan S., Gillett A., Banks P.B., Ryan U.M., Irwin P.J., Oskam C.L. (2018). Molecular surveillance of piroplasms in ticks from small and medium-sized urban and peri-urban mammals in Australia. Int. J. Parasitol. Parasites Wildl..

[65] Andrews R.H., Petney T.N. (2008). The distribution of reptile ticks in South Australia: More complex than assumed. Syst. Appl. Acarol..

[66] Roberts F.H.S. (1970). Australian Ticks.

[67] Barbosa A.D., Gofton A.W., Paparini A., Codello A., Greay T., Gillett A., Warren K., Irwin P., Ryan U. (2017). Increased genetic diversity and prevalence of co-infection with *Trypanosoma* spp. in koalas (*Phascolarctos cinereus*) and their ticks identified using next-generation sequencing (NGS). PLoS ONE.

[68] Lydecker H.W., Hochuli D.F., Banks P.B. (2019). Peri-urban black rats host a rich assembly of ticks and healthier rats have more ticks. Ticks Tick-Borne Dis..

[69] Krige A.S., Thompson R.C.A., Seidlitz A., Keatley S., Wayne J., Clode P.L. (2021). Molecular detection of *Trypanosoma* spp. in questing and feeding ticks (*Ixodidae*) collected from an endemic region of south-west Australia. Pathogens.

[70] Brown B., Copeman D.B. (2003). Zoonotic importance of parasites in wild dogs caught in the vicinity of Townsville. Aust. Vet. J..

[71] Andrews R.H., Beveridge I., Bull C.M., Chilton N.B., Dixon B., Petney T. (2006). Systematic status of *Aponomma tachyglossi* Roberts (Acari: *Ixodidae*) from echidnas, *Tachyglossus aculeatus*, from Queensland, Australia. Syst. Appl. Acarol..

[72] Vilcins I.M.E., Old J.M., Deane E. (2009). Molecular detection of *Rickettsia*, *Coxiella* and *Rickettsiella* DNA in three native Australian tick species. Exp. Appl. Acarol..

[73] Storey-Lewis B., Mitrovic A., McParland B. (2018). Molecular detection and characterisation of *Babesia* and *Theileria* in Australian hard ticks. Ticks Tick-Borne Dis..

[74] Waudby H.P., Petit S., Matthews B., Sharp A., Pradhan R., Dale B. (2019). Investigation of ticks and red blood cell parasites of a population of reintroduced mainland tammar wallabies (*Notamacropus eugenii eugenii*). Aust. Mammal..

[75] Hussain-Yusuf H., Stenos J., Vincent G., Shima A., Abell S., Preece N.D., Tadepalli M., Hii S.F., Bowie N., Mitram K. (2020). Screening for *Rickettsia*, *Coxiella* and *Borrelia* species in ticks from Queensland, Australia. Pathogens.

[76] Barnden B., Slender A.L., Sharrad R.D., Gardner M.G. (2022). Changes in parasite species distributions could be driven by host range expansions: The case of hybridisation between two Australian reptile ticks. Aust. J. Zool..

[77] Steventon C., Harley D., Wicker L., Legione A.R., Devlin J.M., Hufschmid J. (2022). An assessment of ectoparasites across highland and lowland populations of Leadbeater’s possum (*Gymnobelideus leadbeateri*): Implications for genetic rescue translocations. Int. J. Parasitol. Parasites Wildl..

[78] Kleindorfer S., Lambert S., Paton D.C. (2006). Ticks (*Ixodes* sp.) and blood parasites (*Haemoproteus* spp.) in New Holland honeyeaters (*Phylidonyris novaehollandiae*): Evidence for site specificity and fitness costs. Emu.

[79] Chapman T.W., Marando L., Oorebeek M., Kleindorfer S. (2009). Genetic structure in ixodid ticks from Kangaroo Island and the South Australia mainland. Aust. J. Entomol..

[80] Hammer J.F., Emery D., Bogema D.R., Jenkins C. (2015). Detection of *Theileria orientalis* genotypes in *Haemaphysalis longicornis* ticks from southern Australia. Parasites Vectors.

[81] Panetta J.L., Šíma R., Calvani N.E.D., Hajdušek O., Chandra S., Panuccio J., Šlapeta J. (2017). Reptile-associated *Borrelia* species in the goanna tick (*Bothriocroton undatum*) from Sydney, Australia. Parasites Vectors.

[82] Taggart P.L., Schultz D. (2017). Do avian ticks (*Ixodes hirsti*) influence host phenotype in New Holland honeyeaters (*Phylidonyris novaehollandiae*)?. Trans. R. Soc. S. Aust..

[83] Moon K.L., Chown S.L., Fraser C.I. (2019). Tandem host-parasite dispersal inferred from similarities in phylogeographical patterns among little penguins and their ‘terrestrial’ ectoparasites. J. Biogeogr..

[84] Kim M.M., Shea G., Šlapeta J. (2024). Detection of tick-borne bacterial DNA (*Rickettsia* sp.) in reptile ticks *Amblyomma moreliae* from New South Wales, Australia. Parasitol. Res..

[85] McCann K.M., Grant W.N., Spratt D.M., Hedtke S.M. (2019). Cryptic species diversity in ticks that transmit disease in Australia. Int. J. Parasitol. Parasites Wildl..

[86] Harvey E., Rose K., Eden J.S., Lo N., Abeyasuriya T., Shi M., Doggett S.L., Holmes E.C. (2019). Extensive diversity of RNA viruses in Australian ticks. J. Virol..

[87] Abouelhassan E.M., El-Gawady H.M., Abdel-Aal A.A., El-Gayar A.K., Esteve-Gassent M.D. (2019). Comparison of some molecular markers for tick species identification. J. Arthropod Borne Dis..

[88] Korshunova T., Picton B., Furfaro G., Mariottini P., Pontes M., Prkić J., Fletcher K., Malmberg K., Lundin K., Martynov A. (2019). Multilevel fine-scale diversity challenges the ‘cryptic species’ concept. Sci. Rep..

[89] Barker S.C., Kelava S., Mans B.J., Apanaskevich D.A., Seeman O.D., Gofton A.W., Shao R., Teo E.J.M., Evasco K.L., Soennichsen K.F. (2024). The first cryptic genus of Ixodida, *Cryptocroton* n. gen. for *Amblyomma papuanum* Hirst, 1914: A tick of North Queensland, Australia, and Papua New Guinea. Zootaxa.

[90] Léger E., Liu X., Masseglia S., Noël V., Vourc’h G., Bonnet S., McCoy K.D. (2015). Reliability of molecular host-identification methods for ticks: An experimental in vitro study with *Ixodes ricinus*. Parasites Vectors.

[91] Galletti M.F.B.M., Hecht J.A., McQuiston J.R., Gartin J., Cochran J., Blocher B.H., Ayres B.N., Allerdice M.E.J., Beati L., Nicholson W.L. (2024). Applying MALDI-TOF MS to resolve morphologic and genetic similarities between two *Dermacentor* tick species of public health importance. Sci. Rep..

[92] Andrews R.H., Chilton N.B. (1999). Multilocus enzyme electrophoresis: A valuable technique for providing answers to problems in parasite systematics. Int. J. Parasitol..

[93] Jackson J., Chilton N.B., Beveridge I., Morris M., Andrews R.H. (2000). Genetic variation within the ticks *Ixodes holocyclus* and *Ixodes cornuatus* from south-eastern Australia. Int. J. Parasitol..

[94] Andrews R.H., Petney T.N. (2007). Variation in the attachment sites of ticks to Australian lizards. Syst. Appl. Acarol..

[95] Lydecker H.W., Etheridge B., Price C., Banks P.B., Hochuli D.F. (2019). Landscapes within landscapes: A parasite utilizes different ecological niches on the host landscapes of two host species. Acta Trop..

[96] McKenzie R.A. (1981). Observations on diseases of free-living and captive koalas (*Phascolarctos cinereus*). Aust. Vet. J..

[97] Whittington R.J., Spratt D.M. (1989). Lesions associated with metazoan parasites of wild platypuses (*Ornithorhynchus anatinus*). J. Wildl. Dis..

[98] Campbell F.E., Atwell R.B., Smart L. (2003). Effects of the paralysis tick, *Ixodes holocyclus*, on the electrocardiogram of the spectacled flying fox, *Pteropus conspicillatus*. Aust. Vet. J..

[99] Barnes T.S., Goldizen A.W., Morton J.M., Coleman G.T. (2010). Parasites of the brush-tailed rock-wallaby (*Petrogale penicillata*). J. Wildl. Dis..

[100] Buettner P.G., Westcott D.A., Maclean J., Brown L., McKeown A., Johnson A., Wilson K., Blair D., Luly J., Skerratt L. (2013). Tick paralysis in spectacled flying-foxes (*Pteropus conspicillatus*) in North Queensland, Australia: Impact of a ground-dwelling ectoparasite finding an arboreal host. PLoS ONE.

[101] Gemmell R.T., Cepon G., Green P.E., Stewart N.P. (1991). Some effects of tick infestations on juvenile northern brown bandicoot (*Isoodon macrourus*). J. Wildl. Dis..

[102] Spencer A.J., Canfield P.J. (1993). Haematological characterisation of heavy tick infestation in koalas (*Phascolarctos cinereus*). Comp. Haematol. Int..

[103] Vogelnest L., Woods R. (2009). Medicine of Australian Mammals.

[104] Kitsou C., Fikrig E., Pal U. (2021). Tick host immunity: Vector immunomodulation and acquired tick resistance. Trends Immunol..

[105] Pfäffle M., Littwin N., Muders S.V., Petney T.N. (2013). The ecology of tick-borne diseases. Int. J. Parasitol..

[106] Chilton N.B., Andrews R.H., Bull C.M. (2000). Influence of temperature and relative humidity on the moulting success of *Amblyomma limbatum* and *Aponomma hydrosauri* (Acari: Ixodidae) larvae and nymphs. Int. J. Parasitol..

[107] Turni C., Smales L.R. (2001). Parasites of the bridled nailtail wallaby (*Onychogalea fraenata*) (Marsupialia: Macropodidae). Wildl. Res..

[108] Lorch D., Fisher D.O., Spratt D.M. (2007). Variation in ectoparasite infestation on the brown antechinus, *Antechinus stuartii*, with regard to host, habitat and environmental parameters. Aust. J. Zool..

[109] Waudby H.P., Petit S. (2007). Seasonal density fluctuations of the exotic ornate kangaroo tick, *Amblyomma triguttatum triguttatum* Koch, and its distribution on Yorke Peninsula, South Australia. Parasitol. Res..

[110] Holz P.H., Lumsden L.F., Hufschmid J. (2018). Ectoparasites are unlikely to be a primary cause of population declines of bent-winged bats in south-eastern Australia. Int. J. Parasitol. Parasites Wildl..

[111] Wang J., Selleck P., Yu M., Ha W., Rootes C., Gales R., Wise T., Crameri S., Chen H., Broz I. (2014). Novel phlebovirus with zoonotic potential isolated from ticks, Australia. Emerg. Infect. Dis..

[112] Gauci P.J., McAllister J., Mitchell I.R., St George T.D., Cybinski D.H., Davis S.S., Gubala A.J. (2015). Hunter Island Group phlebovirus in ticks, Australia. Emerg. Infect. Dis..

[113] Taggart P.L., Traub R., Fui S., Weinstein P. (2018). Attempt to uncover reservoirs of human spotted fever rickettsiosis on the Fleurieu Peninsula, South Australia. J. Vector Borne Dis..

[114] Kada S., McCoy K.D., Boulinier T. (2017). Impact of life stage-dependent dispersal on the colonization dynamics of host patches by ticks and tick-borne infectious agents. Parasites Vectors.

[115] Lydecker H., Stanfield E., Lo N., Hochuli D., Banks P. (2015). Are urban bandicoots solely to blame for tick concerns?. Aust. Zool..

[116] Kwak M.L. (2018). The first records of human infestation by the hard tick *Ixodes (Endopalpiger) australiensis* (Acari: Ixodidae), with a review of human infestation by ticks in Australia. Exp. Appl. Acarol..

[117] Norval G., Sharrad R.D., Gardner M.G. (2020). Three instances of reptile ticks parasitising humans. Acarologia.

[118] Egan S.L., Lettoof D.C., Oskam C.L. (2022). First record of the stump-tailed lizard tick, *Amblyomma albolimbatum* (Ixodida, Ixodidae) parasitising a human. Ticks Tick-Borne Dis..

[119] Commins S.P. (2020). Diagnosis & management of alpha-gal syndrome: Lessons from 2,500 patients. Expert Rev. Clin. Immunol..

[120] Vlahov D., Galea S. (2002). Urbanization, urbanicity, and health. J. Urban Health.

[121] Bradley C.A., Altizer S. (2007). Urbanization and the ecology of wildlife diseases. Trends Ecol. Evol..

[122] Becker D.J., Hall R.J., Forbes K.M., Plowright R.K., Altizer S. (2018). Anthropogenic resource subsidies and host–parasite dynamics in wildlife. Philos. Trans. R. Soc. Lond. B Biol. Sci..

[123] Webster K.N., Hill N.J., Burnett L., Deane E.M. (2014). Ectoparasite infestation patterns, haematology and serum biochemistry of urban-dwelling common brushtail possums. Wildl. Biol..

[124] Hillman A.E., Lymbery A.J., Elliot A.D., Thompson R.C.A. (2017). Urban environments alter parasite fauna, weight and reproductive activity in the quenda (*Isoodon obesulus*). Sci. Total Environ..

[125] Egan S.L., Taylor C.L., Austen J.M., Banks P.B., Northover A.S., Ahlstrom L.A., Ryan U.M., Irwin P.J., Oskam C.L. (2021). Haemoprotozoan surveillance in peri-urban native and introduced wildlife from Australia. Curr. Res. Parasitol. Vector Borne Dis..

[126] Battilani M., De Arcangeli S., Balboni A., Dondi F. (2017). Genetic diversity and molecular epidemiology of *Anaplasma*. Infect. Genet. Evol..

[127] Jonsson N.N., Bock R.E., Jorgensen W.K. (2008). Productivity and health effects of anaplasmosis and babesiosis on *Bos indicus* cattle and their crosses, and the effects of differing intensity of tick control in Australia. Vet. Parasitol..

[128] Brown G.K., Martin A.R., Roberts T.K., Aitken R.J. (2001). Detection of *Ehrlichia platys* in dogs in Australia. Aust. Vet. J..

[129] Shapiro A.J., Brown G., Norris J.M., Bosward K.L., Marriot D.J., Balakrishnan N., Breitschwerdt E.B., Malik R. (2017). Vector-borne and zoonotic diseases of dogs in north-west New South Wales and the Northern Territory, Australia. BMC Vet. Res..

[130] Arraga-Alvarado C.M., Qurollo B.A., Parra O.C., Berrueta M.A., Hegarty B.C., Breitschwerdt E.B. (2014). Case report: Molecular evidence of *Anaplasma platys* infection in two women from Venezuela. Am. J. Trop. Med. Hyg..

[131] Gofton A.W., Doggett S., Ratchford A., Oskam C.L., Paparini A., Ryan U., Irwin P. (2015). Bacterial profiling reveals novel “*Ca. Neoehrlichia*”, *Ehrlichia*, and *Anaplasma* species in Australian human-biting ticks. PLoS ONE.

[132] Gofton A.W., Waudby H.P., Petit S., Greay T.L., Ryan U.M., Irwin P.J. (2017). Detection and phylogenetic characterisation of novel *Anaplasma* and *Ehrlichia* species in *Amblyomma triguttatum* subsp. from four allopatric populations in Australia. Ticks Tick-Borne Dis..

[133] Rar V., Golovljova I. (2011). *Anaplasma*, *Ehrlichia*, and “*Candidatus* Neoehrlichia” bacteria: Pathogenicity, biodiversity, and molecular genetic characteristics, a review. Infect. Genet. Evol..

[134] Seo M.G., Ouh I.O., Kwak D. (2023). Detection and genotypic analysis of *Anaplasma bovis* and *A. phagocytophilum* in horse blood and lung tissue. Int. J. Mol. Sci..

[135] Karpathy S.E., Kingry L., Pritt B.S., Berry J.C., Chilton N.B., Dergousoff S.J., Cortinas R., Sheldon S.W., Oatman S., Anacker M. (2023). *Anaplasma bovis*-like infections in humans, United States, 2015–2017. Emerg. Infect. Dis..

[136] Chalada M.J., Stenos J., Bradbury R.S. (2016). Is there a Lyme-like disease in Australia? Summary of the findings to date. One Health.

[137] Loh S.M., Gofton A.W., Lo N., Gillett A., Ryan U.M., Irwin P.J., Oskam C.L. (2016). Novel *Borrelia* species detected in echidna ticks, *Bothriocroton concolor*, in Australia. Parasites Vectors.

[138] Tang T., Zhu Y., Zhang Y.Y., Chen J.J., Tian J.B., Xu Q., Jiang B.G., Wang G.L., Golding N., Mehlman M.L. (2024). The global distribution and the risk prediction of relapsing fever group *Borrelia*: A data review with modelling analysis. Lancet Microbe.

[139] Carley J., Pope J. (1962). A new species of *Borrelia* (*B. queenslandica*) from *Rattus villosissimus* in Queensland. Aust. J. Exp. Biol. Med. Sci..

[140] Celina S.S., Černý J. (2022). *Coxiella burnetii* in ticks, livestock, pets and wildlife: A mini-review. Front. Vet. Sci..

[141] Maurin M., Raoult D. (1999). Q fever. Clin. Microbiol. Rev..

[142] Cooper A., Stephens J., Ketheesan N., Govan B. (2013). Detection of *Coxiella burnetii* DNA in wildlife and ticks in Northern Queensland, Australia. Vector Borne Zoonotic Dis..

[143] Graves S.R., Jackson C., Hussain-Yusuf H., Vincent G., Nguyen C., Stenos J., Webster M. (2016). *Ixodes holocyclus* tick-transmitted human pathogens in north-eastern New South Wales, Australia. Trop. Med. Infect. Dis..

[144] Duron O., Noël V., McCoy K.D., Bonazzi M., Sidi-Boumedine K., Morel O., Vavre F., Zenner L., Jourdain E., Durand P. (2015). The recent evolution of a maternally-inherited endosymbiont of ticks led to the emergence of the Q fever pathogen, *Coxiella burnetii*. PLoS Pathog..

[145] Duron O., Sidi-Boumedine K., Rousset E., Moutailler S., Jourdain E. (2015). The importance of ticks in Q fever transmission: What has (and has not) been demonstrated?. Trends Parasitol..

[146] Fang R., Blanton L.S., Walker D.H. (2017). Rickettsiae as emerging infectious agents. Clin. Lab. Med..

[147] Sexton D.J., Dwyer B., Kemp R., Graves S. (1991). Spotted fever group rickettsial infections in Australia. Rev. Infect. Dis..

[148] Graves S.R., Stewart L., Stenos J., Stewart R.S., Schmidt E., Hudson S., Banks J., Huang Z.H., Dwyer B. (1993). Spotted fever group rickettsial infection in south-eastern Australia: Isolation of rickettsiae. Comp. Immunol. Microbiol. Infect. Dis..

[149] Stenos J., Graves S., Popov V.L., Walker D.H. (2003). *Aponomma hydrosauri*, the reptile-associated tick reservoir of *Rickettsia honei* on Flinders Island, Australia. Am. J. Trop. Med. Hyg..

[150] Unsworth N.B., Stenos J., Graves S.R., Faa A.G., Cox G.E., Dyer J.R., Boutlis C.S., Lane A.M., Shaw M.D., Robson J. (2007). Flinders Island spotted fever rickettsioses caused by “marmionii” strain of *Rickettsia honei*, Eastern Australia. Emerg. Infect. Dis..

[151] Tomassone L., Portillo A., Nováková M., de Sousa R., Oteo J.A. (2018). Neglected aspects of tick-borne rickettsioses. Parasites Vectors.

[152] Vilcins I.M., Fournier P.E., Old J.M., Deane E. (2009). Evidence for the presence of *Francisella* and spotted fever group *Rickettsia* DNA in the tick *Amblyomma fimbriatum* (Acari: Ixodidae), Northern Territory, Australia. J. Med. Entomol..

[153] Li A.Y., Adams P.J., Abdad M.Y., Fenwick S.G. (2010). High prevalence of *Rickettsia gravesii* sp. nov. in *Amblyomma triguttatum* collected from feral pigs. Vet. Microbiol..

[154] Izzard L., Graves S., Cox E., Fenwick S., Unsworth N., Stenos J. (2009). Novel *Rickettsia* in ticks, Tasmania, Australia. Emerg. Infect. Dis..

[155] Whiley H., Custance G., Graves S., Stenos J., Taylor M., Ross K., Gardner M.G. (2016). *Rickettsia* detected in the reptile tick *Bothriocroton hydrosauri* from the lizard *Tiliqua rugosa* in South Australia. Pathogens.

[156] Vilcins I.M., Old J.M., Deane E. (2009). Detection of a *Hepatozoon* and spotted fever group *Rickettsia* species in the common marsupial tick (*Ixodes tasmani*) collected from wild Tasmanian devils (*Sarcophilus harrisii*), Tasmania. Vet. Parasitol..

[157] Vilcins I.M., Old J.M., Deane E.M. (2008). Detection of a spotted fever group *Rickettsia* in the tick *Ixodes tasmani* collected from koalas in Port Macquarie, Australia. J. Med. Entomol..

[158] Abdad M.Y., Abdallah R.A., Karkouri K.E., Beye M., Stenos J., Owen H., Unsworth N., Robertson I., Blacksell S.D., Nguyen T.T. (2017). *Rickettsia gravesii* sp. nov.: A novel spotted fever group rickettsia in Western Australian *Amblyomma triguttatum triguttatum* ticks. Int. J. Syst. Evol. Microbiol..

[159] Greay T.L., Evasco K.L., Evans M.L., Oskam C.L., Magni P.A., Ryan U.M., Irwin P.J. (2021). Illuminating the bacterial microbiome of Australian ticks with 16S and *Rickettsia*-specific next-generation sequencing. Curr. Res. Parasitol. Vector Borne Dis..

[160] Stewart A., Armstrong M., Graves S., Hajkowicz K. (2017). *Rickettsia australis* and Queensland tick typhus: A rickettsial spotted fever group infection in Australia. Am. J. Trop. Med. Hyg..

[161] Bock R., Jackson L., De Vos A., Jorgensen W. (2004). Babesiosis of cattle. Parasitology.

[162] Senanayake S.N., Paparini A., Latimer M., Andriolo K., Dasilva A.J., Wilson H., Xayavong M.V., Collignon P.J., Jeans P., Irwin P.J. (2012). First report of human babesiosis in Australia. Med. J. Aust..

[163] Barker I., Beveridge I., Bradley A., Lee A.K. (1978). Observations on spontaneous stress-related mortality among males of the dasyurid marsupial *Antechinus stuartii* Macleay. Aust. J. Zool..

[164] Dawood K.E., Morgan J.A., Busfield F., Srivastava M., Fletcher T.I., Sambono J., Jackson L.A., Venus B., Philbey A.W., Lew-Tabor A.E. (2013). Observation of a novel *Babesia* spp. in eastern grey kangaroos (*Macropus giganteus*) in Australia. Int. J. Parasitol. Parasites Wildl..

[165] Donahoe S.L., Peacock C.S., Choo A.Y.L., Cook R.W., O’Donoghue P., Crameri S., Vogelnest L., Gordon A.N., Scott J.L., Rose K. (2015). A retrospective study of *Babesia macropus* associated with morbidity and mortality in eastern grey kangaroos (*Macropus giganteus*) and agile wallabies (*Macropus agilis*). Int. J. Parasitol. Parasites Wildl..

[166] Barbosa A., Reiss A., Jackson B., Warren K., Paparini A., Gillespie G., Stokeld D., Irwin P., Ryan U. (2017). Prevalence, genetic diversity and potential clinical impact of blood-borne and enteric protozoan parasites in native mammals from northern Australia. Vet. Parasitol..

[167] Paparini A., Ryan U.M., Warren K., McInnes L.M., de Tores P., Irwin P.J. (2012). Identification of novel *Babesia* and *Theileria* genotypes in the endangered marsupials, the woylie (*Bettongia penicillata ogilbyi*) and boodie (*Bettongia lesueur*). Exp. Parasitol..

[168] Northover A.S., Godfrey S.S., Keatley S., Lymbery A.J., Wayne A.F., Cooper C., Pallant L., Morris K., Thompson R.C.A. (2019). Increased *Trypanosoma* spp. richness and prevalence of haemoparasite co-infection following translocation. Parasites Vectors.

[169] Egan S.L., Ruiz-Aravena M., Austen J.M., Barton X., Comte S., Hamilton D.G., Hamede R.K., Ryan U.M., Irwin P.J., Jones M.E. (2020). Blood parasites in endangered wildlife—Trypanosomes discovered during a survey of haemoprotozoa from the Tasmanian devil. Pathogens.

[170] Vanstreels R.E., Woehler E.J., Ruoppolo V., Vertigan P., Carlile N., Priddel D., Finger A., Dann P., Herrin K.V., Thompson P. (2015). Epidemiology and molecular phylogeny of *Babesia* sp. in little penguins *Eudyptula minor* in Australia. Int. J. Parasitol. Parasites Wildl..

[171] Bangs M.J. (1996). *Babesia thylacis* (Apicomplexa: Babesiidae) in a northern quoll, *Dasyurus hallucatus* (Marsupialia: Dasyuridae), from Western Australia. J. Helminthol. Soc. Wash..

[172] Greay T.L., Zahedi A., Krige A.S., Owens J.M., Rees R.L., Ryan U.M., Oskam C.L., Irwin P.J. (2018). Endemic, exotic and novel apicomplexan parasites detected during a national study of ticks from companion animals in Australia. Parasites Vectors.

[173] Jenkins C. (2018). Bovine theileriosis in Australia: A decade of disease. Microbiol. Aust..

[174] Barbosa A.D., Austen J., Portas T.J., Friend J.A., Ahlstrom L.A., Oskam C.L., Ryan U.M., Irwin P.J. (2019). Sequence analyses at mitochondrial and nuclear loci reveal a novel *Theileria* sp. and aid in the phylogenetic resolution of piroplasms from Australian marsupials and ticks. PLoS ONE.

[175] Šlapeta J., Saverimuttu S., Vogelnest L., Sangster C., Hulst F., Rose K., Thompson P., Whittington R. (2017). Deep-sequencing to resolve complex diversity of apicomplexan parasites in platypuses and echidnas: Proof of principle for wildlife disease investigation. Infect. Genet. Evol..

[176] Kessell A.E., Boulton J.G., Dutton G.J., Woodgate R., Shamsi S., Peters A., Connolly J.H. (2014). Haemolytic anaemia associated with *Theileria* sp. in an orphaned platypus. Aust. Vet. J..

[177] Kwak M.L., Griffiths J., Barry D., Begent M., Hoang T., Taafua L., Chiovitti A. (2018). The first DNA barcodes for the Australian platypus tick *Ixodes ornithorhynchi* Lucas, 1846 (Acari: Ixodidae) to facilitate conservation efforts for a declining parasite and its host. Acarologia.

[178] Macgregor J.W., Holyoake C.S., Munks S.A., Connolly J.H., Robertson I.D., Fleming P.A., Warren K.S. (2017). Investigation into individual health and exposure to infectious agents of platypuses (*Ornithorhynchus anatinus*) in two river catchments in northwest Tasmania. J. Wildl. Dis..

[179] Loh S.M., Paparini A., Ryan U., Irwin P., Oskam C. (2018). Identification of *Theileria fuliginosa*-like species in *Ixodes australiensis* ticks from western grey kangaroos (*Macropus fuliginosus*) in Western Australia. Ticks Tick-Borne Dis..

[180] Clark P., Spencer P.B.S. (2007). Description of three new species of *Theileria* Bettencourt, Franca & Borges, 1907 from Macropodoidea in Western Australia. Trans. R. Soc. S. Aust..

[181] Rong J., Bunce M., Wayne A., Pacioni C., Ryan U., Irwin P. (2012). A high prevalence of *Theileria penicillata* in woylies (*Bettongia penicillata*). Exp. Parasitol..

[182] Portas T.J., Evans M.J., Spratt D., Vaz P.K., Devlin J.M., Barbosa A.D., Wilson B.A., Rypalski A., Wimpenny C., Fletcher D. (2020). Baseline health and disease assessment of founder eastern quolls (*Dasyurus viverrinus*) during a conservation translocation to mainland Australia. J. Wildl. Dis..

[183] Lee J.Y., Ryan U.M., Jefferies R., McInnes L.M., Forshaw D., Friend J.A., Irwin P.J. (2009). *Theileria gilberti* n. sp. (Apicomplexa: Theileriidae) in the Gilbert’s potoroo (*Potorous gilbertii*). J. Eukaryot. Microbiol..

[184] Büscher P., Cecchi G., Jamonneau V., Priotto G. (2017). Human African trypanosomiasis. Lancet.

[185] Austen J.M., Ryan U.M., Friend J.A., Ditcham W.G.F., Reid S.A. (2011). Vector of *Trypanosoma copemani* identified as *Ixodes* sp.. Parasitology.

[186] Krige A.S., Thompson R.C.A., Clode P.L. (2019). ‘Hang on a tick’—Are ticks really the vectors for Australian trypanosomes?. Trends Parasitol..

[187] Mackie J.T., Stenner R., Gillett A.K., Barbosa A., Ryan U., Irwin P.J. (2017). Trypanosomiasis in an Australian little red flying fox (*Pteropus scapulatus*). Aust. Vet. J..

[188] Greay T.L., Barbosa A.D., Rees R.L., Paparini A., Ryan U.M., Oskam C.L., Irwin P.J. (2018). An Australian dog diagnosed with an exotic tick-borne infection: Should Australia still be considered free from *Hepatozoon canis*?. Int. J. Parasitol..

[189] Baneth G., Allen K. (2022). Hepatozoonosis of dogs and cats. Vet. Clin. N. Am. Small Anim. Pract..

[190] Schäfer I., Müller E., Nijhof A.M., Aupperle-Lellbach H., Loesenbeck G., Cramer S., Naucke T.J. (2022). First evidence of vertical *Hepatozoon canis* transmission in dogs in Europe. Parasites Vectors.

[191] Uiterwijk M., Vojta L., Šprem N., Beck A., Jurković D., Kik M., Duscher G.G., Hodžić A., Reljić S., Sprong H. (2023). Diversity of *Hepatozoon* species in wild mammals and ticks in Europe. Parasites Vectors.

[192] Labuda M., Nuttall P.A. (2004). Tick-borne viruses. Parasitology.

[193] Humpherysmith I., Cybinski D.H., Byrnes K.A., St George T.D. (1991). Seroepidemiology of arboviruses among seabirds and island residents of the Great Barrier Reef and Coral Sea. Epidemiol. Infect..

[194] Major L., La Linn M., Slade R.W., Schroder W.A., Hyatt A.D., Gardner J., Cowley J., Suhrbier A. (2009). Ticks associated with Macquarie Island penguins carry arboviruses from four genera. PLoS ONE.

[195] O’Brien C.A., Hall-Mendelin S., Hobson-Peters J., Deliyannis G., Allen A., Lew-Tabor A., Rodriguez-Valle M., Barker D., Barker S.C., Hall R.A. (2018). Discovery of a novel iflavirus sequence in the eastern paralysis tick *Ixodes holocyclus*. Arch. Virol..

[196] Porter A.F., Pettersson J.H., Chang W.S., Harvey E., Rose K., Shi M., Eden J.S., Buchmann J., Moritz C., Holmes E.C. (2020). Novel hepaci- and pegi-like viruses in native Australian wildlife and non-human primates. Virus Evol..

[197] Gofton A.W., Loh S.M., Barbosa A.D., Paparini A., Gillett A., Macgregor J., Oskam C.L., Ryan U.M., Irwin P.J. (2018). A novel *Ehrlichia* species in blood and *Ixodes ornithorhynchi* ticks from platypuses (*Ornithorhynchus anatinus*) in Queensland and Tasmania, Australia. Ticks Tick-Borne Dis..

[198] Swei A., Kwan J.Y. (2017). Tick microbiome and pathogen acquisition altered by host blood meal. ISME J..

[199] Landesman W.J., Mulder K., Allan B.F., Bashor L.A., Keesing F., LoGiudice K., Ostfeld R.S. (2019). Potential effects of blood meal host on bacterial community composition in *Ixodes scapularis* nymphs. Ticks Tick-Borne Dis..

[200] Goethert H.K., Mather T.N., Buchthal J., Telford S.R. (2021). Retrotransposon-based blood meal analysis of nymphal deer ticks demonstrates spatiotemporal diversity of *Borrelia burgdorferi* and *Babesia microti* reservoirs. Appl. Environ. Microbiol..

[201] Teo E.J.M., Vial M.N., Hailu S., Kelava S., Zalucki M.P., Furlong M.J., Barker D., Barker S.C. (2021). Climatic requirements of the eastern paralysis tick, *Ixodes holocyclus*, with a consideration of its possible geographic range up to 2090. Int. J. Parasitol..

[202] Ngnindji-Youdje Y., Diarra A.Z., Lontsi-Demano M., Berenger J.M., Tchuinkam T., Parola P. (2023). MALDI-TOF MS identification of cattle ticks from Cameroon. Ticks Tick-Borne Dis..

[203] Eisen L. (2020). Vector competence studies with hard ticks and *Borrelia burgdorferi sensu lato* spirochetes: A review. Ticks Tick-Borne Dis..

[204] Richards S.L., Langley R., Apperson C.S., Watson E. (2017). Do tick attachment times vary between different tick-pathogen systems?. Environments.

[205] Eisen L. (2018). Pathogen transmission in relation to duration of attachment by *Ixodes scapularis* ticks. Ticks Tick-Borne Dis..

[206] McOrist S., Smales L. (1986). Morbidity and mortality of free-living and captive echidnas, *Tachyglossus aculeatus* (Shaw), in Australia. J. Wildl. Dis..

[207] Laan B., Handasyde K., Beveridge I. (2011). Occurrence of the tick *Haemaphysalis bancrofti* Nuttall & Warburton, 1915 in Victoria with additional data on its distribution and with scanning electron micrographs of life cycle stages. Proc. R. Soc. Vic..

[208] Vilcins I.M., Kosoy M., Old J.M., Deane E.M. (2009). *Bartonella*-like DNA detected in *Ixodes tasmani* ticks (Acari: Ixodida) infesting koalas (*Phascolarctos cinereus*) in Victoria, Australia. Vector Borne Zoonotic Dis..

[209] Laan B., Handasyde K., Beveridge I. (2011). Observations on the biology and distribution of the tick *Ixodes hirsti* Hassall, 1931 (Acari: Ixodoidea). Proc. R. Soc. Vic..

[210] Hall J., Rose K., Austen J., Egan S., Bilney R., Kambouris P., MacGregor C., Dexter N. (2021). Baseline health parameters for a newly established population of long-nosed potoroo (*Potorous tridactylus*) at Booderee National Park, Australia. J. Wildl. Dis..

[211] Kaewmongkol G., Kaewmongkol S., Burmej H., Bennett M.D., Fleming P.A., Adams P.J., Wayne A.F., Ryan U., Irwin P.J., Fenwick S.G. (2011). Diversity of *Bartonella* species detected in arthropod vectors from animals in Australia. Comp. Immunol. Microbiol. Infect. Dis..

[212] Portas T., Fletcher D., Spratt D., Reiss A., Holz P., Stalder K., Devlin J., Taylor D., Dobroszczyk D., Manning A.D. (2014). Health evaluation of free-ranging eastern bettongs (*Bettongia gaimardi*) during translocation for reintroduction in Australia. J. Wildl. Dis..

[213] Tozer S.J., Lambert S.B., Strong C.L., Field H.E., Sloots T.P., Nissen M.D. (2014). Potential animal and environmental sources of Q fever infection for humans in Queensland. Zoonoses Public Health.

[214] Jackson J. (1998). A new host record for *Ixodes holocyclus* Neumann and *Ixodes cordifer* Neumann (Acarina: Ixodidae) in Australia. Aust. J. Entomol..

[215] Weaver H.J. (2014). New host records for ticks (Ixodidae) from the northern quoll (*Dasyurus hallucatus*) in north Queensland. Aust. J. Zool..

[216] Oakwood M., Spratt D.M. (2000). Parasites of the northern quoll, *Dasyurus hallucatus* (Marsupialia: Dasyuridae) in tropical savanna, Northern Territory. Aust. J. Zool..

[217] Kaewmongkol G., Kaewmongkol S., Owen H., Fleming P.A., Adams P.J., Ryan U., Irwin P.J., Fenwick S.G. (2011). *Candidatus Bartonella antechini*: A novel *Bartonella* species detected in fleas and ticks from the yellow-footed antechinus (*Antechinus flavipes*), an Australian marsupial. Vet. Microbiol..

[218] Chakma S., Picard J., Duffy R., Constantinoiu C., Gummow B. (2017). A survey of zoonotic pathogens carried by non-indigenous rodents at the interface of the Wet Tropics of North Queensland, Australia. Transbound. Emerg. Dis..

[219] Smales L.R., Miller A.K., Obendorf D.L. (1990). Parasites of the water rat, *Hydromys chrysogaster*, from Victoria and South Australia. Aust. J. Zool..

[220] Mulder E., Smales L.R. (2009). Parasites of *Rattus colletti* (Rodentia: Muridae) from the Adelaide River floodplain, Northern Territory, and comparison with assemblages in other *Rattus* species. Aust. J. Zool..

[221] Heise-Pavlov P.M., Heise-Pavlov S.R. (2003). Feral pigs in tropical lowland rainforest of northeastern Australia: Ecology, zoonoses and management. Wildl. Biol..

[222] Fenner A.L., Godfrey S.S., Bull C.M. (2011). Using social networks to deduce whether residents or dispersers spread parasites in a lizard population. J. Anim. Ecol..

[223] Petney T.N., Dixon B.R., Andrews R.H. (2008). A new host and disquieting distribution record for *Amblyomma triguttatum triguttatum*. Syst. Appl. Acarol..

[224] Duffield G.A., Bull C.M. (1996). Microhabitat choice and its role in determining the distribution of the reptile tick *Amblyomma vikirri*. Aust. J. Ecol..

[225] Smallridge C.J., Bull C.M. (2001). Prevalence of infection by the protozoan *Hemolivia mariae* in ticks. Parasitol. Res..

[226] Bull C.M., Burzacott D. (1993). The impact of tick load on the fitness of their lizard hosts. Oecologia.

[227] Guzinski J., Bull C.M., Donnellan S.C., Gardner M.G. (2009). Molecular genetic data provide support for a model of transmission dynamics in an Australian reptile tick, *Bothriocroton hydrosauri*. Mol. Ecol..

[228] Tucker A.D. (1995). First record of parasitism by a tick on an Australian freshwater crocodile. Mem. Qld. Mus..

[229] Kwak M.L., Madden C. (2017). The first record of infestation by a native tick (Acari: Ixodidae) on the Australian emu (*Dromaius novaehollandiae*) and a review of tick paralysis in Australian birds. Exp. Appl. Acarol..

[230] Moon K.L., Chown S.L., Loh S.M., Oskam C.L., Fraser C.I. (2018). Australian penguin ticks screened for novel *Borrelia* species. Ticks Tick-Borne Dis..

[231] Moon K.L., Banks S.C., Fraser C.I. (2015). Phylogeographic structure in penguin ticks across an ocean basin indicates allopatric divergence and rare trans-oceanic dispersal. PLoS ONE.

[232] Wicks R.M., Spencer P.B.S., Moolhuijzen P., Clark P. (2006). Morphological and molecular characteristics of a species of *Hepatozoon* Miller, 1908 (Apicomplexa: Adeleorina) from the blood of *Isoodon obesulus* (Marsupialia: Peramelidae) in Western Australia. Syst. Parasitol..

[233] Short M., Lowe K., Michie M., Smith I., Blasdell K., Maier A.G., Gofton A.W. (2024). Tick-borne piroplasms and trypanosomes incidentally detected in eastern grey kangaroos (*Macropus giganteus*) during a mortality and morbidity event in southern New South Wales, Australia. Int. J. Parasitol. Parasites Wildl..

[234] Tricco A.C., Lillie E., Zarin W., O’Brien K.K., Colquhoun H., Levac D., Moher D., Peters M.D., Horsley T., Weeks L. (2018). PRISMA extension for scoping reviews (PRISMA-ScR): Checklist and explanation. Ann. Intern. Med..

