# Supplementary material for: Ticks and Tick-Borne Microorganisms in Australian Wildlife: A Scoping One Health Evidence Synthesis of Reported Associations and Knowledge Gaps
Source: Pathogens. 2026 Jun 18;15(6):646. doi: 10.3390/pathogens15060646 (PMC13305428; doi:10.3390/pathogens15060646)
Supplement: Supplementary file 1 [file pathogens-15-00646-s001.zip › pathogens-4362941-Table S1+ Table S2.pdf]

**Supplementary Table S1.** Wildlife hosts and their associated tick species, as reported in the reviewed studies. Each entry includes tick species (with life stages where reported), study location(s), identification method(s), and tick occurrence data (where available).

| Wildlife species | Tick species (life-stages reported) | Location(s) | Method(s) of Identification | Occurrence (% infested animals) [# infested/ # sampled] | Reference |
|---|---|---|---|---|---|
| **Mammals** | | | | | |
| Short-beaked Echidna (*Tachyglossus aculeatus*) | *Bothriocroton concolor* (adults [m+f]) | Beerwah, QLD | Morphological | Not reported | [137] |
| | | Narre Warren, VIC | | | |
| | | Wagga Wagga, NSW | | | |
| | *B. concolor* (adults [m+f]) | Narre Warren, VIC | Morphological | Not reported | [36] |
| | | Wagga Wagga, NSW | | | |
| | | QLD (Unspecified) | | | |
| | *H. bancrofti* (nymph) | | | | |
| | *B. concolor* (adults [m+f], nymph, larvae) | NSW (Heathcote, Toolom, Biddon, Bateman's Bay, Uralla, Sydney) | Morphological | Not reported (archived samples) | [40] |
| | | QLD (Nanango, Bowen, Clermont, Warwick, Anakie, Brisbane, Boompa, Woolonga, Yeppoon, Cinnabar Goomeri, Gatton, Maleny, Redland Bay, Mt. Abbott, Joyner, Mt. Ommaney, Mt. Isa, Pinjarra Hills, Samford, Running Creek North Arm, Buccan, Lethebrook, Nerang, Port Stewart, Parkhurst, Rockhampton, Chinchilla, Grace Lithgow, Ormiston, South of Mooloolaba, Forestdale, Karana Downs, Queen Mary Falls, Ipswich, Highvale, Malanda, Rural Queensland) | | | |
| | | VIC (Monbulk-Macclesfield, Bathurst Teachers, Healesville Sanctuary, Nowa Nowa, Bathurst Teachers College, Whitlands + unspecified) | | | |
| | | SA (Kangaroo Island, Struan, Lower Southeast, Birdwood Adelaide, Mt. Lofty Ranges, Millicent Lower Southeast, + unspecified) | | | |
| | *B. tachyglossi* (adults [f],larvae) | QLD (Rockhampton, Warwick, Boompa, Woolonga, Yeppoon, Cinnabar Goomeri) | | | |
| | *B. hydrosauri* (adult [m], nymph) | Rockhampton, QLD | | | |
| | *I. tasmani* (adults [f], nymph, larvae) | TAS (Fingal, Antill Ponds, Maydena, Scamander) | | | |
| | *Am. australiense* (adults [m+f], nymph) | - Bowen, QLD<br>- Darwin, NT<br>- Mt. Isa, QLD | | | |
| | *Am. echidnae* (adult [m]) | Townsville, QLD | | | |
| | *Am. triguttatum* (adults [m+f]) | - Lake Hart Gairdner / Torrens Basin, SA<br>- Bowen, QLD | | | |
| | *Am. fimbriatum* (adults [m+f]) | Justa Creek [China Wall], NT | | | |
| | *B. concolor* | VIC (unspecified) | Not reported | 9.8% (n = 8/82) | [206] |
| | *B. concolor* (adults [f]) | Bunyip, VIC | Morphological | Not reported | [72] |
| | | Healesville, VIC | | | |
| | *I. barkeri* (adults [m+f], nymphs, larvae) | Tarzali, QLD | Morphological | Not reported | [41] |
| | | Topaz, QLD | | | |

| | | | | | |
|---|---|---|---|---|---|
| | | Northern QLD | | | |
| | *B. tachyglossi* (adults [m+f], nymphs, larvae) | QLD (Rockhampton, Wowan, Boompa, Monto, Gladstone, Woolooga, Biloela, Emu Park, Yeppoon, Stanwell, St. Lawrence, Mackay, Marian) | - Morphological<br>- Multi-locus enzyme electrophoresis | Not reported | [71] |
| | *I. holocyclus* (adult [f]) | NSW (unspecified) | Morphological | Not reported | [39] |
| | *B. concolor* | VIC (Heyfield, Bairnsdale, Orbost) | - Morphological<br>- Molecular (cPCR [COI], and Sanger sequencing) | Not reported | [80] |
| | *B. concolor* | French Island National Park, VIC | Morphological | Not reported | [207] |
| Echidna (unidentified) | *B. tachyglossi* (adults) | Central QLD (Fitzroy, Central West) | Morphological | Not reported | [55] |
| Platypus (*Ornithorhynchus anatinus*) | *I. ornithorhynchi* | Inglis Catchment, TAS | Morphological | 96.1%<br>(n = 147/153) | [178] |
| | *I. ornithorhynchi* (adult [f], nymph) | Jackson Creek [Sunbury], VIC | Morphological | Not reported | [177] |
| | *I. ornithorhynchi* (adult [f], nymph) | Sandy Bay, TAS<br>QLD (unspecified) | Morphological | Not reported | [36] |
| | *I. ornithorhynchi* (adults [f], nymphs, larvae) | NSW (Abercrombie River, Upper Murrumbidgee River, Queanbeyan River) | Not reported | 100% (n = 4/4) | [97] |
| | *I. ornithorhynchi* | QLD (unspecified) | Morphological | Not reported | [34] |
| | *I. ornithorhynchi* | Inglis Rivers, TAS | Morphological | 36.8% (n = 7/19) | [197] |
| Western Grey Kangaroo (*Macropus fuliginosus*) | *I. australiensis* (adults [m+f], nymphs) | WA (Lowlands, Albany, Manjimup) | Morphological | Not reported | [179]<br>[36] |
| | *Am. triguttatum* (adults [m+f]) | WA (Kulikup, Gidgegannup, Manjimup) | Morphological | Not reported | [36]<br>[179] |
| | *Am. triguttatum* | Innes NP [Southern Yorke Peninsula], SA | Morphological | 50% (n = 2/4) | [59] |
| | *I. australiensis* (adult [f]) | Green Range, WA | Not reported | Not reported | [41] |
| Red Kangaroo (*Osphranter rufus*) | *Am. triguttatum* (adults [m]) | Broome, WA | Morphological | Not reported | [179] |
| | *Am. triguttatum* (adults [m+f]) | WA (unspecified) | Morphological | Not reported | [36] |
| Eastern Grey Kangaroo (*Macropus giganteus*) | *I. holocyclus* (adult [m+f], nymph) | Mallacoota, VIC | Morphological | Not reported | [36] |
| | *H. petrogalis* (adult [m+f]) | Calliope, QLD | | | |
| | *H. lagostrophi* (adult [f]) | | | | |
| | *H. bancrofti* (adults) | Central QLD (Fitzroy, Central West) | Morphological | Not reported | [55] |
| | *I. holocyclus* (adults) | | | | |
| | *Am. triguttatum* | Northern QLD (unspecified) | Morphological | 52.9% (n = 9/17) | [142] |
| | *H. bancrofti* | QLD (unspecified) | Morphological | Not reported | [34] |
| Lumholtz Tree Kangaroo (*Dendrolagus lumholtzi*) | *Haemaphysalis* sp. | QLD (North, South, West); unspecified | Morphological | Not reported | [75] |
| | *I. holocyclus* | | | | |
| | *I. tasmani* | | | | |
| "Macropus sp." (unidentified) | *I. holocyclus* (adult [f]) | NSW (unspecified) | Morphological | Not reported | [39] |
| Tammar Wallaby (*Notamacropus e. eugenii; Macropus eugenii*) | *Ixodes hirsti* (adults [m+f], nymphs) | Innes NP [Southern Yorke Peninsula], SA | Morphological | 20.8%<br>(n = 26/125) | [74] |
| | *Amblyomma triguttatum* (adults [f], nymphs, larvae) | | | | |
| | *Amblyomma* sp. (larvae) | | | | |

| | | | | | |
|---|---|---|---|---|---|
| | *Amblyomma* sp. (larvae) | Upper Warren Range, SA | Morphological | Not reported | [69] |
| | *Am. triguttatum* (larvae) | Innes NP [Southern Yorke Peninsula], SA | Morphological | 21.1% (n = 4/19) | [59] |
| | *I. hirsti* | | | | |
| Red-necked Wallaby (*Notamacropus rufogriseus*) | *Haemaphysalis bancrofti* (adult [m+f], nymph) | - Rock Valley, NSW<br>- Stony Chute, NSW<br>- QLD (unspecified) | Morphological | Not reported | [36] |
| | *I. holocyclus* (adults [f], nymph) | | | | |
| | *I. fecialis* (nymph) | Rock Valley, NSW | | | |
| | | NSW (unspecified) | | | |
| | *I. tasmani* (adults [m+f]) | TAS (unspecified) | | | |
| | *I. tasmani* | Flinders Island (Bass Strait), TAS | Not reported | Not reported | [148] |
| | *Ixodes* sp. | | | | |
| | *I. holocyclus* | QLD (unspecified) | Morphological | Not reported | [34] |
| | *H. bancrofti* | | | | |
| | *H. longicornis* | | | | |
| Swamp Wallaby (*Wallabia bicolor*) | *H. petrogalis* | QLD (unspecified) | Morphological | Not reported | [36] |
| | *I. tasmani* | VIC (unspecified) | | | |
| Agile Wallaby (*Notamacropus agilis*) | *Am. triguttatum* | Northern QLD (unspecified) | Morphological | 80% (n = 4/5) | [142] |
| | *I. confusus* (adults [f]) | Cardwell Forest, QLD | Morphological | Not reported | [52] |
| Bridled Nailtail Wallaby (*Onychogalea fraenata*) | *Am. triguttatum* | Taunton National Park, QLD | Morphological | 96.4% (n = 53/55) | [107] |
| | *H. bancrofti* | | | | |
| Brush-tailed Rock-Wallaby (*Petrogale penicillata*) | *I. holocyclus* (adults [m+f], nymphs, larvae) | QLD (Hurdle Creek, Fern Creek, Farm Creek, Mount Colliery) | Morphological | 75.8% (n = 88/116) | [99] |
| | *H. bancrofti* (adults [m+f], nymphs, larvae) | | | | |
| 'Wallaby' (unidentified) | *H. bancrofti* | QLD (North, South, West); unspecified | Morphological | Not reported | [75] |
| | *Ixodes* sp. | | | | |
| | *I. holocyclus* | | | | |
| Wallaroo (*Osphranter robustus*) | *H. bancrofti* | Central QLD (Fitzroy, Central West) | Morphological | Not reported | [55] |
| | *Am. triguttatum* | Northern QLD (unspecified) | Morphological | Not reported | [142] |
| Koala (*Phascolarctos cinereus*) | *I. tasmani* (adults [m+f]) | Koala Conservation Centre [Phillip Island], VIC | Morphological | Not reported | [208] |
| | *I. trichosuri* (adult [f]) | | | | |
| | *I. tasmani* | - QLD (unspecified)<br>- NSW (unspecified) | Morphological | Not reported | [34] |
| | *I. holocyclus* | | | | |
| | *I. tasmani* (adults [m+f]) | Phillip Island, VIC | Morphological | Not reported | [72] |
| | *I. holocyclus* (adults [m+f]) | QLD (unspecified) | Morphological | Not reported | [67] |
| | *I. tasmani* (adults [m+f]) | | | | |
| | *I. holocyclus* (adults [f]) | Lamington National Park, QLD | Not reported | Not reported | [96] |
| | *I. tasmani* (adults [f]) | | | | |
| | *I. tasmani* | Portland, VIC | - Morphological<br>- Molecular (cPCR [COI + 16S rRNA]; then Sanger sequencing) [tick samples] | Not reported | [32] |
| | *I. tasmani* | Port Macquarie, NSW | Morphological | Not reported | [157] |
| | *Haemaphysalis* sp. (*H. bancrofti*) | Raymond Island, VIC | Morphological | Not reported | [207] |
| | | | - Morphological<br>- Molecular (cPCR [COI], and Sanger sequencing) | Not reported | [80] |
| | *I. hirsti* | Bairnsdale, VIC | | | |

| | | | | | |
|---|---|---|---|---|---|
| | | | | | |
| | *I. hirsti* | VIC (French Island National Park, Mt. Eccles, Framlingham Forest) | Morphological | Not reported | [209] |
| | | Kangaroo Island, SA | | | |
| Tasmanian Pademelon (*Thylogale billardierii*) | *I. tasmani* (adult [f]) | Port Sorell, TAS | Morphological | Not reported | [36] |
| | | Nicholls Rivulet, TAS | | | |
| Red-legged Pademelon (*Thylogale stigmatica*) | *I. confusus* (adults [m+f]) | Mt. Molloy, QLD | Morphological | Not reported | [52] |
| Quokka (*Setonix brachyurus*) | *I. australiensis* | Bald Island, WA | Morphological | Not reported | [185] |
| | *I. hirsti* | | | | |
| | *I. myrmecobii* | | | | |
| Long-footed Potoroos (*Potorous longipes*) | *I. victoriensis* | VIC (Orbost, Cabbage Tree Creek) | Morphological | Not reported | [47] |
| Gilbert's Potoroo (*Potorous gilbertii*) | *I. australiensis* | Two Peoples Bay, WA | Morphological | Not reported | [185] |
| | *I. australiensis* (adults, nymphs) | | | | [183] |
| | *I. fecialis* (adult) | | | | |
| Long-nosed Potoroo (*Potorous tridactylus*) | *H. bancrofti* | NSW (Nadgee, Timbillica, East Boyd) | Morphological | Not reported | [210] |
| | *I. holocyclus* | | | | |
| | *I. victoriensis* | Maggs Mountain, TAS | Morphological | Not reported | [47] |
| | *I. hirsti* (adult [m+f]) | French Island National Park, VIC | Morphological | Not reported | [209] |
| Northern Bettong (*Bettongia tropica*) | *Amblyomma* sp. | QLD (North - unspecified) | Morphological | Not reported | [75] |
| | *Haemaphysalis* sp. | | | | |
| | *H. bancrofti* | | | | |
| | *Ixodes* sp. | | | | |
| | *I. holocyclus* | | | | |
| | *I. tasmani* | | | | |
| Brush-tailed Bettong ['Woylie'] (*Bettongia penicillata*) | *I. woyliei* (adults [f], nymph) | WA (Karakamia Wildlife Sanctuary, Dryandra Woodlands, Upper Warren Region) | - Morphological; + electron microscopy<br>- Molecular (cPCR [COI]; then Sanger sequencing) | 19.7% (n = 123/624) | [42] |
| | *I. australiensis* (adult [f], nymph) | | Morphological | 24.5% (n = 153/624) | |
| | *I. myrmecobii* (adult [m]) | | | 8.3% (n = 52/624) | |
| | *I. tasmani* | | | 0.64% (n = 4/624) | |
| | *I. fecialis* | | | 0.16% (n = 1/624) | |
| | *Am. triguttatum* (nymph) | Upper Warren Region, WA | Morphological | Not reported | [69] |
| | *I. woyliei* (adults [f], nymphs) | | | | |
| | *I. tasmani* (adults [f], nymph) | | | | |
| | *I. myrmecobii* (adults [f], nymph) | | | | |
| | *Ixodes sp* (larvae) | | | | |
| | *Amblyomma* sp. (larvae) | | | | |
| | *Am. triguttatum* | WA (Keninup, Balban, Karakamia) | Morphological | Not reported | [211] |
| | *I. tasmani* | | | | |
| | *I. myrmecobii* | | | | |

| | | | | | |
|---|---|---|---|---|---|
| | *I. australiensis* | | | | |
| | *Ixodes* spp. | | | | |
| Rufous Bettong (*Aepyprymnus rufescens*) | *Am. triguttatum* | Northern QLD (unspecified) | Morphological | 100% (n = 1/1) | [142] |
| Eastern Bettong (*Bettongia gaimardi*) | *I. tasmani* | TAS (Long Hill, Pipers River, Mt. Morrison, Bangor, The Lea, Grove, Castle Forbes Bay, Geeveston) | Morphological | 92% (n = 55/60) | [212] |
| | *I. cornuatus* | | | | |
| | *I. trichosuri* | | | | |
| Bare-nosed Wombat (*Vombatus ursinus*) | *B. auruginans* (adults [m+f], nymph) | Wee Jasper, NSW | Morphological | Not reported | [36] |
| | | Trafalgar, VIC | | | |
| | | TAS (unspecified) | | | |
| | *I. tasmani* (adults [f]) | Orbost, VIC | | | |
| | *I. holocyclus* (adults [f]) | | | | |
| | *Aponomma* sp. [*Bothriocroton*] | Flinders Island (Bass Strait), TAS | Not reported | Not reported | [148] |
| | *Ixodes* sp. | | | | |
| | *Aponomma* [*Bothriocroton*] auruginans | Gippsland, VIC | | | |
| | *B. auruginans* | TAS (unspecified) | Morphological | Not reported | [34] |
| | *I. tasmani* | Not reported | | | |
| | *I. cornuatus* (adults [f]) | Cape Patterson, VIC | - Morphological<br>- Multilocus enzyme electrophoresis | Not reported | [53] |
| | *I. holocyclus* (adult [f]) | | | | |
| | *B. auruginans* (adult [f]) | Buxton, VIC | Morphological | Not reported | [72] |
| | *I. victoriensis* | VIC (Marysville, Mt. Buller, Tolmie, Myers Creek) | Morphological | Not reported | [47] |
| | *B. auruginans* (adults [m+f], nymphs) | NSW (Coolagilite, Rock Flat, Yowrie, Bellmount Forest, Bilpin, Bemboka, Buxton, Primrose Valley, Courijah, Orangville, Bungendore, Burra, Gelstone Park, Dalgety, Kangaroo Valley, Mowbray Park, Pheasant Nest, Picton, Quaama, The Oaks, Mount Hunter, The Rock, Wagga Wagga, Thirlmere, Wolgan Valley) | Morphological | Not reported | [50] |
| | *H. bancrofti* (adults [f], nymphs) | NSW (Coolagilite, Dalgety, Picton, Quaama) | | | |
| | *H. longicornis* (adults [m+f]) | Coolagilite, NSW | | | |
| | *I. tasmani* (adults [f], nymph) | Cradle Mountain, TAS | | | |
| | | Dalgety, NSW | | | |
| | *I. holocyclus* (adults [f]) | NSW (Coolagilite, Dalgety, Quaama) | | | |
| | *Ap.* (*Bothriocroton*) *auruginans* | Wilson's Promontory, VIC | - Morphological<br>- Molecular (cPCR [COI + 16S rRNA]; then Sanger sequencing) [tick samples] | Not reported | [32] |
| | *B. auruginans* | Glenburn, VIC | - Morphological<br>- Multi-locus enzyme electrophoresis | Not reported | [71] |
| | *I. cornuatus* | Raymond Island, VIC | - Morphological<br>- Molecular (cPCR [COI], and Sanger sequencing) | Not reported | [80] |

| | | | | | |
|---|---|---|---|---|---|
| Eastern-Barred Bandicoot (*Perameles gunnii*) | *I. tasmani* (adult [f], nymph) | TAS (Seven Mile Beach, Devonport, Ridgeway) | Morphological | Not reported | [36] |
| | *I. tasmani* (adults [f], nymphs) | TAS (Devonport, Ridgeway) | Morphological | Not reported | [64] |
| Long-nosed Bandicoot (*Perameles nasuta*) | *I. holocyclus* (adult [m+f], nymph) | NSW (Boxhill, Murrah, Sydney, Boorie Creek, Stony Chute, Manly, Pearl Beach, Castlecrag) | Morphological | Not reported | [36] |
| | *H. bancrofti* (adult [f], nymph) | NSW (Murrah, Stony Chute, Castlecrag) | | | |
| | *H. humerosa* (nymph) | Stony Chute, NSW | | | |
| | *I. tasmani* (nymph) | Sydney, NSW | | | |
| | *I. holocyclus* (adults [m+f], nymphs, larvae) | NSW (Nareen Reserve, Stapleton Reserve, Turimetta Headland, Warriewood Wetlands) | - Morphological<br>- Molecular (cPCR; PCR [amplify]; then NGS [Illumina])<br>[tick 12S rRNA] | 70.5% (n = 31/44) | [51] |
| | *I. tasmani* (nymphs, larvae) | NSW (Dobroyd Head, Nareen Reserve, Stapleton Reserve, Turimetta Headland, Warriewood Wetlands) | | | |
| | *I. trichosuri* (nymphs, larvae) | NSW (North Head, Warriewood Wetlands) | | | |
| | *I. tasmani* | QLD (unspecified) | Morphological | Not reported | [34] |
| | *I. holocyclus* | | | | |
| | *H. humerosa* | | | | |
| | *I. holocyclus* | Northern Beaches [Sydney], NSW | Morphological | Not reported | [95] |
| | *I. holocyclus* (adult, nymph) | Sydney, NSW | - Morphological<br>- Molecular (NGS (COI) [Illumina]) | Not reported | [86] |
| | *I. holocyclus* (adults, nymphs, larvae) | Northern Beaches LGA (Sydney), NSW | - Morphological<br>- Molecular (NGS [Illumina] -- immature) | Not reported | [33] |
| | *I. tasmani* (adult, nymphs, larvae) | | | | |
| | *I. trichosuri* (nymphs, larvae) | | | | |
| | *I. hirsti* (nymph) | | | | |
| | *I. holocyclus* (nymphs) | Castlecrag, NSW | Morphological | Not reported | [64] |
| | *H. bancrofti* (nymph) | | | | |
| | *I. holocyclus* (nymph) | Sydney, NSW | | | |
| | *I. tasmani* (nymphs) | | | | |
| | *I. holocyclus* (adults [m+f]) | NSW (Boorie Creek, Boxhill) | | | |
| | *I. holocyclus* (nymphs) | Murrah, NSW | | | |
| | *H. bancrofti* (nymph) | | | | |
| | *H. bancrofti* (nymph) | Stony Chute, NSW | | | |
| | *H. humerosa* (nymph) | | | | |
| | *I. holocyclus* | Manly, NSW | | | |
| | *I. holocyclus* (nymphs) | Beerwah, QLD | | | |
| | *I. tasmani* | | | | |
| Northern Brown Bandicoot (*Isoodon macrourus*) | *I. holocyclus* (adult [f], nymph) | Not reported | Morphological | Not reported | [36] |
| | *H. humerosa* (adult [m+f], nymph) | Palmerston, NT | | | |
| | | Bees Creek, NT | | | |
| | *I. tasmani* (adult [f]) | QLD (unspecified) | | | |
| | *Haemaphysalis* sp. | QLD (North, South, or West); unspecified | Morphological | Not reported | [75] |

| | | | | | |
|---|---|---|---|---|---|
| | *H. bancrofti* | | | Not reported | |
| | *I. holocyclus* | | | Not reported | |
| | *I. holocyclus* | Northern QLD (unspecified) | Morphological | 97.1% (n = 34/35) | [142] |
| | *H. humerosa* | | | | |
| | *I. holocyclus* (females) | Beerwah, QLD | Morphological | Not reported | [64] |
| | *H. humerosa* (adult [f]) | | | | |
| | *H. humerosa* (adults [m+f], nymphs) | Palmerston, NT<br>Bees Creek, NT | | | |
| Southern Brown Bandicoot (*Isoodon obesulus*) | *I. holocyclus* (adults, nymphs) | Timbillica, NSW | - Morphological<br>- Molecular (NGS (COI) [Illumina]) | Not reported | [86] |
| | *I. tasmani* | Koo Wee Rup, VIC | - Morphological<br>- Molecular (cPCR [COI + 16S rRNA]; then Sanger sequencing) [tick samples] | Not reported | [32] |
| | *I. trichosuri* | | | | |
| | *I. trichosuri* | Cranbourne, VIC | | | |
| Quenda (*Isoodon fusciventer*) | *I. woyliei* | - Dryandra Woodland, WA<br>- Upper Warren, WA | Morphological | 15.4% (n = 2/13) | [42] |
| | *I. australiensis* | | | 15.4% (n = 2/13) | |
| | *I. myrmecobii* | | | 7.7% (n = 1/13) | |
| | *I. fecialis* (adult [f]) | Albany, WA | Not reported | Not reported | [41] |
| | *I. woyliei* (adult [f]) | | | | |
| | *I. fecialis* (adults [f]) | Albany, WA | Morphological | Not reported | [64] |
| | *I. australiensis* (adult [f]) | | | | |
| | *I. fecialis* (adult [f]) | Maida Vale, WA | | | |
| | *I. fecialis* (adult [f]) | Maida Vale, WA<br>Albany, WA | Morphological | Not reported | [36] |
| | *I. australiensis* (adult [f], nymph) | Walcott, WA<br>Warrup East, WA | | | |
| | *I. myrmecobii* (nymph) | | | | |
| | *Am. triguttatum* (nymphs) | Whiteman Park, WA | - Morphological<br>- Molecular (cPCR; PCR [amplify]; then NGS [Illumina])<br>[tick 12S rRNA] | 100% (n = 2/2) | [51] |
| | *I. australiensis* (nymph) | | | | |
| | *I. fecialis* (adults [f]) | Upper Warren, WA | Morphological | 100% (n = 1/1) | [69] |
| | *I. fecialis* | WA (various locations -- incl. bushland [BL] and urbanized [UR])<br>- Fremantle, WA<br>- Armadale, WA<br>- Mundaring, WA<br>- Perth, WA | Morphological | BL: 7.5% (n =12/161)<br>UR: 36.5% (n = 46/126) | [124] |
| | *I. australiensis* | | | BL: 7.5% (n =12/161)<br>UR: 6.3% (n = 8/126) | |
| | *H. humerosa* | | | BL: 5.6% (n = 9/161)<br>UR: 0.8% (n = 1/126) | |
| | *Amblyomma* sp. | | | BL: 19.9% (n =32/161)<br>UR: 12.7% (n = 16/126) | |
| Western-Barred Bandicoot (*Perameles bougainville*) | *H. humerosa* (adult [m+f]) | - Dorre, WA<br>- Bernier Island, WA | Morphological | Not reported | [60] |

| | | | | | |
|---|---|---|---|---|---|
| | *H. ratti* (adults [f]) | | | | |
| | *H. lagostrophi* | | | | |
| 'Bandicoot' (unidentified) | *H. humerosa* (nymph) | Central QLD (Fitzroy, Central West) | Morphological | Not reported | [55] |
| | *I. tasmani* (adult [f]) | Port Sorell, TAS | Morphological | Not reported | [64] |
| | | Beerwah, QLD | | | |
| Greater Bilby (*Macrotis lagotis*) | *I. woyliei* | Kanyana Wildlife Rehabilitation Centre [Perth], WA | Morphological | 100% (n = 1/1) | [42] |
| Brush-tailed Possum (*Trichosurus vulpecula*) | *I. tasmani* (adult [f], nymph) | TAS (unspecified) | Morphological | Not reported | [36] |
| | | Royal Botanical Garden, NSW | | | |
| | *I. holocyclus* (adult [m+f], nymph) | QLD (unspecified) | | | |
| | *I. trichosuri* (adult [f]) | | | | |
| | *I. hirsti* (adult [f]) | NSW (unspecified) | | | |
| | *Ixodes* sp. | QLD (North, South, West); unspecified | Morphological | Not reported | [75] |
| | *I. holocyclus* | | | | |
| | *I. australiensis* | - Dryandra Woodland, WA<br>- Upper Warren, WA | Morphological | 0.6% (n = 1/166) | [42] |
| | *I. myrmecobii* (nymph) | | | 1.8% (n = 3/166) | |
| | *I. tasmani* (adult [f], nymph) | | | 32.5% (n = 54/166) | |
| | *I. australiensis* (adults [m+f], nymph) | Upper Warren, WA | Morphological | Not reported | [69] |
| | *I. myrmecobii* (adults [f], nymph) | | | | |
| | *I. tasmani* (adults [f], nymph) | | | | |
| | *Am. triguttatum* (larvae) | Julimar State Forest, WA | - Morphological<br>- Molecular (cPCR; PCR [amplify]; then NGS [Illumina])<br>[tick 12s rRNA] | 55.6% (n = 15/27) | [51] |
| | *I. holocyclus* (adults [f], nymphs) | NSW (Stapleton Reserve, Dobroyd Head, North Head) | | | |
| | *I. tasmani* (adults [f]) | North Head, NSW | | | |
| | *I. trichosuri* (adults [f], nymph, larvae) | | | | |
| | *I. tasmani* | Perth, WA | Morphological | 5.6% (n = 1/18) | [124] |
| | *H. bremneri* | | | 5.6% (n = 1/18) | |
| | *Amblyomma* sp. | | | 22.2% (n = 4/18) | |
| | Ixodidae (larvae) | | Unidentified | 38.9% (n = 7/18) | |
| | *I. tasmani* | QLD (unspecified) | Morphological | Not reported | [34] |
| | *I. holocyclus* | | | | |
| | *I. tasmani* | NSW (Taronga Zoological Park [Mosman], Jenolan Caves Reserve Trust land [Blue Mountains]) | Morphological | Urban: 19.5% (n = 47/241)<br>Woodland: 13.5% (n = 5/37) | [123] |
| | *I. trichosuri* | | | Urban: 18.7% (n = 45/241)<br>Woodland: 21.6% (n = 8/37) | |
| | *I. holocyclus* | | | Urban: 3.7% (n = 9/241)<br>Woodland: 2.7% (n = 1/37) | |

| | | | | | |
|---|---|---|---|---|---|
| | *I. holocyclus* (adults, nymphs) | Northern Beaches LGA (Sydney), NSW | - Morphological<br>- Molecular (NGS [Illumina] -- immature) | Not reported | [33] |
| | *I. tasmani* (adults, nymphs, larva) | | | | |
| | *I. trichosuri* (adults, nymphs, larva) | | | | |
| | *I. hirsti* (adults) | | | | |
| | *I. trichosuri* (adult [f]) | NSW (unspecified) | Not reported | Not reported | [52] |
| | *I. holocyclus* (adults [m+f]) | Beerwah, QLD | Morphological | Not reported | [64] |
| | *I. tasmani* (adults [f]) | | | | |
| | *I. trichosuri* (adults [f]) | | | | |
| | *I. tasmani* (adult [f]) | Royal Botanic Gardens, NSW | | | |
| | *I. holocyclus* (adult [f]) | NSW (unspecified) | Morphological | Not reported | [39] |
| Ring-tailed Possum (*Pseudocheirus peregrinus*) | *I. tasmani* (adult [f], nymph) | Turramurra, NSW<br>TAS (unspecified)<br>QLD (unspecified) | Morphological | Not reported | [36] |
| | *I. holocyclus* (adult [f]) | QLD (unspecified) | | | |
| | *I. tasmani* | QLD (unspecified) | Morphological | Not reported | [34] |
| | *I. holocyclus* (adult [f]) | Beerwah, QLD | Morphological | Not reported | [64] |
| | *I. tasmani* (adults [f]) | | | | |
| | *I. tasmani* (adults [f]) | NSW (Neutral Bay, Turramurra) | | | |
| | *I. tasmani* (nymph) | Sandy Bay, TAS | | | |
| Leadbeater's Possum (*Gymnobelideus leadbeateri*) | *I. tasmani* | Yellingbo Nature Conservation Reserve, VIC [lowland]<br>Toorongo Plateau, VIC [highland] | Morphological | Not reported | [77] |
| Mountain brush-tailed possum (*Trichosurus cunninghami*) | *I. trichosuri* | Boho South, VIC | - Morphological<br>- Molecular (cPCR [COI + 16S rRNA]; then Sanger sequencing) [tick samples] | Not reported | [32] |
| | *I. tasmani* | | | | |
| Possum (unidentified sp.) | *I. holocyclus* (adult [f], nymph) | QLD (unspecified) | Morphological | Not reported | [36] |
| | *H. humerosa* (adult [f]) | | | | |
| | *H. bremneri* (adult [f]) | | | | |
| | *Ixodes* sp. | QLD (North, South, West); unspecified | Morphological | Not reported | [75] |
| | *I. holocyclus* | | | | |
| | *I. holocyclus* | Brisbane, QLD | Not reported | Not reported | [213] |
| | *I. holocyclus* (adult [f], nymph) | Beerwah, QLD | Morphological | Not reported | [64] |
| | *H. bremneri* (adults [f]) | | | | |
| | *H. humerosa* (adults [f]) | | | | |
| Short-eared Possum (*Trichosurus caninus*) | *I. holocyclus* (adults [m+f]) | Nambour, QLD | Morphological | Not reported | [36] |
| | *I. tasmani* (adults [f]) | QLD (unspecified) | | | |
| | *I. trichosuri* (adults [f]) | | | | |
| | *I. tasmani* | QLD (unspecified) | Morphological | Not reported | [34] |
| | *I. holocyclus* (adults [m+f]) | Beerwah, QLD | Morphological | Not reported | [64] |
| | *I. tasmani* (adult [f]) | | | | |
| | *I. heathi* | Mt. Loch, VIC | Morphological | Not reported | [49] |

| | | | | | |
|---|---|---|---|---|---|
| Mountain Pygmy Possum (*Burramys parvus*) | | Mt. Higginbotham, VIC | | | |
| Striped Possum (*Dactylopsila trivirgata*) | *I. holocyclus* | Atherton, QLD | Morphological | Not reported | [214] |
| | *I. cordifer* | QLD (Atherton, Shiptons Flat, Daintree) | | | |
| Sugar Glider (*Petaurus breviceps*) | *I. tasmani* (adults [f], nymph) | QLD (unspecified) | Morphological | Not reported | [36] |
| | *I. holocyclus* (adults [f]) | | | | |
| Squirrel glider (*Petaurus norfolcensis*) | *I. tasmani* | QLD (unspecified) | Morphological | Not reported | [34] |
| Spotted Quoll (*Dasyurus maculatus*) | *I. fecialis* (adults [f]) | QLD (unspecified) | Morphological | Not reported | [36] |
| | *I. holocyclus* | NSW (unspecified) | Morphological | Not reported | [34] |
| | *I. tasmani* (adults [f]) | New England Tablelands, NSW | Morphological | Not reported | [30] |
| | *I. holocyclus* (adults [m+f], nymphs) | | | | |
| Northern Quoll (*Dasyurus hallucatus*) | *I. holocyclus* | QLD (North, South, West); unspecified | Morphological | Not reported | [75] |
| | *Ixodes* spp. | | | | |
| | *I. tasmani* | Walsh River Range, QLD | Morphological | 73.1% (n = 19/26)<br>- Walsh: 44.4% (n = 4/9)<br>- Davies Creek: 80% (n = 4/5)<br>- Tinaroo: 91.7% (n.= 11/12) | [215] |
| | *I. fecialis* | QLD (Davies Creek, Tinaroo Creek) | | | |
| | *I. holocyclus* | Tinaroo Creek, QLD | | | |
| | *H. humerosa* | QLD (Walsh River Range, Davies Creek, Tinaroo Creek) | | | |
| | *H. humerosa* | Kakadu National Park, NT | Morphological | Not reported | [216] |
| | *H. bancrofti* | | | | |
| | *H. humerosa* | Shark Bay [Bernier and Dorre Island], WA | Not reported | Not reported | [60] |
| | *H. ratti* | | | | |
| Western Quoll [‘Chuditch’] (*Dasyurus geoffroii*) | *I. australiensis* | - Dryandra Woodland, WA<br>- Upper Warren, WA | Morphological | 14.6% (n = 13/89) | [42] |
| | *I. tasmani* | | | 7.9% (n = 7/89) | |
| | *I. fecialis* (adult [f]) | | | 10.1 % (n = 9/89) | |
| | *Am. triguttatum* (larva) | Avon Valley, WA | - Morphological<br>- Molecular (cPCR; PCR [amplify]; then NGS [Illumina])<br>[tick 12s rRNA] | 4.5% (n = 1/22) | [51] |
| Eastern Quoll (*Dasyurus viverrinus*) | *I. tasmani* | TAS (Bronte, Uxbridge, Judbury, Grove, Geeveston, Cygnet) | Morphological | 25% (n = 4/16) | [182] |
| | *I. fecialis* | | | 12.5% (n = 2/16) | |
| | *I. cornuatus* (misidentified as *I. holocyclus*) | | | 6.25% (n = 1/16) | |
| Tasmanian Devil (*Sarcophilus harrisii*) | *I. tasmani* (adults [f], nymph) | Lower Longley, TAS | Morphological | Not reported | [36] |
| | *I. fecialis* (adults [f], nymph) | West Takone, TAS | | | |
| | *I. tasmani* | NE/E TAS (near Launceston + Hobart) | Morphological | Not reported | [154] |
| | *I. cornuatus* (misidentified as *I. holocyclus*) | TAS (Wall Woolnorth, Surrey Hills, Mount Pleasant, Ida Bay, Bronte Park, Hastings) | Morphological | Not reported | [156] |

| | | | | | |
|---|---|---|---|---|---|
| Brown Antechinus (*Antechinus stuartii*) | *I. holocyclus* (nymphs, larvae) | Murramarang National Park [Kioloa, Creek, Lagoon], NSW | Morphological | Not reported | [108] |
| | *I. tasmani* (nymphs, larvae) | | | | |
| | *I. fecialis* (nymphs) | | | | |
| | *H. ratti* (larvae) | | | | |
| | *I. tasmani* (nymph) | Newres Plateau, NSW | Morphological | Not reported | [36] |
| | *I. antechini* (adult [m+f], nymph) | | | | |
| | *I. tasmani* (nymph) | Pearl Beach, NSW | | | |
| | *I. holocyclus* (nymph) | | | | |
| | *I. antechini* (adult [m+f], nymph) | Dungog, NSW | | | |
| | *I. fecialis* (nymph) | | | | |
| | *I. antechini* (adults [f]) | NSW (Warriewood Wetlands) | - Morphological<br>- Molecular (cPCR; PCR [amplify]; then NGS [Illumina])<br>[tick 12S rRNA] | 80% (n = 4/5) | [51] |
| | *I. tasmani* (nymph, larvae) | | | | |
| | *I. holocyclus* (nymphs, larvae) | Northern Beaches LGA (Sydney), NSW | - Morphological<br>- Molecular (NGS [Illumina] -- immature) | Not reported | [33] |
| | *I. tasmani* (nymphs, larvae) | | | | |
| | *I. trichosuri* (larvae) | | | | |
| | *I. antechini* (adults, nymphs, larvae) | | | | |
| Agile Antechinus (*Antechinus agilis*) | *I. antechini* (adults [m+f], nymph) | Newnes Plateau, NSW | Morphological | Not reported | [36] |
| | *I. antechini* (adults [m+f], nymphs, larvae) | Bullengarook, VIC | - Morphological<br>- Multilocus enzyme electrophoresis | Not reported | [53] |
| | *I. tasmani* | Boho South, VIC | - Morphological<br>- Molecular (cPCR [COI + 16S rRNA]; then Sanger sequencing)<br>[tick samples] | Not reported | [32] |
| | *I. antechini* | | | | |
| Yellow-footed Antechinus (*Antechinus flavipes*) | *I. antechini* | Dwellingup, WA | Morphological | Not reported | [217] |
| Black Rat (*Rattus rattus*) | *I. holocyclus* (adult [f], nymph) | Castlecrag, NSW | Morphological | Not reported | [36] |
| | | Sydney, NSW | | | |
| | *I. tasmani* (nymph) | | | | |
| | *I. holocyclus* (nymphs, larvae) | NSW (Stapleton Reserve, Warriewood Wetlands, Dobroyd Head) | - Morphological<br>- Molecular (cPCR; PCR [amplify]; then NGS [Illumina])<br>[tick 12S rRNA] | 61.4% (n = 54/88) | [51] |
| | *I. tasmani* (larvae, nymphs) | NSW (Manly Dam, Warriewood Wetlands, Stapleton Reserve, Condover Reserve, Turimetta Headland, Nareen Reserve, Dobroyd Head) | | | |
| | *I. holocyclus* (nymphs, larvae) | Gippsland, VIC | - Morphological | Not reported | [53] |

| | | | | | |
|---|---|---|---|---|---|
| | *I. fecialis* (adults [f], nymphs) | | - Multilocus enzyme electrophoresis | | |
| | *I. tasmani* (nymph)? | | | | |
| | *H. longicornis* (nymph, larva) | | | | |
| | *I. holocyclus* | Northern Beaches [Sydney], NSW | Morphological | Not reported | [95] |
| | *Am. triguttatum* | Innes NP [Southern Yorke Peninsula], SA | Morphological | 22.2% (n = 2/9) | [59] |
| | *I. holocyclus* (nymph) | Sydney, NSW | - Morphological<br>- Molecular (NGS (COI) [Illumina]) | Not reported | [86] |
| | *I. holocyclus* (adults, nymphs, larvae) | Northern Beaches LGA (Sydney), NSW | - Morphological<br>- Molecular (NGS [Illumina] -- immature) | Not reported | [33] |
| | *I. tasmani* (nymphs, larvae) | | | | |
| | *I. trichosuri* (nymphs, larvae) | | | | |
| | *I. hirsti* (nymphs, larva) | | | | |
| | *I. fecialis* (nymph, larvae) | | | | |
| | *I. holocyclus* | Atherton Tablelands, QLD | Morphological | 17.6% (n = 3/17) | [218] |
| | *I. holocyclus* (adult [f], nymph) | Castlecrag, NSW | Morphological | Not reported | [64] |
| | *I. tasmani* (nymphs) | Sydney, NSW | | | |
| | *I. holocyclus* (nymph) | | | | |
| | *I. holocyclus* (nymphs, larvae) | North Head [Sydney Harbour National Park], NSW | Morphological | 91.1% (n = 82/90) | [68] |
| | *I. tasmani* (nymphs, larvae) | | | | |
| | *I. hirsti* (nymphs, larvae, adult) | | | | |
| | *I. fecialis* (nymphs, larvae) | | | | |
| | *I. trichosuri* (nymphs, larvae) | | | | |
| | *H. bancrofti* | | | | |
| Bush Rat (*Rattus fuscipes*) | *I. tasmani* (nymph) | Pearl Beach, NSW | Morphological | Not reported | [36] |
| | *I. holocyclus* (nymph) | | | | |
| | *I. trichosuri* | Gippsland, VIC | Not reported | Not reported | [148] |
| | *Ixodes* sp. | | | | |
| | *I. tasmani* (nymphs, larva) | NSW (Warriewood Wetlands, Manly Dam) | - Morphological<br>- Molecular (cPCR; PCR [amplify]; then NGS [Illumina])<br>[tick 12s rRNA] | 100% (n = 3/3) | [51] |
| | *I. cornuatus* (nymphs) | Bullengarook, VIC | - Morphological<br>- Multilocus enzyme electrophoresis | Not reported | [53] |
| | *I. holocyclus* (nymphs, larvae) | Gippsland, VIC | | | |
| | *I. fecialis* (adults [f], nymphs) | | | | |

| | | | | | |
|---|---|---|---|---|---|
| | *I. tasmani* (nymph) | | | | |
| | *H. longicornis* (nymphs, larva) | | | | |
| | *Ixodes* sp. | WA (unspecified) | Morphological | Not reported | [211] |
| | *I. trichosuri* (adult) | Sydney, NSW | - Morphological<br>- Molecular (NGS (COI) [Illumina]) | Not reported | [86] |
| | *I. holocyclus* (nymphs) | Northern Beaches LGA (Sydney), NSW | - Morphological<br>- Molecular (NGS [Illumina] -- immature) | Not reported | [33] |
| | *I. tasmani* (nymphs, larvae) | | | | |
| | *I. tasmani* (nymphs) | Pearl Beach, NSW | Morphological | Not reported | [64] |
| | *Ixodes* sp. (nymphs, larvae) | Mogo State Forest (Kioloa), NSW | Molecular (cPCR, then Sanger sequencing and NGS [Illumina]) | Not reported | [85] |
| Swamp Rat (*Rattus lutreolus*) | *I. hirsti* | Flinders Island (Bass Strait), TAS | Not reported | Not reported | [148] |
| | *I. trichosuri* | Gippsland, VIC | | | |
| | *I. holocyclus* (nymphs, larvae) | Gippsland VIC | - Morphological<br><br>- Multilocus enzyme electrophoresis | Not reported | [53] |
| | *I. fecialis* (adults [f], nymphs) | | | | |
| | *I. tasmani* (nymph) | | | | |
| | *H. longicornis* (nymph, larva) | | | | |
| Water Rat (*Hydromys chrysogaster*) | *I. tasmani* (adult [f]) | QLD (unspecified) | Morphological | Not reported | [36] |
| | *I. holocyclus* (nymph) | | | | |
| | *I. tasmani* | Eagle Point, VIC | Not reported | 3.2% (n = 1/31) | [219] |
| Dusky Rat (*Rattus colletti*) | *H. ratti* | Beatrice Hill Research Farm, NT | Not reported | 14.4% (n = 26/180) | [220] |
| Shark Bay Mouse (*Pseudomys fieldi*) | *H. ratti* (adults [f]) | Bernier Island, WA | Not reported | Not reported | [60] |
| | *H. humerosa* (adults [f]) | | | | |
| 'Bat' (unidentified) | *Rhipicephalus* (*sanguineus*) ***linnaei*** (adult [f]) | Darwin, NT | Morphological | Not reported | [36] |
| | | | | | [64] |
| Flying Fox (*Pteropus* sp.) | *I. holocyclus* (adult [f]) | QLD (unspecified) | Morphological | Not reported | [36] |
| | | Beerwah, QLD | | | [64] |
| Black Flying Fox (*Pteropus alecto*) | *I. tasmani* (adult [f]) | QLD (unspecified) | Morphological | Not reported | [36] |
| | | | | | [64] |
| Spectacled Flying Fox (*Pteropus conspicillatus*) | *I. holocyclus* | QLD (Tolga Scrub, New Powley Road, Whiteng Road, Lakeside) | Not reported | Not reported | [100] |
| | | QLD (Powley Road, Tolga) | | | [98] |
| Southern Bent-winged bat (*Miniopterus orianae bassani*) | *I. simplex* | Allansford, VIC | Morphological | 50% (n = 16/32) | [110] |
| | | Portland, VIC | | 26.8% (n = 30/112) | |
| | | Naracoorte, SA | | 5.1% (n = 7/138) | |
| Eastern Bent-winged bat (*Miniopterus orianae oceanensis*) | *I. simplex* | Eildon, VIC | Morphological | 66.7% (n = 26/39) | [110] |
| Gould's Wattled Bat (*Chalinolobus gouldii*) | *Argas dewae* | Organ Pipes National Park, VIC | Morphological | Not reported | [57] |
| *Vespadelus* spp. | *Ar. dewae* | | | | |

| | | | | | |
|---|---|---|---|---|---|
| Wild (feral) Pig (*Sus scrofa*) | *I. australiensis* (adult [f]) | WA (unspecified) | Morphological | Not reported | [36] |
| | *Am. triguttatum* | Mundaring, WA | Morphological | 56.2% (n = 18/32) | [153] |
| | *Am. triguttatum* | Serpentine, WA | | 61.2% (n = 63/103) | |
| | *I. australiensis* | | | | |
| | *Am. triguttatum* | Dwellingup, WA | | 28.8% (n = 21/73) | |
| | *I. australiensis* | | | | |
| | *Am. cyprium cyprium* | Wet Tropic World Heritage area, QLD | Not reported | 43.9% (n = 134/305) | [221] |
| Red Fox (*Vulpes vulpes*) | *I. holocyclus* (adults [f]) | Grose Vale, NSW | Morphological | Not reported | [36] |
| | *H. longicornis* (adults [f], nymph) | | | | [64] |
| Wild Dogs (hybrid -- Dingo [*Canis lupus dingo*] x domestic dog [*Canis lupus familiaris*]) | *H. bancrofti* (adults) | Townsville, QLD<br>- fringing/suburban | Morphological | *Haemaphysalis* sp.: 33.3% (n = 9/27) | [70] |
| | *Haemaphysalis* sp. (larvae, nymphs) | | | | |
| | *Am. triguttatum* (adults) | | | *Amblyomma* sp.: 44.4% (n = 12/27) | |
| | *Amblyomma* sp. (larvae, nymphs) | | | | |
| Rabbit (*Oryctolagus cuniculus*) | *I. tasmani* (nymphs) | North Head, NSW | - Morphological<br>- Molecular (cPCR; PCR [amplify]; then NGS [Illumina])<br>[tick gDNA] | 100% (n = 7/7) | [51] |
| | *I. holocyclus* (nymphs, larvae) | | | | |
| | *I. trichosuri* (nymphs, larvae) | | | | |
| | *I. holocyclus* | Sydney Harbour National Park, NSW | - Morphological<br>- Molecular (PCR; then Sanger sequencing) | 90% (n = 38/42) | [37] |
| | *I. trichosuri* | | | | |
| | *I. hirsti* | | | | |
| | *I. tasmani* | | | | |
| | *Am. triguttatum* | Innes NP [Southern Yorke Peninsula], SA | Morphological | 62.5% (n = 5/8) | [59] |
| **Reptiles** | | | | | |
| Pygmy Bluetongue Skink (*Tiliqua adelaidensis*) | *B. hydrosauri* (larvae) | Burra, SA | Not reported | 12.5%<br>(n = 6/48) | [222] |
| | *B. hydrosauri* (adults [f]) | Burra, SA | Morphological | 0.33% (n = 1/300) | [29] |
| Shingleback Skinks [‘Sleepy Lizard’] (*Tiliqua [Trachydosaurus] rugosa*) | *Am. albolimbatum* | WA (Yanchep, Tammin, Southern Cross, Yellowdine, Wagin, Moulyinning, Esperance) | Morphological | Not reported | [44] |
| | *Am. triguttatum* (adult [f]) | Redbanks Conservation, SA | Not reported | 100% (n = 1/1) | [223] |
| | *B. hydrosauri* (adults [m+f], nymph, larvae) | Bundey Bore Station, SA | Not reported | Adult [f] – 32% (0.32)<br>Adult [m] -- 74% (0.74) | [43] |
| | *Am. limbatum* (adults [m+f]) | | | Adult [f] – 32% (0.32)<br>Adult [m] -- 61% (0.61) | |
| | *Am. vikirri* | Warruwarldunha Range [Hawker], SA | Not reported | 50% (n = 20/40) | [224] |
| | *Am. limbatum* | | | 35% (n = 14/40) | |
| | *Am. limbatum* (adults [m+f], nymphs) | Mt. Mary, SA | Not reported | 74.6%<br>(n = 135/181) | [225] |
| | *Ornithodoros gurneyi* | Bundey Bore Station, SA | Morphological | 2.5% (n = 14/552) | [45] |
| | *Am.* sp. *near limbatum* | Between Snowtown, Blyth, and Lochiel, SA | Morphological | Not reported | [65] |
| | *Aponomma [Bothriocroton] hydrosauri* | | | | |
| | *Am. triguttatum* (nymph) | Innes NP [Southern Yorke Peninsula], SA | Morphological | 3.3% (n = 1/30) | [59] |

| | | | | | |
|---|---|---|---|---|---|
| | *Aponomma* (*Bothriocroton*) *hydrosauri* | | | Not reported | |
| | *Ap.* (*Bothriocroton*) hydrosauri | Mt. Mary, SA | Not reported | Not reported | [226] |
| | *Am. limbatum* (adults [m+f], nymphs) | | | | |
| | *B. hydrosauri* | Bundey Bore Station, SA | Not reported | 70.3% (n = 109/155) | [227] |
| | *Am. albolimbatum* (adults [m+f], nymphs, larvae) | Eyre Peninsula, SA | - Morphological<br>- Molecular (NGS [DArT – SNPs; for larvae + nymphs]) | Not reported | [76] |
| | *Am. limbatum* (adults [m+f], nymphs) | | | | |
| | Hybrid (*Am.albo /Am.limb.*) (adults [m+f], nymph) | | | | |
| | *Am. albolimbatum* (adult [m+f]) | Port Gibbon [Eyre Peninsula], SA | Not reported | Not reported | [94] |
| | *Bothriocroton* (*Ap.*) *hydrosauri* (adult [m+f]) | | | | |
| | *Bothriocroton* (*Ap.*) hydrosauri (adults [m+f], nymphs, larvae) | Cavendish, VIC<br>SA (Port Lincoln, Pt. Parham, Swan Reach, Mt. Bold, Victor Harbour, Younghusband Peninsula) | Morphological | Not reported | [71] |
| | *B. hydrosauri* (adult [m+f], nymph, larvae) | Murray Mallee, SA | Not reported | Not reported | [155] |
| Blotched Blue-tongue Skink (*Tiliqua nigrolutea*) | *Aponomma sp* [Bothriocroton] | Flinders Island (Bass Strait), TAS | Not reported | Not reported | [148] |
| | *Am.* sp. *near limbatum* | Between Snowtown, Blyth, and Lochiel, SA | Morphological | Not reported | [65] |
| | *Aponomma* [*Bothriocroton*] *hydrosauri* | | | | |
| | *Ap.* (*Bothriocroton*) *hydrosauri* | Flinders Island, TAS | Morphological | Not reported | [149] |
| | *Bothriocroton* (*Ap.*) *hydrosauri* | TAS (Launceston, Hobart, Kelso, West Tamar, Green's Beach, Sandford) | Morphological | Not reported | [71] |
| | *Bothriocroton (Ap.) hydrosauri* | French Island National Park, VIC | Morphological | Not reported | [209] |
| Stokes' Skink (*Egernia stokesii*) | *Am. vikirri* | Warruwarldunha Range [Hawker], SA | Not reported | 94% (n = 30/32) | [224] |
| | *Am. vikirri* | Flinders Ranges, SA<br>Gawler Ranges, SA | Morphological | Not reported | [58] |
| Yellow-spotted monitor (*Varanus panoptes*) | *Am. fimbriatum* (adults [m+f]) | NT (Djukbinj National Park, Fogg Dam Nature Reserve, Humpty Doo) | Morphological | 81.8% (n = 27/33) | [46] |
| | *Am. moreliae* (adult [f]) | | | | |
| | *Am. fimbriatum* (adults [m+f]) | NT (Djukbinj National Park, Fogg Dam Nature Reserve) | Morphological | Not reported | [152] |
| | *Am. limbatum* (adults [m+f]) | | | | |
| Frilled-neck Lizard (*Chlamydosaurus kingii*) | *Am. limbatum* (adults [m+f]) | NT (Djukbinj National Park, Fogg Dam Nature Reserve, Humpty Doo) | Morphological | 66.7% (n = 4/6) | [46] |

| | | | | | |
|---|---|---|---|---|---|
| | *Amblyomma* sp. (nymph) | | | | |
| | *Am. fimbriatum* (adults [m+f]) | NT (Djukbinj National Park, Fogg Dam Nature Reserve) | Morphological | Not reported | [152] |
| | *Am. limbatum* (adults [m+f]) | | | | |
| | *Amblyomma* sp. (nymph) | | | | |
| Common Bluetongued skink (*Tiliqua scincoides*) | *Amblyomma* sp. (nymph) | NT (Djukbinj National Park, Fogg Dam Nature Reserve, Humpty Doo) | Morphological | Not reported | [152] |
| | *Am. moreliae* | Sydney, NSW | - Morphological<br>- Molecular (NGS (COI) [Illumina]) | Not reported | [86] |
| | *Am. moreliae* | Lismore, NSW<br>Canley Heights, NSW | - Morphological<br>- Molecular (cPCR [COI]; then Sanger sequencing) | Not reported | [84] |
| | *Bothriocroton* (*Ap.*) hydrosauri (adults [m+f], nymphs, larvae) | VIC (Melbourne, Cobungra Station) | Morphological | Not reported | [71] |
| Eastern Bearded Dragon *(Pogona barbata)* | *Aponomma* sp. (*Bothriocroton*) | Innes NP [Southern Yorke Peninsula], SA | Morphological | Not reported | [59] |
| Central Bearded Dragon (*Pogona* vitticeps) | *B. hydrosauri* | Blanchetown, SA | Morphological | Not reported | [71] |
| Lace monitor (*Varanus varius*) | *B. undatum* (adults [m+f], nymphs) | Burragorang Valley, NSW | Morphological | Not reported | [54] |
| | *B. undatum* (adult [f]) | Terrey Hills, NSW | - Morphological<br>- Molecular (cPCR [COI]) | Not reported | [81] |
| Perentie (*Varanus giganteus*) | *Am. calabyi* (adults [m+f]) | Mt. Conner (Curtain Springs), NT | Morphological | Not reported | [54] |
| | *Am. fimbriatum* (adults [m+f]) | | | | |
| | *Am. limbatum* (nymphs) | | | | |
| Spiny-tailed monitor (*Varanus acanthurus*) | *Am. limbatum* (adults [m+f]) | Fitzroy River (Kimberly), WA | Morphological | Not reported | [54] |
| Tiger Snake (*Notechis ater*) | *Aponomma sp* [*Bothriocroton*] | Flinders Island (Bass Strait), TAS | Not reported | Not reported | [148] |
| | *Ap.* (*Bothriocroton*) *hydrosauri* | Flinders Island, TAS | Morphological | Not reported | [149] |
| Eastern Tiger Snake (*Notechis scutatus*) | *B. hydrosauri* | TAS (Chappell Island [Bass Strait], Lower Langley) | Morphological | Not reported | [71] |
| Water Python (*Liasis fuscus*) | *Am. fimbriatum* (adults [m+f]) | NT (Djukbinj National Park, Fogg Dam Nature Reserve, Humpty Doo) | Morphological | 62.5% (n = 5/8) | [46] |
| | *Am. limbatum* (adults [m+f]) | | | | |
| | *Am. moreliae* (adults [m+f]) | | | | |
| | *Amblyomma* sp. (nymph) | | | | |
| | *Am. fimbriatum* (adults [m+f]) | NT (Djukbinj National Park, Fogg Dam Nature Reserve) | Morphological | Not reported | [152] |

| | | | | | |
|---|---|---|---|---|---|
| | *Am. limbatum* (adults [m+f]) | | | | |
| Green-tree Snake (*Dendrelaphis punctulatus*) | *Am. fimbriatum* (adults [m+f]) | NT (Djukbinj National Park, Fogg Dam Nature Reserve, Humpty Doo) | Morphological | 75% (n = 3/4) | [46] |
| | *Am. limbatum* (adults [m+f]) | | | | |
| | *Amblyomma* sp. (nymph) | | | | |
| | *Am. fimbriatum* (adults [m+f]) | NT (Djukbinj National Park, Fogg Dam Nature Reserve, Humpty Doo) | Morphological | Not reported | [152] |
| | *Am. limbatum* (adults [m+f]) | | | | |
| Copperhead Snake (*Austrelaps superbus*) | *Ap.* (*Bothriocroton*) *hydrosauri* | Flinders Island, TAS | Morphological | Not reported | [149] |
| | *B. hydrosauri* | TAS (Green's Beach, Kelso) | Morphological | Not reported | [71] |
| Diamond Python (*Morelia spilota spilota*) | *Am. moreliae* | Woronora Heights, NSW | - Morphological<br>- Molecular (cPCR [COI]; then Sanger sequencing) | Not reported | [84] |
| Red-Bellied Black Snake (*Pseudechis porphyriacus*) | *Am. moreliae* | Cronulla, NSW | - Morphological<br>- Molecular (cPCR [COI]; then Sanger sequencing) | Not reported | [84] |
| White-lipped Snake (*Drysdalia coronoides*) | *Bothriocroton* (*Ap.*) *hydrosauri* (adults [m+f], nymphs, larvae) | Green's Beach, TAS | Morphological | Not reported | [71] |
| Freshwater Crocodile (*Crocodylus johnstoni*) | *Amblyomma* sp. (nymph) | Lynd River, QLD | Morphological | 100% (n = 1/1) | [228] |
| **Birds** | | | | | |
| New Holland Honeyeater (*Phylidonyris novaehollandiae*) | *I. hirsti* | SA (Fleurieu Peninsula, Adelaide Hills, Kangaroo Island, York Peninsula) | Molecular (cPCR [COI + tRNA]) | 14.9% (n = 43/288) | [82] |
| | | SA (unspecified) | Morphological | 53.4% (n = 236/442) | [63] |
| | | Pelican Lagoon Research and Wildlife Centre, SA | Molecular (cPCR [COI + tRNA]) | 43.8% (n = 7/16) | [78] |
| | | Newland Head Conservation Park | | 40.5% (n = 15/37) | |
| | | SA (Pelican Lagoon Research and Wildlife Centre, Flinders Chase National Park, Newland Head Conservation Park) | Molecular (cPCR [COI + tRNA]) | Not reported | [79] |
| Emu (*Dromaius novaehollandiae*) | *I. holocyclus* (adults) | Central QLD (Fitzroy, Central West) | Morphological | Not reported | [55] |
| | *I. cornuatus* (adult [f]) | Healesville Sanctuary, VIC | Morphological | 100% (n = 1/1) | [229] |
| Kookaburra (unidentified) | *I. holocyclus* (adults) | Central QLD (Fitzroy, Central West) | Morphological | Not reported | [55] |
| Mixed Black Noddy (*Anous minutus*) | *O. capensis* (adults) | Central QLD (Heron Island) | Morphological | Not reported | [55] |
| Short-tailed shearwaters (*Ardenna tenuirostris*) | *I. eudyptidis* | Wedge Island, WA | Not reported | Not reported | [83] |
| | *I. kohlsi* | | | | |
| Little Penguins (*Eudyptula m. novaehollandiae*) | *I. eudyptidis* | - Phillip Island, VIC<br>- Montague Island, NSW<br>- SA (Kangaroo Island, Troubridge Island)<br>- WA (Garden Island, Wedge Island) | - Molecular (cPCR; then NGS [Illumina -COI]) | Not reported | [83] |
| | *I. kohlsi* | | | | |
| | *I. eudyptidis* | - Phillip Island, VIC<br>- Montague Island, NSW | Not reported | Not reported | [230] |
| | *I. kohlsi* | | | | |
| | *I. eudyptidis* | - NSW (Brush Island, Montague Island)<br>- Phillip Island, VIC | Morphological | Not reported | [231] |
| | *I. kohlsi* | | | | |

| | | | | | |
|---|---|---|---|---|---|
| | Hybrid (*I.eud/I.koh*) | | | | |
| | *I. kohlsi* | Phillip Island, VIC | - Morphological<br>- Molecular (cPCR [COI + 16S rRNA]; then Sanger sequencing)<br>[tick samples] | Not reported | [32] |
| Shy Albatross (*Thalassarche cauta*) | *I. eudyptidis* | Albatross Island, TAS | Not reported | Not reported | [111] |
| | *I. eudyptidis* | Albatross Island, TAS | Not reported | Not reported | [112] |
| Fairy Martin (*Petrochelidon ariel*) | *Ar. lagenoplastis* | Near Currawinya National Park, QLD | Morphological | Not reported | [56] |
| White-browed Scrubwren (*Sericornis frontalis*) | *I. hirsti* | SA (unspecified) | Morphological | 67.5% (n = 27/40) | [63] |
| | | Pelican Lagoon Research and Wildlife Centre, SA<br>Flinders Chase National Park, SA | Molecular (cPCR [COI + tRNA]) | Not reported | [79] |
| Striated Thornbill (*Acanthiza lineata*) | | SA (unspecified) | Morphological | 17.4% (n = 12/69) | [63] |
| | | Pelican Lagoon Research and Wildlife Centre, SA<br>Flinders Chase National Park, SA | Molecular (cPCR [COI + tRNA]) | Not reported | [79] |
| Brown Thornbill (*Acanthiza pusilla*) | | SA (unspecified) | Morphological | 26.1% (n = 12/46) | [63] |
| | | Flinders Chase National Park, SA | Molecular (cPCR [COI + tRNA]) | Not reported | [79] |
| Spotted Pardalote (*Pardalotus punctatus*) | | SA (unspecified) | Morphological | 36.4% (n = 4/11) | [63] |
| | | Newland Head Conservation Park, SA | Molecular (cPCR [COI + tRNA]) | Not reported | [79] |
| Striated Pardalote (*Pardalotus striatus*) | | SA (unspecified) | Morphological | 6.7% (n = 3/45) | [63] |
| Yellow-faced Honeyeater (*Lichenostomus chrysops*) | | SA (unspecified) | Morphological | 16.7% (n = 1/6) | [63] |
| Singing Honeyeater (*Lichenostomus virescens*) | | SA (unspecified) | Morphological | 85.7% (n = 6/7) | [63] |
| White-eared Honeyeater (*Lichenostomus leucotis*) | | SA (unspecified) | Morphological | 100% (n = 1/1) | [63] |
| Purple-gaped Honeyeater (*Lichenostomus cratitius*) | | SA (unspecified) | Morphological | 50% (n = 1/2) | [63] |
| Spiny-cheeked Honeyeater (*Acanthagenys rufogularis*) | | SA (unspecified) | Morphological | 100% (n = 1/1) | [63] |
| Little Wattlebird (*Anthochaera chrysoptera*) | | SA (unspecified) | Morphological | 100% (n = 2/2) | [63] |
| Red Wattlebird (*Anthochaera carunculata*) | | SA (unspecified) | Morphological | 14.3% (n = 1/7) | [63] |
| Tawny-crowned Honeyeater (*Glyciphila melanops*) | | SA (unspecified) | Morphological | 100% (n = 2/2) | [63] |
| | | Newland Head Conservation, SA | Molecular (cPCR [COI + tRNA]) | Not reported | [79] |
| Crescent Honeyeater (*Phylidonyris pyrrhopterus*) | | SA (unspecified) | Morphological | 28.1% (n = 18/64) | [63] |
| | | Flinders Chase National Park, SA | Molecular (cPCR [COI + tRNA]) | Not reported | [79] |
| Brown-headed Honeyeater (*Melithreptus brevirostris*) | | SA (unspecified) | Morphological | 35.3% (n = 6/17) | [63] |
| | | Pelican Lagoon Research and Wildlife Centre, SA | Molecular (cPCR [COI + tRNA]) | Not reported | [79] |
| Golden Whistler (*Pachycephala pectoralis*) | | SA (unspecified) | Morphological | 45.5% (n = 5/11) | [63] |

| | | | | | |
|---|---|---|---|---|---|
| Grey Fantail (*Rhipidura albiscapa*) | | SA (unspecified) | Morphological | 9.1% (n = 1/11) | [63] |
| Scarlet Robin (*Petroica boodang*) | | SA (unspecified) | Morphological | 13.6% (n = 3/22) | [63] |
| | | Pelican Lagoon Research and Wildlife Centre, SA | Molecular (cPCR [COI + tRNA]) | Not reported | [79] |
| Silvereye (*Zosterops lateralis*) | | SA (unspecified) | Morphological | 1.3% (n = 3/233) | [63] |
| Common Blackbird (*Turdus merula*) | | SA (unspecified) | Morphological | 66.7% (n = 2/3) | [63] |
| Masked lapwing (*Vanellus miles*) | *I. holocyclus* | Unspecified | Morphological | 19.2% (n = 10/52) | [38] |
| Australian wood duck (*Chenonetta jubata*) | | Unspecified | Morphological | 17.3% (n = 9/52) | [38] |
| Australian magpie (*Cracticus tibicen*) | | Unspecified | Morphological | 11.5% (n = 6/52) | [38] |
| Rainbow lorikeet (*Trichoglossus moluccanus*) | | Unspecified | Morphological | 11.5% (n = 6/52) | [38] |
| Tawny frogmouth (*Podargus strigoides*) | | Unspecified | Morphological | 7.7% (n = 4/52) | [38] |
| Common Koel (*Eudynamys scolopacea*) | | Unspecified | Morphological | 3.8% (n = 2/52) | [38] |
| Pale-headed rosella (*Platycercus adscitus*) | | Unspecified | Morphological | 3.8% (n = 2/52) | [38] |
| Brahminy kite (*Haliastur indus*) | | Unspecified | Morphological | 1.9% (n = 1/52) | [38] |
| Wandering whistling duck (*Dendrocygna arcuata*) | | Unspecified | Morphological | 1.9% (n = 1/52) | [38] |
| Collared sparrowhawk (*Accipiter cirrocephalus*) | | Unspecified | Morphological | 1.9% (n = 1/52) | [38] |
| Barn owl (*Tyto alba*) | | Unspecified | Morphological | 1.9% (n = 1/52) | [38] |
| White-cheeked rosella (*Platycercus eximius*) | | Unspecified | Morphological | 1.9% (n = 1/52) | [38] |
| Sulphur-crested cockatoo (*Cacatua galerita*) | | Unspecified | Morphological | 1.9% (n = 1/52) | [38] |
| Little corella (*Cacatua sanguinea*) | | Unspecified | Morphological | 1.9% (n = 1/52) | [38] |
| Purple swamp hen (*Porphyrio porphyrio*) | | Unspecified | Morphological | 1.9% (n = 1/52) | [38] |
| Bush stone curlew (*Burhinus grallarius*) | | Unspecified | Morphological | 1.9% (n = 1/52) | [38] |
| Laughing kookaburra (*Dacelo novaeguineae*) | | Unspecified | Morphological | 1.9% (n = 1/52) | [38] |
| Figbird (*Sphecotheres vieilloti*) | | Unspecified | Morphological | 1.9% (n = 1/52) | [38] |
| Noisy friarbird (*Philemon corniculatus*) | | Unspecified | Morphological | 1.9% (n = 1/52) | [38] |
| Southern boobook (*Ninox novaeseelandiae*) | | Unspecified | Morphological | 1.9% (n = 1/52) | [38] |
| Cattle Egret (*Bubulcus ibis coromandus*) | *Ar. robertsi* (adults, nymphs) | Lake Clarendon, QLD | Not reported | Not reported | [62] |
| | *Ar. robertsi* | Adelaide River, NT | Morphological | Not reported | [61] |

| | | | | | |
|---|---|---|---|---|---|
| Great Egret (*Egretta alba*) | | Adelaide River, NT | Morphological | Not reported | [61] |
| Little Egret (*Egretta garzetta*) | | Adelaide River, NT | Morphological | Not reported | [61] |
| Medium Egret (*Egretta intermedia*) | | Adelaide River, NT | Morphological | Not reported | [61] |
| Pied Heron (*Ardea picata*) | | Adelaide River, NT | Morphological | Not reported | [61] |
| Australian White Ibis (*Threskiornis molucca*) | | Adelaide River, NT | Morphological | Not reported | [61] |
| Little Black Cormorant (*Phalacrocorax sulcirostris*) | | Adelaide River, NT | Morphological | Not reported | [61] |
| Little Pied Cormorant (*Phalacrocorax melanoleucos*) | | Adelaide River, NT | Morphological | Not reported | [61] |
| Great Cormorant (*Phalacrocorax carbo novaehollandiae*) | | Lake Cowal, NSW | Morphological | Not reported | [61] |
| Galah (*Cacatua roseicapilla*) | | Lake Cowal, NSW | Morphological | Not reported | [61] |
| Silver Gull (*Larus* [*Chroicocephalus*] *novaehollandiae novaehollandiae*) | *I. laridis* (adult [m+f], nymph, larvae) | TAS (Wynyard, Tamar River)<br>Torquay, VIC | Morphological | Not reported | [48] |
| Australasian Gannet (*Morus serrator*) | *I. laridis* (adult [m+f], nymph, larvae) | Black Pyramid [Bass Strait], TAS<br>Phillip Island, VIC | Morphological | Not reported | [48] |
| "Albatross" (unspecified) | *I. laridis* (adult [m+f], nymph, larvae) | Albatross Island [Bass Strait], TAS | Morphological | Not reported | [48] |
| Tasmanian Masked Owl (*Tyto novaehollandiae castanops*) | *I. laridis* (adult [m+f], nymph, larvae) | TAS (Lemon Hill [Jericho], Campbell Town) | Morphological | Not reported | [48] |
| Australian Raven (*Corvus coronoides*) | *I. holocyclus* (adult [f]) | NSW (unspecified) | Morphological | Not reported | [39] |
| Australian Magpie (*Cracticus tibicen*) | *I. holocyclus* (adult [f]) | NSW (unspecified) | Morphological | Not reported | [39] |
| "Birds" (unspecified) | *I. hirsti* | SA [Fleurieu Peninsula] (Newland Head, Cox Scrub, Deep Creek, Flinders Chase, Vivonne Bay, Pelican Lagoon) | Not reported | Not reported | [113] |

**Abbreviations.** Life stages: m, male; f, female (e.g. adult [m+f] denotes adults of both sexes); nymph and larvae as stated.
States and territories: NSW, New South Wales; NT, Northern Territory; QLD, Queensland; SA, South Australia; TAS, Tasmania; VIC, Victoria; WA, Western Australia.
Identification methods: PCR, polymerase chain reaction; cPCR, conventional PCR; NGS, next-generation sequencing.
Genetic markers: COI, cytochrome c oxidase subunit I; 16S rRNA, 16S ribosomal RNA; 12S rRNA, 12S ribosomal RNA; tRNA, transfer RNA; gDNA, genomic DNA; DArT, Diversity Arrays Technology; SNP, single-nucleotide polymorphism.
Other terms: sp., species (singular); spp., species (plural); Am., Amblyomma; Ap., Aponomma; B., Bothriocroton; H., Haemaphysalis; I., Ixodes; O., Ornithodoros; Ar., Argas; NP, National Park; LGA, local government area; n, number.

**Supplementary Table S2.** Tick-borne microorganism studies in Australian wildlife, organised by host species and sample type

| Wildlife Host | Sample (tick, host tissue, or blood) | Location(s) | Pathogen(s) detected | Method(s) of Detection [+ sample type used] | Occurrence (% test-positive) | Reference |
|---|---|---|---|---|---|---|
| **Bacteria** | | | | | | |
| Koala | *Ixodes tasmani* (adult [m]) | Koala Conservation Centre [Phillip Island], VIC | *Bartonella*-like sp. (DNA) | - Molecular (cPCR, then Sanger sequencing [PCR products]) [tick samples] | 21% (cPCR) (n = 4/19) 75% (seq.) (n = 3/4) | [208] |
| | *I. tasmani* | - QLD (unspecified) - NSW (unspecified) | *Chlamydiales* spp. (*Ca.* Rhabdochlamydia) | - Molecular (cPCR; then Sanger sequencing) [tick samples] | Not reported | [34] |
| | | | *Chlamydiales* spp. *(Ca.* Fritschea) | | | |
| | | | *Chlamydiales* spp. *(Ca.* Rhabdochlamydia) | | | |
| | *I. holocyclus* | | *Chlamydiales* spp. *(Ca.* Fritschea*)* | | | |
| | | | *Chlamydiales* spp. (*Ca.* Rhabdochlamydia) | | | |
| | *I. tasmani* (adults [m]) | Phillip Island, VIC | *Rickettsiella* spp. | - Molecular (cPCR; then Sanger sequencing) - gel electrophoresis (PCR products) [tick samples] | 19.1% (n = 8/42) | [72] |
| | *I. tasmani* | Portland, VIC | *Rickettsia* sp. | - Molecular (microfluidic real-time PCR, cPCR; then Sanger sequencing) - + Molecular (qPCR) – *Rickettsia* sp. [tick samples] | 25% (n = 1/4) | [32] |
| | *I. tasmani* (adults [m+f]) | Port Macquarie, NSW | *Rickettsia* sp. | - Molecular (cPCR; then Sanger sequencing) - Gel electrophoresis (PCR products) | 28.3% (n = 22/78) | [157] |
| Echidna | *Bothriocroton concolor* (adults [m+f]) | Beerwah, QLD | *Borrelia* sp. | - Molecular (nPCR, cPCR, + forward and reverse nested PCR sequencing) - gel electrophoresis (PCR products) [tick samples] | 45.7 % (n = 37/81) | [137] |
| | | Wagga Wagga, NSW | | | 25% (n = 3/12) | |
| | *B. concolor* | NSW (unspecified) | *Anaplasma* sp. (OTU) | - Molecular (cPCR; + NGS [Illumina]) [tick samples] | 5% (n = 1/20) | [36] |
| | | | *Coxiella*-like sp. (OTU) | | 95% (n = 19/20) | |
| | | QLD (unspecified) | *Ca.* Borrelia. tachyglossi (OTU) | | 40% (n = 8/20) | |
| | *B. tachyglossi* (adults [f]) | Rockhampton, QLD | *Rickettsia* sp. | - Molecular (real-time PCR; + conventional hemi-nested PCR [+ve samples]; then Sanger sequencing) - gel electrophoresis (PCR products) [tick samples (midgut)] | Not reported | [55] |
| | | | *Rickettsia monacensis* | | | |
| | | | *Rickettsia* sp. | | | |
| | *B. concolor* | Bunyip, VIC | *Rickettsia bellii* | - Molecular (cPCR; then Sanger sequencing) | 75% (n = 3/4) | [72] |

| | | | | | | |
|---|---|---|---|---|---|---|
| | | | *Rickettsia* sp. | - gel electrophoresis (PCR products) [tick samples] | | |
| | *I. holocyclus* (adult [f]) | NSW (unspecified) | *Borrelia* sp. | Molecular (qPCR; then NGS [Ion Torrent]) | 100% (n = 1/1) | [39] |
| 'Possum' (unidentified) | *Haemaphysalis bremneri* | QLD (unspecified) | *Francisella* [endosymbiont] sp. (OTU) | - Molecular (cPCR; + NGS [Illumina]) [tick samples] | 50% (n = 2/4) | [36] |
| Brush-tailed Possum | Not reported | Kioloa, NSW | *Ehrlichia* sp. | - Molecular (NGS [Illumina]; + cPCR, qPCR [confirm detection], + Sanger sequencing (PCR products)) - gel electrophoresis (PCR products) [wildlife blood sample] | 33.3% (n = 1/3) | [31] |
| | Not reported | Flinders Island (Bass Strait), TAS | *Rickettsia australis* | - Serology (competitive ELISA assay) [antibodies] | 50% (n = 2/4) | [148] |
| | Blood | North Head, NSW | *Bartonella* spp. | - Molecular (NGS [Illumina]) - Molecular (nPCR [*Neoehrlichia* + *Ehrlichia*]; cPCR [*Bartonella*]; then Sanger sequencing) - gel electrophoresis (PCR products) [blood samples, tissue (skin/spleen) samples, tick samples] | 66.7% (n = 14/21) | [51] |
| | | Stapleton Reserve, NSW | | | 4.8% (n = 1/21) | |
| | Tissue | North Head, NSW | | | 45% (n = 9/20) | |
| | | Stapleton Reserve, NSW | | | 10% (n = 2/20) | |
| | *I. holocyclus* (nymphs) | Stapleton Reserve, NSW | *Ca.* Neoehrlichia arcana | | 66.7% (n = 2/3) | |
| | Tissue | North Head, NSW | | | 5% (n = 1/20) | |
| | Blood | Julimar State Forest, WA | | | 14.3% (n = 3/21) | |
| | Blood | North Head, NSW | *Ca.* Neoehrlichia australis | | 4.8% (n = 1/21) | |
| | *I. trichosuri* (adult [f]) | | | | 38.9%(n = 7/18) | |
| | *I. holocyclus* (adult [f]) | Stapleton Reserve, NSW | *Ehrlichia* sp. (nov. POS) | | 100% (n = 6/6) | |
| | Tissue | | | | 30% (n = 6/20) | |
| | Blood | | | | 14.3% (n = 3/21) | |
| | *Amblyomma triguttatum* (larvae) | Julimar State Forest, WA | *Rickettsiella* sp. | | 33.3% (n = 1/3) | |
| | *I. holocyclus* (nymph) | Dobroyd Head, NSW | | | 33.3% (n = 1/3) | |
| | *I. holocyclus* (nymph) | North Head, NSW | | | 33.3% (n = 1/3) | |
| | *I. tasmani* (adult [f]) | | | | 100% (n = 4/4) | |

| | | | | | | |
|---|---|---|---|---|---|---|
| | *I. trichosuri* (larvae, nymph, adult [f]) | | | | 22.2% (n = 4/18) | |
| | Tissue | | | | 5% (n = 1/20) | |
| | *I. tasmani* (adult [f]) | | *Coxiellaceae* | | 100%(n = 4/4) | |
| | Tissue | | | | 10% (n = 2/20) | |
| | | Stapleton Reserve, NSW | | | 5% (n = 1/20) | |
| | Blood | | | | 4.8% (n =1/21) | |
| | *Am. triguttatum* (larvae) | Julimar State Forest, WA | *Francisellaceae* | | 66.7% (n = 2/3) | |
| | | | *Francisella* sp. | | 33.3% (n = 1/3) | |
| | | | *Rickettsia* sp. | | 66.7% (n = 2/3) | |
| | Not reported | Northern QLD | *Coxiella burnetii* | - Molecular (real-time qPCR; then Sanger sequencing [com1 primers]) [blood, + tick samples]<br>- Serological (competitive ELISA) [sera] | Blood – 50% (n = 1/2) | [142] |
| | *I. tasmani* | QLD (unspecified) | - *Chlamydiales* spp. (*Ca. Rhabdochlamydia*)<br>- *Chlamydiales* spp. *(Ca. Fritschea*) | - Molecular (cPCR; then Sanger sequencing) [tick samples] | Not reported | [34] |
| | *I. holocyclus* | | | | | |
| Shingleback skink | *Am. albolimbatum* (adult [m+f], nymph) | Moulyinning, WA | *Rickettsia* sp. (DNA) | - Molecular (real-time qPCR; + cPCR (partial fragments); + sequencing [PCR amplicons]) [Tick samples] | 100% (n = 4/4) | [44] |
| | | Wagin, WA | | | 100% (n = 3/3) | |
| | | Yanchep, WA | | | 100% (n = 8/8) | |
| | | Esperance, WA | | | 100% (n = 2/2) | |
| | | Southern Cross, WA | | | 88.9% (n = 16/18) | |
| | | Yellowdine | | | 87.5% (n = 7/8) | |
| | | Tammin, WA | | | 83.3% (n = 5/6) | |
| | | Youndegin, WA | | | 100% (n = 3/3) | |
| | | Doodlakine, WA | | | 100% (n = 1/1) | |
| | | Korbel, WA | | | 90.9% (n = 10/11) | |
| | *B. hydrosauri* (adults [m+f], nymph, larva) | Murray Mallee, SA | *Rickettsia* sp. | Molecular (qPCR; then Sanger sequencing) [tick samples] | 100% (n = 41/41) | [155] |
| Long-nosed Bandicoot | *H. bancrofti* | NSW | *Anaplasma bovis*-like sp. (OTU) | Molecular (cPCR; + NGS [Illumina]) [tick samples] | 10% (n = 1/10) | [36] |
| | *H. humerosa* | | | | 10% (n = 1/10) | |
| | *I. holocyclus* | - NSW<br>- QLD | *Midichloria* sp. (OTU) | | 55.5% (n = 20/36) | |
| | *H. bancrofti* | NSW | | | 9% (n = 1/11) | |
| | *H. humerosa* | | | | 7.7% (n = 1/13) | |
| | *I. holocyclus* | - NSW | | | 41.6% (n = 15/36) | |

| | | | | | | |
|---|---|---|---|---|---|---|
| | | - QLD | | | | |
| | *H. bancrofti* | NSW | | | 9% (n = 1/11) | |
| | *I. tasmani* | - NSW<br>- QLD | *Ca.* Neoehrlichia arcana | | 7.8% (4/51) | |
| | *I. holocyclus* | | | | 5.5% (2/36) | |
| | *H. bancrofti* | | | | 9 % (n = 1/11) | |
| | *H. humerosa* | | | | 7.7 % (n = 1/13) | |
| | *I. holocyclus* | -NSW | *Ca.* Neoehrlichia australis | | 22.2% (n = 8/36) | |
| | *I. tasmani* | -QLD | | | 3.9% (n = 2/51) | |
| | *I. holocyclus* | NSW | *Rickettsiella*-like sp. (OTU) | | 2.7% (n =1/36) | |
| | Not reported | Sydney, NSW | *Ca.* Neoehrlichia arcana | - Molecular (NGS [Illumina]; + cPCR, qPCR [confirm detection], + Sanger sequencing (PCR products) )<br>- gel electrophoresis (PCR products)<br>[wildlife blood sample] | 22.2% (n = 4/18) | [31] |
| | Tissue | Nareen Reserve, NSW | *Ca.* Neoehrlichia australis | - Molecular (NGS [Illumina])<br>- Molecular (nPCR [*Neoehrlichia* + *Ehrlichia*]; cPCR [*Bartonella*]; then Sanger sequencing)<br>- gel electrophoresis (PCR products)<br>[blood samples, tissue (skin/spleen) samples, tick samples] | 9% (n = 4/44) | [51] |
| | Blood | | | | 4% (n =1/25) | |
| | Tissue | Turimetta Headland, NSW | | | 4.5% (n = 2/44) | |
| | Blood | | | | 4% (n = 1/25) | |
| | *I. trichosuri* (nymphs) | North Head, NSW | | | 100% (n = 2/2) | |
| | Tissue | | | | 9% (n = 4/44) | |
| | Tissue | Stapleton Reserve, NSW | | | 13.6% (n = 6/44) | |
| | Blood | | | | 20% (n = 5/25) | |
| | *I. holocyclus* (larvae, adult [m]) | | | | 21.4% (n = 3/14) | |
| | *I. holocyclus* (larvae, nymph, adult [f]) | | *Ca.* Neoehrlichia arcana | | 5.9% (n = 2/34) | |
| | | Warriewood Wetlands, NSW | | | 5.9% (n = 2/34) | |
| | | Nareen Reserve, NSW | | | 2.9% (n = 1/34) | |
| | Blood | | | | 4% (n =1/25) | |
| | Tissue | | | | 4.5% (n = 2/44) | |
| | Blood | Dobroyd Head, NSW | | | 8% (n = 2/25) | |
| | Blood | North Head, NSW | | | 48% (n = 12/25) | |
| | Tissue | | | | 43.2% (n = 19/44) | |
| | Blood | Stapleton Reserve, NSW | | | 88% (n = 22/25) | |
| | Tissue | | | | 13.6% (n = 6/44) | |
| | Blood | Turimetta Headland, NSW | | | 8% (n = 2/25) | |
| | Tissue | | | | 4.5% (n = 2/44) | |
| | Blood | Warriewood, Wetlands, NSW | | | 4% (n = 1/25) | |
| | Tissue | | | | 2.3% (n = 1/44) | |

| | | | | | | |
|---|---|---|---|---|---|---|
| | *I. trichosuri* (nymph) | North Head, NSW | *Bartonella* sp. | | 50% (n = 1/2) | |
| | Blood | | | | 48% (n =12/25) | |
| | Tissue | | | | 6.8% (n = 3/44) | |
| | *I. tasmani* (nymph) | Dobroyd Head, NSW | *Rickettsiella* sp. | | 33.3% (n = 4/12) | |
| | Tissue | | | | 4.5% (n = 2/44) | |
| | *I. holocyclus* (nymph) | Nareen Reserve, NSW | | | 20% (n =1/5) | |
| | *I. tasmani* (nymph) | | | | 16.7% (n = 2/12) | |
| | Tissue | | | | 4.6% (n =2/44) | |
| | *I. trichosuri* (larvae) | North Head, NSW | | | 33.3% (n = 1/3) | |
| | Tissue | | | | 2.3% (n = 1/44) | |
| | *I. tasmani* (larvae, nymphs) | Stapleton Reserve, NSW | | | 55% (n = 33/60) | |
| | Blood | | | | 24% (n = 6/25) | |
| | Tissue | | | | 34.1% (n = 15/44) | |
| | *I. holocyclus* (nymph) | Turimetta Headlands, NSW | | | 20% (n =1/5) | |
| | *I. tasmani* (nymph) | | | | 16.7% (n = 2/12) | |
| | Tissue | | | | 9.1% (n = 4/44) | |
| | *I. holocyclus* (nymph, adult [f]) | Warriewood Wetlands, NSW | | | 8% (n = 2/25) | |
| | *I. tasmani* (nymph) | | | | 58.3% (n = 7/12) | |
| | *I. trichosuri* (larvae) | | | | 33.3% (n =1/3) | |
| | Tissue | | | | 15.9% (n = 7/44) | |
| | Tissue | Condover Reserve, NSW | | | 11.4% (n = 5/44) | |
| | *I. tasmani* (nymph) | Dobroyd Head, NSW | *Coxiellaceae* | | 33.3% (n = 4/12) | |
| | Tissue | | | | 2.3% (n = 1/44) | |
| | *I. tasmani* (nymph) | Nareen Reserve, NSW | | | 16.7% (n = 2/12) | |
| | *I. holocyclus* (adult [f]) | Stapleton Reserve, NSW | | | 10% (n = 2/20) | |
| | *I. tasmani* (nymph) | | | | 38.3% (n = 23/60) | |
| | Tissue | | | | 2.3% (n = 1/44) | |
| | *I. tasmani* (nymph) | Turimetta Headland, NSW | | | 16.7% (n =2/12) | |
| | *I. tasmani* (nymph) | Warriewood Wetlands, NSW | | | 50% (n = 6/12) | |
| | Tissue | | | | 6.8% (n = 3/44) | |
| | *I. tasmani* (nymph) | Dobroyd Head, NSW | *Rickettsia* sp. | | 8.3% (n = 1/12) | |
| | *I. tasmani* (nymph) | Nareen Reserve, NSW | | | 8.3% (n = 1/12) | |

| | | | | | | |
|---|---|---|---|---|---|---|
| | *I. holocyclus* (nymph) | | | | 20% (n = 1/5) | |
| | *I. trichosuri* (larvae, nymph) | North Head, NSW | | | 60% (n = 3/5) | |
| | *I. tasmani* (nymph) | Stapleton Reserve, NSW | | | 8.3% (n = 1/12) | |
| | *I. holocyclus* (larvae, nymph) | | | | 28.6% (n = 4/14) | |
| | *I. holocyclus* (nymph) | Turimetta Headland, NSW | | | 20% (n = 1/5) | |
| | *I. tasmani* (nymph) | Warriewood Wetland, NSW | | | 8.3% (n = 1/12) | |
| | *I. holocyclus* (nymph) | | | | 20% (n = 1/5) | |
| | *I. tasmani* | QLD (unspecified) | *Chlamydiales* spp. *(Ca.* Fritschea) | - Molecular (cPCR; then Sanger sequencing) [tick samples] | Not reported | [34] |
| | *H. humerosa* | | | | | |
| Northern Brown Bandicoot | *H. humerosa* | QLD (unspecified) | *Ca.* Borrelia tachyglossi (OTU) | - Molecular (cPCR; + NGS [Illumina]) [tick samples] | 7.7% (n = 1/13) | [36] |
| | | - QLD (unspecified)<br>- NT (unspecified) | *Francisella* [endosymbiont] sp. | | 84.6% (n = 11/13) | |
| | Tick(s) unspecified | QLD (unspecified) | *Rickettsia* sp. (DNA) | - Molecular (real-time qPCR; then cPCR [ampl. of qPCR DNA]; then sequencing) [tick samples] | 16.7% (n = 3/18) | [75] |
| | *I. holocyclus* | Northern QLD (unspecified) | *Coxiella burnetii* | - Molecular (real-time qPCR; then Sanger sequencing [com1 primers]) [blood, + tick samples]<br>- Serological (competitive ELISA) [sera] | Blood – 17.1% (n = 6/35)<br>Tick – 33.3% (n = 10/30)<br>Serum -- 31.4% (n = 11/35) | [142] |
| Eastern-barred Bandicoot | *I. tasmani* | TAS (unspecified) | *Rickettsiella*-like sp. (OTU) | - Molecular (cPCR; + NGS [Illumina]) [tick samples] | Not reported | [36] |
| 'Bandicoot' | *H. humerosa* (adult [f]) | Port Douglas, QLD | *Rickettsia* sp. | - Molecular (real-time PCR; + conventional hemi-nested PCR [+ve samples]; then Sanger sequencing)<br>- gel electrophoresis (PCR products) [tick samples (midgut)] | 100% (n = 1/1) | [55] |
| | | | *Rickettsia* sp. | | | |
| | | | *Rickettsia* sp. | | | |
| Red-necked Wallaby | *H. bancrofti* | NSW (unspecified) | *Anaplasma bovis*-like sp. (OTU) | Molecular (cPCR; + NGS [Illumina]) [tick samples] | 10% (n = 1/10) | [36] |
| | | QLD (unspecified) | *Francisella* [endosymbiont] sp. | | 72.7% (n = 8/11) | |
| | *H. humerosa* | NSW (unspecified) | | | 7.7% (n = 1/13) | |
| | *I. fecialis* | | *Midichloria* sp. (OTU) | | 7.7 % (n = 1/13) | |
| | *I. holocyclus* | QLD (unspecified) | *Chlamydiales* spp. *(Ca.* Fritschea) | - Molecular (cPCR; then Sanger sequencing) [tick samples] | Not reported | [34] |
| | *H. bancrofti* | | | | | |
| | *H. longicornis* | | | | | |

| | | | | | | |
|---|---|---|---|---|---|---|
| Agile Wallaby | *Am. triguttatum* | Northern QLD (unspecified) | *Coxiella burnetii* | - Molecular (real-time qPCR; then Sanger sequencing [com1 primers]) [blood, + tick samples]<br>- Serological (competitive ELISA) [sera] | Blood – 20% (n = 1/5)<br>Sera – 60% (n = 3/5) | [142] |
| Black-striped Wallaby | Not reported (none) | Northern QLD (unspecified) | *Coxiella burnetii* | - Molecular (real-time qPCR; then Sanger sequencing [com1 primers]) [blood, + tick samples]<br>- Serological (competitive ELISA) [sera] | Blood – 100% (n = 1/1)<br>Serum – 100% (n = 1/1) | [142] |
| Quenda | *I. fecialis* | WA (unspecified) | *Bartonella bacilliformis* (OTU) | Molecular (cPCR; + NGS [Illumina]) [tick samples] | 100% (n = 2/2) | [36] |
| | | | *Ehrlichia* sp. (OTU) | | 7.7% (n = 1/13) | |
| | | | *Neoehrlichia* sp. (OTU) | | 15.4% (n = 2/13) | |
| | *I. australiensis* | | | | 7.7% (n = 2/26) | |
| | *Am. triguttatum* (nymph) | Whiteman Park, WA | *Francisellaceae* | - Molecular (NGS [Illumina])<br>- Molecular (nPCR [*Neoehrlichia* + *Ehrlichia*]; cPCR [*Bartonella*]; then Sanger sequencing)<br>- gel electrophoresis (PCR products)<br>[blood samples, tissue (skin/spleen) samples, tick samples] | 100% (n= 1/1) | [51] |
| | *I. australiensis* (nymph) | | | | 100% (n =1/1) | |
| Red Fox | *H. longicornis* | NSW (unspecified) | *Coxiella* sp. (OTU) | - Molecular (cPCR; + NGS [Illumina]) [tick samples] | 75% (n = 3/4) | [36] |
| Platypus | *I. ornithorhynchi* | QLD (unspecified) | *Ca. Ehrlichia ornithorhynchi* | - Molecular (cPCR; + NGS [Illumina]) [tick samples] | 60% (n = 6/10) | [36] |
| | *I. ornithorhynchi* | QLD (unspecified) | *Chlamydiales* spp. (*Ca. Rhabdochlamydia*) | - Molecular (cPCR; then Sanger sequencing) [tick samples] | Not reported | [34] |
| | | | *Chlamydiales* spp. *(Ca. Fritschea)* | | | |
| | *I. ornithorhynchi* | Nerum Creek, QLD | *Actinomyces* (rRNA) | - Molecular (NGS [Illumina]) [tick samples] | 10% (n = 1/10) | [197] |
| | | | *Corynebacterium* (rRNA) | | 50% (n = 5/10) | |
| | | | *Agromyces* (rRNA) | | 10% (n = 1/10) | |
| | | | *Leucobacter* (rRNA) | | 10% (n = 1/10) | |
| | | | *Kocuria* (rRNA) | | 10% (n = 1/10) | |
| | | | *Micrococcus* (rRNA) | | 30% (n = 3/10) | |
| | | | *Mycobacterium* (rRNA) | | 30% (n = 3/10) | |
| | | | *Propionibacterium* (rRNA) | | 60% (n = 6/10) | |
| | | | *Bacillus* (rRNA) | | 50% (n = 5/10) | |
| | | | *Lactobacillus* (rRNA) | | 10% (n = 1/10) | |

| | | | | | | |
|---|---|---|---|---|---|---|
| | | | *Stenotrophomonas* (rRNA) | | 30% (n = 3/10) | |
| | | | *Novosphingobium* (rRNA) | | 30% (n = 3/10) | |
| | | | *Delftia* (rRNA) | | 50% (n = 5/10) | |
| | | | *Rhodoferax* (rRNA) | | 10% (n = 1/10) | |
| | | | *Variovorax* (rRNA) | | 10% (n = 1/10) | |
| | | | *Ehrlichia* sp. | - Molecular (NGS [Illumina] [tick samples] | NGS: 70% (n = 7/10) Tick (PCR) -- 53.3% (n = 16/30) | |
| | | Inglis River, TAS | | - Molecular (cPCR; + nPCR/hemi-nested PCR; then Sanger sequencing) - gel electrophoresis (PCR products) [blood, + tick samples] - Morphological (light microscopy) [blood smear] | Blood – 11% (n = 3/27) Tick – 31% (n = 13/42) Smear -- 11% (n = 3/27) | |
| Red Kangaroo | *Am. triguttatum* | WA (unspecified) | *Francisella*-like [endosymbiont] sp. (OTU) | - Molecular (cPCR; + NGS [Illumina]) [tick samples] | 100% (n = 6/6) | [36] |
| | Not reported | Northern QLD (unspecified) | *Coxiella burnetii* | - Molecular (real-time qPCR; then Sanger sequencing [com1 primers]) [blood, + tick samples] - Serological (competitive ELISA) [sera] | Blood – 25% (n = 1/4) | [142] |
| Western Grey Kangaroo | *I. australiensis* | WA (unspecified) | *Rickettsiella*-like sp. (OTU) | - Molecular (cPCR; + NGS [Illumina]) [tick samples] | 3.84% (n = 1/26) | [36] |
| Eastern Grey Kangaroo | *Am. triguttatum* | Northern QLD (unspecified) | *Coxiella burnetii* | - Molecular (real-time qPCR; then Sanger sequencing [com1 primers]) [blood, + tick samples] - Serological (competitive ELISA) [sera] | Blood -- 35.3% (n = 6/17) Tick – 38.7% (n = 12/31) Serum -- 41.2% (n = 7/17) | [142] |
| | *H. bancrofti* | QLD (unspecified) | *Chlamydiales* spp. *(Ca. Fritschea)* | - Molecular (cPCR; then Sanger sequencing) [tick samples] | Not reported | [34] |
| Black Rat | Not reported | Flinders Island (Bass Strait), TAS | *Rickettsia australis* | - Serology (competitive ELISA assay) [antibodies] | 50% (n = 2/4) | [148] |
| | Tissue | North Head, NSW | *Ca.* Neoehrlichia australis | - Molecular (NGS [Illumina]) - Molecular (nPCR [*Neoehrlichia* + *Ehrlichia*]; cPCR [*Bartonella*]; then Sanger sequencing) - gel electrophoresis (PCR products) [blood samples, tissue (skin/spleen) samples, tick samples] | 1.3% (n = 1/80) | [51] |
| | | Warriewood Wetlands, NSW | | | 2.6% (n = 2/80) | |
| | Blood | North Head, NSW | *Ca.* Neoehrlichia arcana | | 2.4% (n =2/84) | |
| | *I. holocyclus* (larvae) | Stapleton Reserve, NSW | | | 16.7% (n = 2/12) | |
| | Blood | Turimetta Headlands, NSW | | | 1.2% (n =1/84) | |

| | | | | | | |
|---|---|---|---|---|---|---|
| | Blood | Warriewood Wetlands, NSW | | | 1.2% (n =1/84) | |
| | Tissue | | | | 3.8% (n = 3/80) | |
| | *I. tasmani* (nymph) | | *Neoehrlichia* sp. (nov.) | | 5.9% (n = 2/34) | |
| | Tissue | | | | 1.3% (n = 1/80) | |
| | Blood | Nareen Reserve, NSW | *Bartonella* sp. | | 1.2% (n = 1/84) | |
| | *I. tasmani* (nymph) | North Head, NSW | | | 2.9% (n = 1/34) | |
| | Blood | | | | 20.2% (n = 17/84) | |
| | Tissue | | | | 3.8% (n = 3/80) | |
| | Blood | Turimetta Headland, NSW | | | 2.4% (n = 2/84) | |
| | *I. tasmani* (larvae, nymph) | Warriewood Wetlands, NSW | | | 4.2% (n = 3/72) | |
| | *I. holocyclus* (larvae) | | | | 8.3% (n = 1/12) | |
| | Blood | | | | 31% (n = 26/84) | |
| | Tissue | | | | 5.2% (n = 4/80) | |
| | Tissue | Condover Reserve, NSW | *Borrelia* sp. (AUSBR) | | 5.2% (n = 4/80) | |
| | | Dobroyd Head | | | 6.3% (n = 5/80) | |
| | | Warriewood Wetlands | | | 11.3% (n = 9/80) | |
| | *I. tasmani* (larvae, nymph) | Condover Reserve, NSW | *Rickettsiella* sp. | | 29.2% (n = 21/72) | |
| | Blood | | | | 1.2% (n = 1/84) | |
| | Tissue | | | | 17.5% (n = 14/80) | |
| | *I. tasmani* (larvae, nymph) | Dobroyd Head, NSW | | | 16.7% (n = 12/72) | |
| | *I. holocyclus* (nymph) | | | | 20% (n = 1/5) | |
| | Tissue | | | | 3.8% (n = 3/80) | |
| | *I. tasmani* (nymph) | Manly Dam, NSW | | | 11.8% (n = 4/34) | |
| | Tissue | | | | 3.8% (n = 3/80) | |
| | *I. tasmani* (nymph) | Nareen Reserve, NSW | | | 11.8% (n = 4/34) | |
| | Tissue | | | | 1.3% (n = 1/80) | |
| | *I. tasmani* (larvae, nymph) | North Head, NSW | | | 34.7% (n = 25/72) | |
| | Tissue | | | | 10% (n = 8/80) | |
| | *I. tasmani* (nymph) | Stapleton Reserve, NSW | | | 11.8 % (n = 4/34) | |
| | Tissue | | | | 2.6% (n = 2/80) | |
| | *I. tasmani* (nymph) | Turimetta Headlands, NSW | | | 29.4% (n = 10/34) | |
| | Tissue | | | | 1.3% (n = 1/80) | |

| | | | | | | |
|---|---|---|---|---|---|---|
| | *I. tasmani* (larvae, nymph) | Warriewood Wetlands, NSW | | | 100% (n = 72/72) | |
| | *I. holocyclus* (nymph) | | | | 20% (n = 1/5) | |
| | Blood | | | | 8.3% (n = 7/84) | |
| | Tissue | | | | 25% (n = 20/80) | |
| | Tissue | Dobroyd Head, NSW | *Aquicella* sp. | | 1.3% (n = 1/80) | |
| | *I. tasmani* (larvae, nymph) | Condover Reserve, NSW | *Coxiellaceae* | | 26.4% (n = 19/72) | |
| | Tissue | | | | 1.3% (n = 1/80) | |
| | *I. tasmani* (larvae, nymph) | Dobroyd Head, NSW | | | 13.9% (n = 10/72) | |
| | *I. tasmani* (nymph) | Manly Dam, NSW | | | 11.8% (n = 4/34) | |
| | *I. tasmani* (nymph) | Nareen Reserve, NSW | | | 11.8% (n = 4/34) | |
| | *I. tasmani* (larvae, nymph) | North Head, NSW | | | 27.8% (n = 20/72) | |
| | Tissue | | | | 7.5% (n = 6/80) | |
| | *I. tasmani* (nymph) | Stapleton Reserve, NSW | | | 14.7% (n = 5/34) | |
| | *I. tasmani* (larvae, nymph) | Turimetta Headlands, NSW | | | 13.9% (n = 10/72) | |
| | *I. tasmani* (larvae, nymph) | Warriewood Wetlands, NSW | | | 96.2% (n = 76/79) | |
| | Tissue | | | | 2.5% (n = 2/80) | |
| | Blood | Dobroyd Head, NSW | *Mycoplasma* sp. | | 1.2% (n = 1/84) | |
| | Tissue | Stapleton Reserve, NSW | | | 1.3% (n = 1/80) | |
| | *I. tasmani* (nymph) | Nareen Reserve, NSW | *Rickettsia* sp. | | 5.9% (n = 2/34) | |
| | *I. tasmani* (nymph) | Stapleton Reserve, NSW | | | 2.9% (n = 1/34) | |
| | *I. holocyclus* (larvae) | | | | 16.7% (n = 2/12) | |
| | *I. tasmani* (larvae) | Turimetta Headland, NSW | | | 2.6% (n = 1/38) | |
| | *I. tasmani* (nymph) | Warriewood Wetland, NSW | | | 8.8% (n = 3/34) | |
| Bush Rat | Not reported | Gippsland, VIC | *Rickettsia australis* | - Serology (competitive ELISA assay) [antibodies] | 89.5% (n = 17/19) | [148] |
| | Blood | Manly Dam, NSW | *Bartonella* sp. | - Molecular (NGS [Illumina]) - Molecular (nPCR [*Neoehrlichia* + *Ehrlichia*]; cPCR [*Bartonella*]; then Sanger sequencing) - gel electrophoresis (PCR products) | 100% (n = 2/2) | [51] |
| | Tissue | | | | 66.7% (n = 2/3) | |
| | *I. tasmani* (larvae) | Warriewood Wetlands, NSW | | | 100% (n = 1/1) | |
| | Blood | | | | 100% (n = 2/2) | |
| | Tissue | | | | 33.3% (n = 1/3) | |

| | | | | | | |
|---|---|---|---|---|---|---|
| | Tissue | Warriewood Wetlands, NSW | *Borrelia* sp. (AUSBR) | [blood samples, tissue (skin/spleen) samples, tick samples] | 33.3% (n = 1/3) | |
| | *I. tasmani* (nymph) | Manly Dam, NSW | *Rickettsiella* sp. | | 66.7% (n = 2/3) | |
| | Tissue | | | | 33.3% (n = 1/3) | |
| | *I. tasmani* (larvae, nymph) | Warriewood Wetlands, NSW | | | 100% (n = 4/4) | |
| | Tissue | | | | 33.3% (n = 1/3) | |
| | *I. tasmani* (nymph) | Manly Dam, NSW | *Coxiellaceae* | | 66.7% (n = 2/3) | |
| | | Warriewood Wetlands, NSW | | | 66.7% (n = 2/3) | |
| | *I. tasmani* (larvae, nymph) | Warriewood Wetlands, NSW | *Rickettsia* sp. | | 75% (n = 3/4) | |
| | *Ixodes* sp. | WA (unspecified) | *Bartonella rattaustraliani* | - Molecular (nPCR, then single-step PCR, then Sanger sequencing)<br>- Gel electrophoresis (PCR products)<br>[tick samples] | 100% (n = 1/1) | [211] |
| Swamp Rat | Tissue | Warriewood Wetlands, NSW | *Borrelia* sp. (AUSBR) | - Molecular (NGS [Illumina])<br>- Molecular (nPCR [*Neoehrlichia* + *Ehrlichia*]; cPCR [*Bartonella*]; then Sanger sequencing)<br>- gel electrophoresis (PCR products)<br>[blood samples, tissue (skin/spleen) samples, tick samples] | 100% (n = 1/1) | [51] |
| | Blood | Nareen Reserve, NSW | *Mycoplasma* sp. | | 100% (n = 1/1) | |
| 'Rat' (unidentified) | Not reported | Flinders Island (Bass Strait), TAS | *Rickettsia australis* | - Serology (competitive ELISA assay)<br>[antibodies] | 50% (n = 2/4) | [148] |
| Tasmanian Devils | *I. tasmani* | TAS (unspecified) | *Rickettsiella*-like sp. (OTU) | - Molecular (cPCR; + NGS [Illumina])<br>[tick samples] | Not reported | [36] |
| | *I. tasmani* (adults [m+f], nymphs) | TAS (unspecified) | *Rickettsia* sp. (*Ca.* Rickettsia tasmanensis) | - Molecular (qPCR; + Sanger sequencing)<br>[tick samples] | 55% (n = 24/44) | [154] |
| | *I. tasmani* (adults [m+f], nymphs) | Wall Woolnorth, TAS | *Rickettsia* sp. | - Molecular (cPCR; then Sanger sequencing)<br>[tick samples]<br>- Gel electrophoresis (PCR products) | 30% (n = 3/10) | [72] |
| | | Surrey Hills, TAS | | | 66.7% (n = 10/15) | |
| | | Bronte Park, TAS | | | 28.6% (n = 4/14) | |
| | | Hastings, TAS | | | 100% (n = 2/2) | |
| | | Ida Bay, TAS | | | 50% (n = 1/2) | |
| Northern Bettong | Tick(s) unidentified | QLD (unspecified) | *Rickettsia* sp. (DNA) | - Molecular (real-time qPCR; then cPCR [ampl. of qPCR DNA]; then sequencing)<br>[tick samples] | 27.8% (n = 5/18) | [75] |
| Rufous Bettong | *Am. triguttatum* | Northern QLD (unspecified) | *Coxiella burnetii* | - Molecular (real-time qPCR; then Sanger sequencing [com1 primers])<br>[blood, + tick samples]<br>- Serological (competitive ELISA)<br>[sera] | Serum -- 100% (n = 1/1) | [142] |

| | | | | | | |
|---|---|---|---|---|---|---|
| Northern Quoll | Tick(s) unidentified | QLD (unspecified) | *Rickettsia* sp. (DNA) | - Molecular (real-time qPCR; then cPCR [ampl. of qPCR DNA]; then sequencing) [tick samples] | 20% (n = 1/5) | [75] |
| Spotted Quoll | *I. holocyclus* | NSW (unspecified) | *Chlamydiales* spp. *(Ca.* Fritschea) | - Molecular (cPCR; then Sanger sequencing) [tick samples] | Not reported | [34] |
| Western Quoll [Chuditch] | Blood | Avon Valley, WA | *Ehrlichia* sp. (nov CHU). | - Molecular (NGS [Illumina]) - Molecular (nPCR [*Neoehrlichia* + *Ehrlichia*]; cPCR [*Bartonella*]; then Sanger sequencing) - gel electrophoresis (PCR products) [blood samples, tissue (skin/spleen) samples, tick samples] | 16.7% (n = 4/24) | [51] |
| | Blood | Julimar State Forest, WA | | | 20.8% (n = 5/24) | |
| | Tissue | | | | 11.1% (n = 1/9) | |
| | Blood | Avon Valley | *Ca.* Neoehrlichia arcana | | 4.2% (n = 1/24) | |
| | *Am. triguttatum* (larvae) | Avon Valley, WA | *Rickettsia* spp. | | 100% (n = 1/1) | |
| Bare-nosed wombat | Not reported | Flinders Island (Bass Strait), TAS | *Rickettsia australis* | - Serology (competitive ELISA assay) [antibodies] | 100% (n = 1/1) | [148] |
| | *I. tasmani* | Not reported | *Chlamydiales* spp. *(Ca. Fritschea)* | - Molecular (cPCR; then Sanger sequencing) [tick samples] | Not reported | [34] |
| | *B. auruginans* (adult [f]) | Buxton, VIC | *Rickettsia* sp. (+ DNA) -*R. massiliae* | - Molecular (cPCR; then Sanger sequencing) - gel electrophoresis (PCR products) [tick samples] | 70% (n = 7/10) | [72] |
| | | | *Coxiella* sp. (+ DNA) | | 100% (n = 10/10) | |
| | *B. auruginans* (adults [m+f], nymphs) | NSW (Coolagilite, Rock Flat, Yowrie, Bellmount Forest, Bilpin, Bemboka, Buxton, Primrose Valley, Courijah, Orangville, Bungendore, Burra, Gelstone Park, Dalgety, Kangaroo Valley, Mowbray Park, Pheasant Nest, Picton, Quaama, The Oaks, Mount Hunter, The Rock, Wagga Wagga, Thirlmere, Wolgan Valley) [unspecified] | *Coxiella burnetii* | - Molecular (NGS [Illumina]) [tick samples] | 71.8 % (n = 56/78) | [50] |
| | | | *Staphylococcus sciuri* | | 20.5% (n = 16/78) | |
| | | | *Corynebacterium amycolatum* | | 11.5% (n = 9/78) | |
| | | | *Dermacoccus nishinomiyaensis* | | 15.4% (n = 12/78) | |
| | | | *Macrococcus brunensis* | | 25.6% (n = 20/78) | |
| | | | *Planomicrobium glaciei* | | 5.1% (n = 4/78) | |
| | | | *Lysinibacillus* sp. | | 10.3% (n = 8/78) | |
| | | | *Brachybacterium paraconglomeratum* | | 16.7% (n = 13/78) | |
| | | | *Escherichia coli* | | 20.5% (n = 16/78) | |
| | | | *Acinetobacter* sp. | | 24.4% (n = 19/78) | |
| | | | *Pseudomonas* sp. | | 9% (n = 7/78) | |
| | | | *Ca.* Borrelia ivorensis | | 1.3% (n = 1/78) | |
| | | | *Anaplasma* sp. | | 5.1% (n = 4/78) | |
| | *I. tasmani* (adult [f]) | NSW (unspecified) | *Rickettsiella* sp. | | 100% (n = 1/1) | |
| | *Aponomma. (Both.) auruginans* | Wilson's Promontory | *Coxiella*-like sp. | - Molecular (microfluidic real-time PCR, cPCR; then Sanger sequencing) | 100% (n = 2/2) | [32] |
| | | | *Rickettsia* sp. | | 50% (n = 1/2) | |

| | | | | | | |
|---|---|---|---|---|---|---|
| Swamp Antechinus | Not reported | Gippsland, VIC | *Rickettsia australis* | - Serology (competitive ELISA assay) [antibodies] | 100% (n = 1/1) | [148] |
| Mixed Black Noddy | *Ornithodoros capensis* (adults [f]) | Heron Island, QLD | *Coxiella*-like sp. | - Molecular (real-time PCR; + conventional hemi-nested PCR [+ve samples]; then Sanger sequencing)<br>- gel electrophoresis (PCR products)<br>[tick samples (midgut)] | Not reported | [55] |
| | | | *Rickettsia* sp. | | | |
| 'Kookaburra' | *I. holocyclus* (adult [f]) | Byfield, QLD | *Coxiella burnetii* | - Molecular (real-time PCR; + conventional hemi-nested PCR [+ve samples]; then Sanger sequencing)<br>- gel electrophoresis (PCR products)<br>[tick samples (midgut)] | 100% (n = 1/1) | [55] |
| Brown Antechinus | *I. antechini* (adult [f]) | Warriewood Wetlands, NSW | *Neoehrlichia* sp. (nov.) | - Molecular (NGS [Illumina])<br>- Molecular (nPCR [*Neoehrlichia* + *Ehrlichia*]; cPCR [*Bartonella*]; then Sanger sequencing)<br>- gel electrophoresis (PCR products)<br>[blood samples, tissue (skin/spleen) samples, tick samples] | 100% (n = 1/1) | [51] |
| | Tissue | | *Bartonella* sp. | | 50% (n = 1/2) | |
| | *I. tasmani* (larvae, nymph) | | *Rickettsiella* sp. | | 100% (n = 4/4) | |
| | | | *Coxiellaceae* | | 100% (n = 4/4) | |
| Rabbit | *I. tasmani* (nymph) | North Head, NSW | *Rickettsiella* sp. | - Molecular (NGS [Illumina])<br>- Molecular (nPCR [*Neoehrlichia* + *Ehrlichia*]; cPCR [*Bartonella*]; then Sanger sequencing)<br>- gel electrophoresis (PCR products)<br>[blood samples, tissue (skin/spleen) samples, tick samples] | 100% (n = 2/2) | [51] |
| | | | *Coxiellaceae* | | 100% (n = 2/2) | |
| | | | *Rickettsia* sp. | | 50% (n =1/2) | |
| Common Wallaroo | *Am. triguttatum* | Northern QLD (unspecified) | *Coxiella burnetii* | - Molecular (real-time qPCR; then Sanger sequencing [com1 primers])<br>[blood, + tick samples]<br>- Serological (competitive ELISA)<br>[sera] | Blood – 33.3% (n = 1/3) | [142] |
| Fairy Martin | *Argas lagenoplastis* | Near Currawinya National Park, QLD | *Rickettsia fournieri* | - Morphological (cell culture [XTC-2]; electron microscopy)<br>- Molecular (qPCR; then NGS [Illumina])<br>[tick samples] | Not reported | [56] |
| Brush-tailed Bettong ['Woylie'] | *I. australiensis* | WA (unspecified) | *Bartonella* sp. *(Ca.* B. woyliei) DNA | - Molecular (nPCR; + cPCR; then Sanger sequencing [ABI Prism])<br>- gel electrophoresis (PCR products)<br>[tick samples] | 1.9% (n = 1/53) | [211] |
| | | | *Bartonella* sp. DNA | | | |
| Yellow-spotted monitor | *Am. fimbriatum* (adults [m+f]) | NT (Djukbinj National Park, Fogg Dam Nature Reserve) | *Rickettsia* sp. (DNA) | - Molecular (cPCR; then Sanger sequencing)<br>- gel electrophoresis (PCR products)<br>[tick samples] | 32.9% (n = 48/148) | [152] |
| Water Python | | | *Rickettsia* sp. (DNA) | - Molecular (cPCR; then Sanger sequencing) | 53.3% (n = 8/15) | [152] |

| | | | | | | |
|---|---|---|---|---|---|---|
| | *Am. fimbriatum* (adults [m+f]) | NT (Djukbinj National Park, Fogg Dam Nature Reserve) | | - gel electrophoresis (PCR products) [tick samples] | | |
| | | | *Rickettsia* sp. | | 22.2% (n = 2/9) | |
| | | | *Francisella* sp. | | | |
| Green-tree Snake | *Am. fimbriatum* (adults [m+f]) | NT (Djukbinj National Park, Fogg Dam Nature Reserve) | *Rickettsia* sp. (DNA) | - Molecular (cPCR; then Sanger sequencing)<br>- gel electrophoresis (PCR products)<br>[tick samples] | 33.3% (n = 1/3) | [152] |
| Wild (feral) Pig | *Am. triguttatum* | Mundaring, WA | *Rickettsia* sp. (nov. BWI-1) | - Molecular (cPCR; then Sanger sequencing)<br>- gel electrophoresis (PCR products)<br>[tick samples] | 77.8% (n = 21/27) | [153] |
| | | Serpentine, WA | | | 78% (n = 46/59) | |
| | | Dwellingup, WA | | | 100% (n = 5/5) | |
| | | Not reported | *Rickettsia* sp. (antibodies) | - Serology (micro-immunofluroescence)<br>[blood] | 50% (n = 20/40) | |
| Host(s) unspecified:<br>- Copperhead snake<br>- Tiger snakes<br>- Blue-tongue lizard | *Ap.* (*Bothriocroton*) *hydrosauri* | Flinders Island, TAS | *Rickettsia honei* | - Molecular (cPCR)<br>[blood + tick haemolymph, egg pool]<br>- Morphological (electron microscopy, cell culture [Vero])<br>[tick samples] | Haemolymph – 63% (n = 29/46)<br>Egg pools – 100% (n = 2/2) | [149] |
| Western-Barred Bandicoot | *H. humerosa* (adult [m]) | Bernier Island, WA | *Coxiella burnetii* (+ antibodies) | - Molecular (qPCR; then Sanger sequencing)<br>[tick, host faeces]<br>- Serology (ELISA) | Tick -- 10% (n = 1/10) | [60] |
| | Not reported | Dorre Island, WA | | | Faeces – 8.3% (n = 1/12)<br>Serum – 14.3% (5/35) | |
| Mountain Brushtail Possum | *I. trichosuri* | Boho South, VIC | *Bartonella* sp. | - Molecular (microfluidic real-time PCR, cPCR; then Sanger sequencing)<br>- Molecular (qPCR) -- *Rickettsia* | 30% (n = 3/10) | [32] |
| | | | *Coxiella*-like sp. | | 10% (n = 1/10) | |
| | | | *Rickettsia* sp. | | 60% (n = 6/10) | |
| Agile Antechinus | *I. antechini* | Boho South VIC | *Bartonella* sp. | - Molecular (microfluidic real-time PCR, cPCR; then Sanger sequencing)<br>- Molecular (qPCR) -- *Rickettsia* | 75% (n = 3/4) | [32] |
| | | | *Ehrlichia* sp. | | 25% (n = 1/4) | |
| | | | *Rickettsia* sp. | | 100% (n = 4/4) | |
| Southern Brown Bandicoot | *I. tasmani* | Koo Wee Rup, VIC | *Bartonella* sp. | - Molecular (microfluidic real-time PCR, cPCR; then Sanger sequencing)<br>- Molecular (qPCR) -- *Rickettsia* | 25% (n = 2/8) | [32] |
| | | | *Rickettsia* sp. | | 100% (n = 8/8) | |
| | *I. trichosuri* | VIC (unspecified) | *Rickettsia* sp. | | 50% (n = 4/8) | |
| Eastern Blue-tongued Lizard<br>Diamond Python<br>Red-Bellied Black Snake | *Am. moreliae* | NSW (unspecified) | *Rickettsia* sp. | - Molecular (real-time qPCR; + conventional nPCR; then Sanger sequencing)<br>[tick samples] | 87.5% (n = 14/16) | [84] |
| Yellow-footed Antechinus | *I. antechini* | Dwellingup, WA | *Ca.* Bartonella antechini | Molecular (nPCR; + cPCR; then Sanger sequencing)<br>[tick samples] | 14.8% (n = 4/27) | [217] |

| | | | | | | |
|---|---|---|---|---|---|---|
| Lace Monitor | *B. undatum* (adults [m+f]) | Burragorang Valley, NSW | *Ca.* Borrelia undatum | - Molecular (nPCR, then Sanger sequencing and NGS [shotgun – Illumina])<br>- Gel electrophoresis (PCR products) | 6% (n = 5/84) | [54] |
| | *B. undatum* (adult [f]) | Terrey Hills, NSW | *Rickettsia* c.f. *tamurae* | - Molecular (NGS; then nPCR [*Borrelia, Rickettsia*], or cPCR [*C. burnetii*])<br>- Gel electrophoresis (PCR products)<br>[tick samples] | 100% (n = 6/6) | [81] |
| | | | Proteobacteria (OTU) | | 100% (n = 6/6) | |
| | | | Actinobacteria (OTU) | | 100% (n = 6/6) | |
| | | | Firmicutes (OTU) | | 100% (n = 6/6) | |
| | | | *Borrelia* sp. Tick3 | | 12% (n = 3/25) | |
| | | | *Borrelia* sp. Tick3/Tick14 | | | |
| Perentie | *Am. calabyi* (adults [m+f]) | Mt. Conner (Curtain Springs), NT | *Ca.* Borrelia rubricentralis | - Molecular (nPCR, then Sanger sequencing and NGS [shotgun – Illumina])<br>- Gel electrophoresis (PCR products) | 35.6% (n = 16/45) | [54] |
| | *Am. limbatum* (nymphs) | | *Borrelia* sp. | | 22.2% (n = 4/18) | |
| | *Am. fimbriatum* (adults [m+f]) | | | | 76.9% (n = 20/26) | |
| Rusa Deer | Tissue | Conondale, QLD | *Anaplasma platys* | - Molecular (NGS [Illumina])<br>[tissue (skin/spleen) samples] | 50% (n = 3/6) | [51] |
| | | | | | 16.7% (n = 1/6) | |
| Gould's Wattled Bat | *Ar. dewae* | Organ Pipes National Park, VIC | *R. japonica str. argasii* | Molecular (qPCR, then Sanger sequencing)<br>[tick samples] | 70% (n = 7/10) | [57] |
| *Vespadelus* sp. | *Ar. dewae* | Organ Pipes National Park, VIC | *R. japonica str. argasii* | Molecular (qPCR, then Sanger sequencing)<br>[tick samples] | 70% (n = 7/10) | [57] |
| Not reported (i.e. collected off a wildlife host) | Tick(s) unspecified:<br>- *B. concolor*<br>- *H. bancrofti*<br>- *H. humerosa*<br>- *I. antechini*<br>- *I. australiensis*<br>- *I. holocyclus*<br>- *I. tasmani* | - NSW<br>- NT<br>- QLD<br>- WA<br>[unspecified] | *Rickettsia* sp. (OTU) | - Molecular (cPCR; + NGS [Illumina])<br>[tick samples] | Not reported | [36] |
| | *I. australiensis* | Not reported | *Rickettsiella*-like sp. (OTU) | - Molecular (cPCR; + NGS [Illumina])<br>[tick samples] | 84.6% (n = 22/26) | [36] |
| | *I. holocyclus* (adult [f]) | NSW (unspecified) | *Rickettsia* sp. | Molecular (qPCR; then NGS [Ion Torrent]) | Not reported | [39] |
| | | | *Anaplasma* sp. | | | |
| | | | *Leptospira inadai* | | | |
| | | | *Clostridium histolyticum* | | | |
| | | | *Ca.* Neoehrlichia arcana | | | |
| | | | *Ca.* Neoehrlichia australis | | | |
| Questing Ticks (collected in | *H. bancrofti* | Kioloa, NSW | - *Francisella* sp.[endosymbiont] | - Molecular (NGS [Illumina]; + cPCR, qPCR [confirm detection], + Sanger sequencing (PCR products)) | 100% (n = 31/31) | [31] |
| | | Sydney, NSW | | | 100% (n = 1/1) | |

| | | | | | | |
|---|---|---|---|---|---|---|
| same area as wildlife:<br>- Black Rat<br>- Bush Rat<br>- Brush-tailed possum<br>- Long-nosed Bandicoot) | | | - *Rickettsia* sp. [endosymbionts] | - gel electrophoresis (PCR products)<br>[tick samples] | | |
| | | Kioloa, NSW | *Borrelia* sp. nov. HB | | 6.5% (n = 2/31) | |
| | | Kioloa, NSW | *Anaplasma bovis* | | 9.7% (n = 3/31) | |
| | *I. holocyclus* | Kioloa, NSW | *Ca.* Midichloria sp. | | 100% (n = 99/99) | |
| | | Sydney, NSW | | | 100% (n = 46/46) | |
| | | Kioloa, NSW | *Rickettsia australis* | | 3% (n = 3/99) | |
| | | Sydney, NSW | | | 2.2% (n = 1/46) | |
| | | Kioloa, NSW | *Ca.* Neoehrlichia australis | | 29.3% (n= 29/99) | |
| | | Sydney, NSW | | | 26.1% (12/46) | |
| | | Kioloa, NSW | *Ca.* Neoehrlichia arcana | | 24.2% (24/99) | |
| | | Sydney, NSW | | | 30.4% (n = 14/46) | |
| Questing Tick | *I. holocyclus* (adult [f]) | NSW (unspecified) | *Francisella* sp. | Molecular (qPCR; then NGS [Ion Torrent]) | Not reported | [39] |
| **Protozoa** | | | | | | |
| Platypus | *I. ornithorhynchi* | Inglis Catchment, TAS | *Theileria* sp. | - Morphological (light microscopy)<br>[Platypus blood] | - *Theileria* sp. -- 92.3% (n = 131/142) | [178] |
| | | | *Trypanosoma* spp. | | - *Trypanosomes* – 91.6 % (n = 131/143) | |
| | *I. ornithorhynchi* | NSW (Abercrombie River, Upper Murrumbidgee River, Queanbeyan River) | *Theileria ornithorhynchi* | - Morphological (light microscopy) [Blood sample] | 100% (n = 4/4) | [97] |
| | Not reported | Not reported | *Theileria ornithorhynchi* | - Molecular (NGS [18s rDNA – GS-FLX]; then PCR then DNA sequencing)<br>[blood samples]<br>- Morphological (light microscopy)<br>[smear]<br><br>- Gel electrophoresis (PCR products) | 100% (n = 3/3) | [175] |
| Echidna | Not reported | Not reported | *Theileria tachyglossus* | - Molecular (NGS [18s rDNA – GS-FLX]; then PCR then DNA sequencing)<br>[blood samples]<br>- Morphological (light microscopy)<br>[smear]<br><br>- Gel electrophoresis (PCR products) | 66.7% (n = 4/6) | [175] |
| Western Grey Kangaroo | *I. australiensis* (nymph, adult [F]) | Lowlands, WA | *Theileria* sp. | - Molecular (nPCR, + Sanger sequencing [PCR products])<br>- gel electrophoresis (PCR products) | 7.6% (n = 9/119) | [179] |
| | | Albany, WA | | | | |

| | | | | | | |
|---|---|---|---|---|---|---|
| | | | | [tick samples] | | |
| | Not reported | Boyup Brook, WA | *Theileria fuliginosa* | - Molecular (cPCR)<br>- Morphological (light microscopy)<br>[blood samples] | 100% (n = 1/1) | [180] |
| Eastern Grey Kangaroo | *Haemaphysalis* sp. | - Fordsdale, QLD<br>- Bingie, NSW | *Babesia* sp.<br>(*Babesia macropus*) | - Molecular (cPCR, + Sanger sequencing [PCR products])<br>- gel electrophoresis (PCR products)<br>- Morphological (light [blood] + electron microscopy [kidney]) | 100% (n = 3/3)<br><br>[EG Kangaroo blood + kidney tissue sample [n = 1]] | [164] |
| | *I. holocyclus* (adults [f], nymph) | Mid-North Coast, NSW (unspecified) | *Piroplasma rRNA* | - Molecular (qPCR [x2]; touchdown PCR; and then Sanger sequencing)<br>- gel electrophoresis (PCR products) | Nymphs – (n = 3/54)<br>Adult [f] – (n= 1/54) | [73] |
| | *Ixodes* sp. (nymph) | | | | 1.9% (n = 1/54) | |
| | *Haemaphysalis* sp.(larvae) | | | | 6.45% (n = 10/155) | |
| | *H. petrogalis* (adult [m]) | | | | 1.3% (n = 2/155) | |
| | Not reported | South-east QLD [coastline] (unspecified) | *Babesia macropus* | - Morphological (histopathology; + cytology [impression smear]; + blood – light microscopy; electron microscopy)<br>[tissue samples: kidney, liver, brain, spleen, bone marrow]<br>- Molecular (cPCR, then Sanger sequencing)<br>[tissue samples: kidney, liver, brain, spleen, bone marrow] | Histopathology: 100% (n = 31/31)<br>Blood: 43.8% (n = 14/32)<br>PCR: 100% (n = 16/16) | [165] |
| | | North-east NSW [coastline] (unspecified) | | | | |
| | Not reported | Tidbinbilla Reserve, ACT | *Theileria* sp. | - Molecular (cPCR, + nPCR; then Sanger sequencing)<br>- gel electrophoresis (PCR products)<br>- Morphological (light microscopy) | 100% (n = 1/1) | [174] |
| Long-nosed Bandicoot | Not reported | Sydney, NSW | *Theileria* cf. *peramelis* | - Molecular (NGS [Illumina]; + cPCR, qPCR [confirm detection], + Sanger sequencing (PCR products) )<br>- gel electrophoresis (PCR products)<br>[wildlife blood sample] | 90.1% (n = 10/11) | [31] |
| | Not reported | Not reported | *Trypanosoma gilletti* | - Molecular (NGS [Illumina]; + semi-nPCR, + cPCR [ampl. of nPCR products]) -- *Trypanosomes*<br>[tissue (skin/spleen) samples, blood samples]<br>- Morphological (light microscopy)<br>[blood] | Not reported | [125] |
| | | | *Theileria* c.f. *peramelis* | | | |
| | *I. tasmani* (nymphs) | Beerwah, QLD | *Theileria* sp. | - Molecular (nPCR; then Sanger sequencing)<br>- gel electrophoresis (PCR products) | 45.5% (n = 5/11) | [64] |
| | | | *Theileria* sp. | | (n = 5/137) | |

| | | | | | | |
|---|---|---|---|---|---|---|
| | | | | [tick samples] | | |
| Northern Brown Bandicoot | Not reported | Darwin, NT | *Trypanosoma vegrandis* | Molecular (nPCR, cPCR [*Hepatozoon*]; then Sanger sequencing)<br><br>Morphological (light microscopy) – *Hepatozoon*<br><br>[blood samples] | 26.5% (n = 30/113) | [166] |
| | | Kakadu National Park NT | | | | |
| | | Bathurst Island, NT | | | | |
| | | Groote Eylandt, NT | | | | |
| | | Cobourg Peninsula, NT | | | | |
| | | | *Trypanosoma* sp. (NTB84) | | | |
| | | Darwin, NT | *Hepatozoon* sp. | | PCR -- 9.7% (n = 11/113)<br><br>Blood smear – 18.2% (n = 2/11) | |
| | | Kakadu National Park, NT | | | | |
| | | Bathurst Island, NT | | | | |
| | | Cobourg Peninsula, NT | | | | |
| | | Darwin, NT | *Babesia* sp. | | 9.7% (n = 11/113) | |
| | | Bathurst Island, NT | | | | |
| | | Cobourg Peninsula, NT | | | | |
| Brush-tailed Possum | Not reported | Kioloa, NSW | *Babesia lohae* | - Molecular (NGS [Illumina]; + cPCR, qPCR [confirm detection], + Sanger sequencing (PCR products) )<br>- gel electrophoresis (PCR products)<br>[wildlife blood sample] | 33.3% (n = 1/3) | [31] |
| | | Sydney, NSW | | | 100% (n = 3/3) | |
| | Not reported | Not reported (either near Perth or near Sydney) | *Trypanosoma gilletti* | - Molecular (NGS [Illumina]; + semi-nPCR, + cPCR [ampl. of nPCR products]) -- *Trypanosomes*<br>[tissue (skin/spleen) samples, blood samples] | Not reported | [125] |
| | | | *Trypanosoma cyclops*-like (sequence) | | 33.3% (n = 6/18)<br>[blood = 5, tissue =1] | |
| | | WA (near Perth) | *Trypanosoma noyesi* | - Molecular (NGS [Illumina]; + semi-nPCR, + cPCR [ampl. of nPCR products]) -- *Trypanosomes*<br>[blood samples] | 5.6% (n = 1/18) | |
| | | NSW (near Sydney) | *Theileria* sp. | -Molecular (nPCR; + Sanger sequencing [PCR products]) – Piroplasms, Hepatozoon<br>[blood samples]<br>- gel electrophoresis (PCR products)<br>- Morphological (light microscopy)<br>[blood] | 72.2% (n = 13/18) | |
| | | NSW (near Sydney) | *Babesia* sp.<br>(*Ba. lohae*-like) | | 72.2% (n = 13/18) | |
| | *I. australiensis* | Upper Warren, WA | *Trypanosoma* spp. (DNA) | - Molecular (HRM-qPCR; then Sanger sequencing)<br>- gel electrophoresis (PCR products)<br>[tick samples] | Not reported | [69] |
| | *I. myrmecobii* | | | | Not reported | |
| | *I. tasmani* | | | | Not reported | |

| | | | | | | |
|---|---|---|---|---|---|---|
| | *I. holocyclus* (adult [f]) | Beerwah, QLD | *Babesia* sp. | - Molecular (nPCR; then Sanger sequencing)<br>- gel electrophoresis (PCR products)<br>[tick samples] | 14.3% (n = 1/7) | [64] |
| | *I. tasmani* (adults [f]) | | | | 28.6% (n = 2/7) | |
| | *I. tasmani* (adult [f]) | Royal Botanic Garden, NSW | *Babesia* sp. | | 100% (n = 1/1) | |
| | Not reported | Darwin, NT<br>Kakadu National Park, NT<br>Bathurst Island, NT<br>Groote Eylandt, NT<br>Cobourg Peninsula, NT | *Trypanosoma noyesi* | Molecular (nPCR, then Sanger sequencing)<br>[blood samples] | 23.7% (n = 9/38) | [166] |
| Western Quoll [Chuditch] | Not reported | Not reported | *Trypanosoma gilletti* | - Molecular (NGS [Illumina]; + semi-nPCR, + cPCR [ampl. of nPCR products]) -- *Trypanosomes*<br>[tissue (skin/spleen) samples, blood samples] | Not reported | [125] |
| | | | *Trypanosoma cyclops*-like (sequence) | | 4.5% (n = 1/22) | |
| Bush Rat | Not reported | Kioloa, NSW | *Hepatozoon* sp. R1 | - Molecular (NGS [Illumina]; + cPCR, qPCR [confirm detection], + Sanger sequencing (PCR products) )<br>- gel electrophoresis (PCR products)<br>[wildlife blood sample] | 27.3% (n = 3/11) | [31] |
| | | | *Hepatozoon* sp. R2 | | 9% (n = 1/11) | |
| | | | *Trypanosoma* cf. *lewisi* | | 45.5% (n = 5/11) | |
| | Not reported | North Head, NSW<br>Manly Dam, NSW | *Trypanosoma lewisi*-like (sp. BR042) | - Molecular (NGS [Illumina]; + semi-nPCR, + cPCR [ampl. of nPCR products]) -- *Trypanosomes*<br>[tissue (skin/spleen) samples, blood samples] | Not reported | [125] |
| | | Not reported | *Hepatozoon* sp. | -Molecular (nPCR; + Sanger sequencing [PCR products]) – Piroplasms, Hepatozoon<br>[blood samples]<br>- gel electrophoresis (PCR products)<br>- Morphological (light microscopy)<br>[blood] | 100% (n = 2/2) | |
| Black Rat | Not reported | Sydney NSW | *Hepatozoon* sp. R1 | - Molecular (NGS [Illumina]; + cPCR, qPCR [confirm detection], + Sanger sequencing (PCR products))<br>- gel electrophoresis (PCR products)<br>[wildlife blood sample] | 3% (n = 1/33) | [31] |
| | | | *Hepatozoon* sp. R2 | | 3% (n = 1/33) | |
| | Not reported | Not reported | *Trypanosoma gilletti* | - Molecular (NGS [Illumina]; + semi-nPCR, + cPCR [ampl. of nPCR products]) -- *Trypanosomes*<br>[tissue (skin/spleen) samples, blood samples] | Not reported | [125] |
| | | | *Trypanosoma cyclops*-like (sequence) | | 10% (n = 7/70)<br>[blood] | |

| | | | | | | |
|---|---|---|---|---|---|---|
| | | | | | | |
| | | | *Trypanosoma cyclops* | - Molecular (NGS [Illumina]; + semi-nPCR, + cPCR [ampl. of nPCR products]) -- *Trypanosomes* [blood samples]<br>- Morphological (light microscopy) [blood] | 1.4% (n = 1/70) | |
| | | North Head, NSW | *Trypanosoma lewisi*-like | - Molecular (NGS [Illumina]; + semi-nPCR, + cPCR [ampl. of nPCR products]) -- *Trypanosomes* [blood samples] | 4.3% (n = 3/70) | |
| | | Manly Dam, NSW | | | | |
| | | NSW (unspecified) | *Theileria c.f. peramelis* | -Molecular (nPCR; + Sanger sequencing [PCR products]) – Piroplasms, Hepatozoon [blood samples]<br>- gel electrophoresis (PCR products)<br>- Morphological (light microscopy) [blood] | 12.9% (n = 9/70) | |
| | | Not reported | *Hepatozoon* sp. | | 1.4% (n = 1/70) | |
| Quenda | *Am. triguttatum* (nymph) | WA (unspecified) | *Trypanosoma vegrandis* | - Molecular (NGS [Illumina]; + cPCR, qPCR [confirm detection], + Sanger sequencing (PCR products) )<br>- gel electrophoresis (PCR products) [tick samples] | 25% (n = 1/4) | [125] |
| | *I. fecialis* | Upper Warren, WA | *Trypanosoma* spp. (DNA) | - Molecular (HRM-qPCR; then Sanger sequencing)<br>- gel electrophoresis (PCR products) [tick samples] | 100% (n = 1/1) | [69] |
| Long-nosed Potoroo | Not reported | NSW (Nadgee, Timbillica, East Boyd) | *Trypanosoma gilletti* | - Molecular (cPCR; then Sanger sequencing)<br>- gel electrophoresis (PCR products) [blood samples] | 22.2% (n = 8/36) | [210] |
| | | | *Trypanosoma vegrandis* | | 2.8% (n = 1/36) | |
| | | | *Trypanosoma* sp. (cyclops genotype) | | 13.9% (n = 5/36) | |
| | | | | | 2.8% (n = 1/36) | |
| | | | | | 2.8% (n = 1/36) | |
| Brush-tailed Bettong [‘Woylie’] | *Am. triguttatum* (nymph) | Upper Warren, WA | *Trypanosoma* spp. (DNA) | - Molecular (HRM-qPCR; then Sanger sequencing)<br>- gel electrophoresis (PCR products) [tick samples] | Not reported | [69] |
| | *I. woyliei* (adults [f], nymph) | | | | Not reported | |
| | *I. australiensis* (adults [m+f], nymph) | | | | Not reported | |
| | *I. myrmecobii* (adults [f], nymph) | | | | Not reported | |
| | *Ixodes* sp.(larva) | | | | Not reported | |

| | | | | | | |
|---|---|---|---|---|---|---|
| | *Amblyomma* sp.(larva) | | | | Not reported | |
| | Not reported (N/A) | Dwellingup, WA | *Babesia* sp. (DNA) | - Molecular (nPCR; + Sanger sequencing)<br>- gel electrophoresis (PCR products) [blood sample]<br>- Morphological (light microscopy) [blood smear] | Smears -- 50% (n = 6/12)<br><br>Blood – 46.7% (n = 7/15) | [167] |
| | Not reported | Avon Valley, WA | *Theileria penicillata* | - Molecular (cPCR)<br>- Morphological (light microscopy) [blood sample] | 100% (n = 1/1) | [180] |
| | Not reported | Dryandra, WA | *Trypanosoma copemani* | Molecular (nPCR, then Sanger sequencing) [blood samples] | 23.7% (n = 95/401) | [168] |
| | | Walcott, WA | | | 24% (n = 113/470) | |
| | | Warrup East, WA | | | 20.9% (n = 71/339) | |
| | | Dryandra, WA | *Trypanosoma vegrandis* | | 24.7% (n = 99/401) | |
| | | Walcott, WA | | | 45.7% (n = 215/470) | |
| | | Warrup East, WA | | | 19.2% (n = 65/339) | |
| | | Dryandra, WA | *Trypanosoma noyesi* | | 5% (n = 20/401) | |
| | | Walcott, WA | | | 7.7% (n = 36/470) | |
| | | Warrup East, WA | | | 7.8% (n = 31/399) | |
| | | Dryandra, WA | *Trypanosoma* sp. ANU2 | | 0.25% (n = 1/401) | |
| | | Walcott, WA | | | 3.4% (n = 16/470) | |
| | | Warrup East, WA | | | 3% (n = 10/339) | |
| | | Dryandra, WA | *Theileria penicillata* | | 86.6% (n = 97/112) | |
| | | Walcott, WA | | | 73.4% (n = 47/64) | |
| | | Warrup East, WA | | | 57.5% (n = 50/87) | |
| | | Dryandra, WA | *Theileria apogeana* | | 3.4% (n = 1/29) | |
| | | Walcott, WA | | | 18.5% (n = 12/65) | |
| | | Warrup East, WA | | | 34.5% (n = 30/87) | |
| | | Dryandra, WA | *Babesia* sp. | | 9.8% (n = 11/112) | |
| | | Walcott, WA | | | 1.5% (n =1/65) | |
| | Not reported | Karakamia, WA | *Theileria penicillata* | - Molecular (nPCR; then Sanger sequencing) [blood samples]<br>- Morphological (light microscopy)<br>- Gel electrophoresis (PCR products) | Karakamia:<br>- LM: 100% (n = 4/4)<br>- PCR: 97.7% (n = 43/44) | [181] |
| | | Kingston [Winnejup, Corbal], WA | | | Corbal:<br>- PCR: 100% (n = 20/20) | |
| | | | | | Winnejup:<br>- PCR: 73.7% (n = 14/19) | |
| | | Perup [Keninup, Boyicup], WA | | | Keninup:<br>- LM: 100% (n = 24/24)<br>- PCR: 100% (n = 35/35) | |
| | | | | | Boyicup:<br>- PCR: 100% (n = 5/5) | |

| | | | | | | |
|---|---|---|---|---|---|---|
| Tammar Wallaby | *Amblyomma* sp. (larvae) | Upper Warren, WA | *Trypanosoma* spp. (DNA) | - Molecular (HRM-qPCR; then Sanger sequencing)<br>- gel electrophoresis (PCR products)<br>[tick samples] | Not reported | [69] |
| Burrowing Bettong [‘Boodie’] | Not reported (N/A) | Kanyana, WA | *Theileria* sp. (rDNA) | - Molecular (nPCR; + Sanger sequencing)<br>- gel electrophoresis (PCR products)<br>[blood sample]<br>- Morphological (light microscopy) [blood smear] | Smear – 33.3% (n = 1/3)<br>Blood -- 33.3% (n = 1/3) | [167] |
| Tasmanian Devil | Not reported | Black River, TAS | *Trypanosoma* spp. | - Molecular (nPCR; then semi-nested PCR) -- *Trypanosoma + Leishmania*<br>- Molecular (nPCR) – *Babesia + Theileria*<br>- Molecular (Sanger sequencing) -- ALL<br>- gel electrophoresis<br>[blood sample] | 35.5% (n = 11/31) | [169] |
| | | | *Trypanosoma copemani* | | 6.5% (n = 2/31) | |
| | | | *Trypanosome cyclop*-like | | 29% (n = 9/31) | |
| | | | *Babesia* sp. | | 3.2% (n = 1/31) | |
| | | Takone, TAS | *Trypanosoma* spp. | | 30.4% (n = 7/23) | |
| | | | *Trypanosoma copemani* | | 21.7% (n = 5/23) | |
| | | | *Trypanosoma cyclop*-like | | 8.7% (n = 2/23) | |
| | | West Pencil Pine, TAS | *Trypanosoma* spp. | | 78.6% (n = 11/14) | |
| | | | *Trypanosoma cyclop*-like | | 78.6% (n = 11/14) | |
| | | Freycinet, TAS | *Trypanosoma* spp. | | 11.1% (n = 3/27) | |
| | | | *Trypanosoma copemani* | | 11.1% (n = 3/27) | |
| | *I. tasmani* (adults [m+f], nymphs) | Wall Woolnorth, TAS | *Hepatozoon* sp. | - Molecular (cPCR, then Sanger sequencing)<br>[tick samples]<br>- Gel electrophoresis (PCR products) | 40% (n = 4/10) | [156] |
| | | Surrey Hills, TAS | | | 33.3% (n = 5/15) | |
| | | Bronte Park, TAS | | | 42.6% (n = 6/14) | |
| Sleepy Lizard [Shingleback skink] | *Am. limbatum* (adults [m+f], nymphs) | Mt. Mary, SA | *Hemolivia mariae* | - Morphological (light microscopy)<br>[tick samples (gut content), blood samples] | Nymphs --14.4% (n= 14/104)<br>Adults -- 40.6% (n = 129/318)<br>Lizards -- 12.7% (n = 23/181) | [225] |
| Yellow-spotted monitor | *Am. fimbriatum* (adults [m+f]) | NT (Djukbinj National Park, Fogg Dam Nature Reserve, Humpty Doo) | - *Hepatozoon* sp. DNA<br>- *Hepatozoon* sp. | - Molecular (cPCR, then Sanger sequencing [BigDye Term.])<br>[tick samples]<br>- gel electrophoresis (PCR products)<br><br>- Morphology (light microscopy)<br>[blood] | 58.4% (n = 87/149)<br>[cPCR]<br>66.7% (n = 4/6) [seq.] | [46] |
| | *Am. moreliae* (adult [f]) | | *Hepatozoon* sp. DNA | | 100% (n = 1/1) | |
| | | | | | 35.5% (11/31)<br>[blood] | |
| Water Python | *Am. fimbriatum* (adults [m+f]) | NT (Djukbinj National Park, Fogg Dam Nature Reserve, Humpty Doo) | - *Hepatozoon* sp. DNA<br>- *Hepatozoon* sp. | - Molecular (cPCR, then Sanger sequencing [BigDye Term.])<br>[tick samples]<br>- gel electrophoresis (PCR products) | 7% (n = 13/187)<br>(*Am.* moreliae -- n = 2/2)<br>-- [cPCR] | [46] |
| | *Am. limbatum* (adults [m+f]) | | *Hepatozoon* sp. DNA | | | |

| | | | | | | |
|---|---|---|---|---|---|---|
| | *Am. moreliae* (adults [m+f]) | | - *Hepatozoon* sp. DNA<br>- *Hepatozoon* sp. | - Morphology (light microscopy)<br>[blood sample ] | *Am. moreliae* – 16.7% (n = 1/6)<br><br>*Am. fimbriatum* 16.7% (n = 1/6) | |
| | *Amblyomma* sp. (nymph) | | *Hepatozoon* sp. DNA | | 50% (n = 2/4)<br>[blood] | |
| Frilled-neck Lizard | *Am. limbatum* (adults [m+f])<br>*Amblyomma* sp. (nymph) | NT (Djukbinj National Park, Fogg Dam Nature Reserve, Humpty Doo) | *Hepatozoon* sp. DNA | - Molecular (cPCR)<br>- Morphology (light microscopy)<br>[blood] | 2.1% (n = 4/187) | [46] |
| Green-tree Snake | *Am. fimbriatum*<br>*Am. limbatum* | NT (Djukbinj National Park, Fogg Dam Nature Reserve, Humpty Doo) | *Hepatozoon* sp. DNA | - Molecular (cPCR,<br>- Morphology (light microscopy)<br>[blood] | 1.6% (n = 3/187) | [46] |
| | *Amblyomma* sp. (nymph) | | | | 75% (n = 3/4)<br>[blood] | |
| Red-necked Wallaby | *Ixodes* sp. (adult [f], nymph, larvae) | Mid-North Coast, NSW (unspecified) | *Piroplasm rRNA* | - Molecular (qPCR [x2]; touchdown PCR; and then Sanger sequencing)<br>- gel electrophoresis (PCR products) | Larvae: (n = 11/23) | [73] |
| | *Haemaphysalis* sp. (nymphs, larvae) | | | | Larvae: (n = 61/96)<br>Nymph: (n = 3/96) | |
| | *H. petrogalis* (larvae) | | | | (n = 24/96) | |
| Little Penguin | Not reported | Cabbage Tree Island, NSW | *Babesia* sp. | - Morphological (light microscopy)<br>[blood smears]<br>- Molecular (nPCR [x2]; then Sanger sequencing)<br>- gel electrophoresis (PCR products) | Smear – 20% (n = 2/10) | [170] |
| | | Summerland Estate [Phillip Island], VIC | | | Smear – (n = 1/12) | |
| | | Darlington Foreshore [Maria Island], TAS | | | Smear – (n = 2/7) | |
| | | The Neck [Bruny Island], TAS | | | Smear – (n = 2/27) | |
| Eastern Bettong | Tick(s) unspecified:<br>- *I. tasmani*<br>- *I. cornuatus*<br>- *I. trichosuri* | TAS (Long Hill, Pipers River, Mt. Morrison, Bangor, The Lea, Grove, Castle Forbes Bay, Geeveston) | *Piroplasm* sp. | - Morphological (light microscopy)<br>[blood smear] | 6.7% (n = 4/60) | [212] |
| | Not reported | Tidbinbilla Reserve, ACT | *Theileria* sp. (*T. lupei*) | - Molecular (cPCR, + nPCR; then Sanger sequencing)<br>- gel electrophoresis (PCR products)<br>- Morphological (light microscopy) | 100% (n = 2/2)<br>[blood sample] | [174] |
| Eastern Quoll | Tick(s) unspecified:<br>- *I. tasmani* | TAS (Bronte, Uxbridge, Judbury, | *Hepatozoon* sp.<br>*Hepatozoon banethi* | - Molecular (hemi-nested PCR; then Sanger sequencing)<br>- gel electrophoresis (PCR products) | 33.3% (n = 5/15) | [182] |

| | | | | | | |
|---|---|---|---|---|---|---|
| | - *I. fecialis*<br>- *I. cornuatus* (misidentified as *I. holocyclus*) | Grove, Geeveston, Cygnet) | *Theileria* sp. | [blood samples] | 6.7% (n = 1/15) | |
| | | | *Theileria paparinii* | | 20% (n = 3/15) | |
| | Not reported | Tidbinbilla Reserve, ACT | *Theileria* sp. (*T. lupei*) | - Molecular (cPCR, + nPCR; then Sanger sequencing)<br>- gel electrophoresis (PCR products)<br>- Morphological (light microscopy) | 100% (n = 2/2) | [174] |
| Koala | *I. holocyclus* | QLD (unspecified) | -*Trypanosoma irwini*<br>-*Trypanosoma gilletti*<br>-*Trypanosoma copemani*<br>-*Trypanosoma vegrandis*<br>-*Trypanosoma* sp.<br>*Trypanosoma noyesi* | - Molecular (hemi-nested PCR; then NGS [Illumina -- PCR amplicons])<br>- gel electrophoresis (PCR products)<br>[blood + tick samples] | 34.3% (n = 12/35) | [67] |
| | *I. tasmani* | | | | 19.6% (n = 11/56) | |
| | Blood | | | | 35.1% (n = 59/168) | |
| | *I. tasmani* | Portland, VIC | *Apicomplexa* sp. | - Molecular (microfluidic real-time PCR, cPCR; then Sanger sequencing) | 75% (n = 3/4) | [32] |
| | | | *Theileria* sp. | | 25% (n = 1/4) | |
| Bare-Nosed Wombat | *Ap.* (*Both.*) *auruginans* | Wilson's Promontory | *Apicomplexa* sp. | - Molecular (microfluidic real-time PCR, cPCR; then Sanger sequencing) | 100% (n = 2/2) | [32] |
| Mountain Brushtail Possum | *I. trichosuri* | Boho South, VIC | *Apicomplexa* sp. | - Molecular (microfluidic real-time PCR, cPCR; then Sanger sequencing) | 70% (n = 7/10) | [32] |
| | *I. tasmani* | | | | 100% (n = 2/2) | |
| | *I. trichosuri* | | *Theileria* sp. | | 20% (n = 2/10) | |
| Agile Antechinus | *I. antechini* | Boho South, VIC | *Apicomplexa* sp. | - Molecular (microfluidic real-time PCR, cPCR; then Sanger sequencing) | 100% (n = 4/4) | [32] |
| | *I. tasmani* | | | | 100% (n = 1/1) | |
| | *I. antechini* | | *Theileria* sp. | | 100% (n = 4/4) | |
| | *I. tasmani* | | | | 100% (n = 1/1) | |
| Southern Brown Bandicoot | *I. tasmani* | Koo Wee Rup, VIC | *Apicomplexa* sp. | - Molecular (microfluidic real-time PCR, cPCR; then Sanger sequencing) | 100% (n = 8/8) | [32] |
| | *I. trichosuri* | VIC | | | 87.5% (n = 7/8) | |
| | *I. tasmani* | Koo Wee Rup, VIC | *Theileria* sp. | | 25% (n = 2/8) | |
| Quenda | Not reported | Perth, WA | *Hepatozoon* sp. | - Molecular (cPCR; then Sanger sequencing)<br>- Morphological (light microscopy)<br>[blood samples] | 58.1% (n = 18/31) [molecular] | [232] |
| | | | | | 48% (n = 25/52) [morphological] | |
| Gilbert's Potoroo | *I. australiensis* | Two Peoples Bay, WA | *Trypanosoma copemani* | - Morphological (light microscopy)<br>[tick: haemolymph, mid-gut, faeces, bloodmeal]<br>- Molecular (cPCR; then Sanger sequencing) | Haemolymph, Midgut, Bloodmeal (n = 1/2) | [185] |
| | *I. australiensis* (adult) | Two Peoples Bay, WA | *Theileria gilberti* | - Molecular (nPCR; then Sanger sequencing)<br>[tick, blood, tissue samples]<br>- Morphological (light microscopy) [blood sample]<br>- gel electrophoresis (PCR products) | Smear: 31.3% (n = 5/16)<br>PCR: 100% (n = 16/16)<br>Found in both tick spp. | [183] |
| | *I. fecialis* (adult) | | | | | |

| | | | | | | |
|---|---|---|---|---|---|---|
| | Not reported | Two Peoples Bay, WA | *Theileria worthingtonorum* | - Molecular (cPCR, + nPCR; then Sanger sequencing)<br>- gel electrophoresis (PCR products)<br>[blood sample] | Not reported | [174] |
| Long-nosed Potoroo | Not reported | VIC (unspecified) | Piroplasm sp. | Molecular (nPCR)<br>[tissue (liver) sample] | 100% (n = 5/5) | [183] |
| Quokka | *I. australiensis* | Bald Island, WA | *Trypanosoma copemani* | - Morphological (light microscopy)<br>[tick: haemolymph, mid-gut, faeces, bloodmeal]<br>- Molecular (cPCR; then Sanger sequencing) | Faeces, Bloodmeal – 12.5% (n = 1/8)<br>Midgut – 20% (n = 2/10) | [185] |
| | Not reported | Two Peoples Bay, WA | *Theileria worthingtonorum* | - Molecular (cPCR, + nPCR; then Sanger sequencing)<br>- gel electrophoresis (PCR products)<br>- Morphological (light microscopy) | 100% (n = 18/18) | [174] |
| | | | *Theileria brachyuri* | | 22.2% (n = 4/18) | |
| | | Bald Island, WA | *Theileria* sp. | | 100% (n = 6/6) | |
| | Not reported | Bald Island, WA | *Theileria brachyuri* | - Molecular (cPCR)<br>- Morphological (light microscopy) | 100% (n = 2/2) | [180] |
| Little Blue Penguin | *I. kohlsi* | Phillip Island, VIC | *Apicomplexa* sp. | - Molecular (microfluidic real-time PCR, cPCR; then Sanger sequencing)<br>- Molecular (qPCR) – *Rickettsia* | 66.7% (n = 4/6) | [32] |
| Agile Wallaby | Not reported (none) | Townsville, QLD | *Babesia macropus* | - Morphological (histopath.; + cytology [impression smear]; + blood – light microscopy; electron microscopy)<br>[tissue samples: kidney, liver, brain, spleen, bone marrow]<br>- Molecular (cPCR, then Sanger sequencing)<br>[tissue samples: kidney, liver, brain, spleen, bone marrow] | Histo: 100% (n = 3/3)<br>Blood: 100% (n = 1/1)<br>PCR: 66.7% (n = 2/3) | [165] |
| Swamp Wallaby | Not reported | Tidbinbilla Reserve, ACT | *Theileria* sp. (*T. lupei*) | - Molecular (cPCR, + nPCR; then Sanger sequencing)<br>- gel electrophoresis (PCR products)<br>- Morphological (light microscopy) | 100% (n = 1/1) | [174] |
| 'Bandicoot' (unidentified) | *I. tasmani* (adult [f]) | Port Sorell, TAS | *Theileria* sp. | - Molecular (nPCR; then Sanger sequencing)<br>- gel electrophoresis (PCR products)<br>[tick samples] | 100% (n = 1/1) | [64] |
| | | Beerwah, QLD | *Theileria* sp. | | 100% (n = 1/1) | |
| Red fox | *H. longicornis* (nymph) | Grosevale, NSW | *Theileria orientalis* | - Molecular (nPCR; then Sanger sequencing)<br>- gel electrophoresis (PCR products)<br>[tick samples] | 33.3% (n = 1/3) | [64] |
| New Holland Honeyeater | *I. hirsti* | Newland Head Conservation Park, SA | *Haemoproteus* sp. | - Morphological (light microscopy)<br>[blood smear] | 5.4% (n = 2/37) | [78] |
| | Not reported | Sandy Creek, SA | | | 15.1% (n = 8/53) | |

| | | | | | | |
|---|---|---|---|---|---|---|
| Northern Quoll | Not reported | Kimberley [Mitchell Plateau], WA | *Babesia thylacis* | - Morphological (light microscopy) [blood smear] | 100% (n = 1/1) | [171] |
| Questing Ticks (collected in same area as wildlife: - Black Rat - Bush Rat - Brush-tailed Possum - Long-nosed Bandicoot) | *H. bancrofti* | Kioloa, NSW | *Babesia mackerrasorum* | - Molecular (NGS [Illumina]; + cPCR, qPCR [confirm detection], + Sanger sequencing (PCR products) ) - gel electrophoresis (PCR products) [tick samples] | 67.7% (n = 21/31) | [31] |
| | | Sydney, NSW | | | 100% (n = 1/1) | |
| | | Kioloa, NSW | *Hepatozoon ewingi* | | 22.6% (n = 7/31) | |
| | | | *Hepatozoon* sp. HB | | 32.3% (n =10/31) | |
| | | | *Trypanosoma vegrandis* | | 12.9% (n = 4/31) | |
| | | | *Trypanosoma* sp. nov. HB | | 32.2% (n = 10/31) | |
| | *I. trichosuri* | Sydney, NSW | *Babesia lohae* | | 100% (n = 1/1) | |
| | *I. holocyclus* | Kioloa, NSW | *Theileria* sp. cf. AU-1048 | | 22.2% (n = 22/99) | |
| | | | *Theileria* sp. IH | | 3% (n = 3/99) | |
| | | Sydney, NSW | *Theileria* cf. peramelis | | 6.5% (n = 3/46) | |
| | | Kioloa, NSW | *Trypanosoma gilletti* | | 19.1% (n = 19/99) | |
| | | Sydney, NSW | | | 6.5% (n = 3/46) | |
| Questing Ticks (collected in same area as wildlife: - Woylie - Tammar wallaby - Brush-tailed possum) | *Am. triguttatum* (adults [m+f], nymph) | Upper Warren, WA | *Trypanosoma* spp. (DNA) | - Molecular (HRM-qPCR; then Sanger sequencing) - gel electrophoresis (PCR products) [tick samples] | Not reported | [69] |
| | *I. australiensis* (adults [m+f], nymph) | | | | | |
| | *I. myrmecobii* (adults [f]) | | | | | |
| | *Ixodes* sp. (larvae) | | | | | |
| Not reported (i.e. collected off a wildlife host) | *I. tasmani* (adults [f]) | Not reported | *Trypanosoma gilletti* | Molecular (NGS [Illumina]; + semi-nPCR, + cPCR [ampl. of nPCR products]) -- *Trypanosomes* [tick samples] | Not reported | [125] |
| | *I. trichosuri* (adults [f], nymph) | | | | | |
| | *I. holocyclus* (adults [f], nymphs, larvae) | | | | | |
| | *H. petrogalis* (adult [f]) | Mid-North Coast NSW (unspecified) | *Piroplasm* rRNA | - Molecular (qPCR [x2]; touchdown PCR; and then Sanger sequencing) - gel electrophoresis (PCR products) | (n = 3/20) | [73] |
| Viruses | | | | | | |
| Host(s) unspecified: - White-Capped Noddies - Common Noddies - Wedge-tailed Shearwaters | Not reported | - Lihou Reef - Flinders Reef - Marion Reef - Creal Reef - Fredrick Reef - Gannet Cay - Cato Island - Heron Island | Gadget's Gully virus | - Serology (neutralization tests; presence of neutralizing antibodies) | 4% (n = 12/300) | [193] |
| | | | Saumarez Reef virus | | 5% (n = 19/380) | |
| | | | Upolu virus | | 10.4% (n = 41/394) | |
| | | | Johnston Atoll virus | | 3.5% (n = 14/367) | |
| | | | Taggert virus | | 0.3% (n = 1/300) | |
| | | | Lake Clarendon virus | | 2.7% (n = 8/300) | |
| | | | CSIRO 1499 virus? | | 3.2% (n = 13/401) | |
| | | | CSIRO 976 virus? | | 1.2% (n = 3/261) | |

| | | | | | | |
|---|---|---|---|---|---|---|
| - Lesser Frigatebirds<br>- Red-footed Booby<br>- Masked Booby<br>- Brown Booby | | - Masthead Island<br>- Musgrave Island<br>[unspecified] | CSIRO 264 virus | | 1.2% (n = 3/261) | |
| Shy Albatross | *I. eudyptidis* | Albatross Island, TAS | Hunter Island Group virus | - Morphological (electron microscopy, CPE [Vero cell -- tick sample])<br>- Molecular (cPCR; then NGS [FLX]) | CPE -- 40% (n= 2/5) | [111] |
| | *I. eudyptidis* | Albatross Island, TAS | Albatross Island virus | - Molecular (sequencing [high-throughput]) | Not reported | [112] |
| King Penguin | *I. uriae* (adult [f]) | Macquarie Island (Sandy Bay), TAS | Gadget's Gully virus | - Morphological (electron microscopy, CPE [BHK cells])<br>- Molecular (Sanger sequencing) | 2.2% (n = 2/90) | [194] |
| | *I. uriae* (adult [m+f]) | | Sandy Bay virus | | 5.6% (n = 5/90) | |
| Rockhopper Penguin | *I. uriae* (adults [m]) | Macquarie Island (Catch-me- cave), TAS | Catch-me- cave virus | - Morphological (electron microscopy, CPE [BHK + Vero cells]) | 5.6% (n = 2/36) | [194] |
| Royal Penguins | *I. uriae* | Macquarie Island (Upper Finch Ck), TAS | Finch Creek virus | - Morphological (electron microscopy, CPE [BHK cells]) | 2.3% (n = 1/43) | [194] |
| Long-nosed bandicoot | *I. holocyclus* | North Head Sydney, NSW | Collins Beach virus 1 | - Molecular (NGS [Illumina]; then RT-PCR; then WGS [Illumina])<br>[tick, + tissue samples] | Not reported | [196] |
| | *I. trichosuri* | Sydney, NSW | Fairfax Lookout virus | - Molecular (NGS [Illumina])<br>[tick samples] | Not reported | [86] |
| | *I. holocyclus* | Sydney, NSW | Collins Beach virus | | | |
| | | | Blue Fish Point virus | | | |
| | | | Shelly Headland virus | | | |
| | | | Shelly Beach virus | | | |
| | | | Old Quarry Swamp virus | | | |
| | | | Jump Rock virus | | | |
| | | | Ingleside virus | | | |
| | | | Wangarabell virus | | | |
| | | | Yambulla virus | | | |
| | | | Nadgee virus | | | |
| | | | Genoa virus | | | |
| Bush Rat | *I. trichosuri* | Sydney, NSW | Fairfax Lookout virus | Molecular (NGS [Illumina])<br>[tick samples] | Not reported | [86] |
| Blue-tongue Lizard | *Am. moreliae* | Sydney, NSW | Store Beach virus | Molecular (NGS [Illumina])<br>[tick samples] | Not reported | [86] |
| | | | Quarantine Head virus | | | |
| | | | Fairlight virus | | | |
| | | | Manly virus | | | |
| | | | Cannae Point virus | | | |

| | | | | | | |
|---|---|---|---|---|---|---|
| Southern Brown Bandicoot | *I. holocyclus* | Timbillica, NSW | Shelly Beach virus | Molecular (NGS [Illumina]) [tick samples] | Not reported | [86] |
| | | | Old Quarry Swamp virus | | | |
| | | | Timbillica virus | | | |
| Cattle Egret | *Ar. robertsi* | Lake Clarendon, QLD | Lake Clarendon virus (CSIRO 704) | - Mouse Inoculation [intracerebral]<br>- Morphological (CPE – via cell culture [BHK21]; electron microscopy) [tick samples]<br>- Serology (microneutralization test) [sera] | Sera – 78.4% (n = 29/37) | [62] |
| Not reported | *Ar. robertsi* | Adelaide River, NT | Kao Shaun virus | Virus isolation (no details) | Not reported | [61] |
| Not reported | *I. holocyclus* | Lismore-Nimbin, NSW (RT-PCR samples) | *Ixodes holocyclus iflavirus* (IhIV) | - Molecular (NGS [Illumina; 454]; then RT-PCR) [Tick samples] | 44% (n = 11/25) | [195] |
| | | Suffolk Park, NSW | | | | |
| | | Pinjarra Hills, QLD | | | | |
| | | Brisbane, QLD | | | | |
| Questing Ticks (collected in same area as wildlife:<br>- Black Rat<br>- Bush Rat<br>- Brush-tailed Possum<br>- Long-nosed Bandicoot) | *I. holocyclus* | Sydney, NSW | *Ixodes holocyclus iflavirus* (IhIV) | - Molecular (NGS [Illumina]; + cPCR, qPCR [confirm detection], + Sanger sequencing (PCR products))<br>- gel electrophoresis (PCR products) [tick samples] | 4.35% (n = 2/46) | [31] |
| | | Kioloa, NSW | North Shore virus | | 44.4% (n = 44/99) | |
| | | Sydney, NSW | | | 56.5% (n = 26/46) | |
| | | Kioloa, NSW | Shelly Headland virus | | 5.1% (n = 5/99) | |
| | | Sydney, NSW | | | 19.6% (n = 9/46) | |
| | | Sydney, NSW | Shelly Beach virus | | 2.2% (n = 1/46) | |
| | | Kioloa, NSW | Timbillica virus | | 38.4% (n = 38/99) | |
| | | Sydney, NSW | | | 67.4% (n = 31/46) | |
| | | Kioloa, NSW | Genoa virus | | 14.1% (n = 14/99) | |
| | | Sydney, NSW | Ingleside virus | | 67.4% (n = 31/46) | |
| | | Kioloa, NSW | Newport Tick virus | | 41.1% (n = 41/99) | |
| | | Sydney, NSW | | | 54.3 % (n = 25/46) | |
| | *H. bancrofti* | Kioloa, NSW | Kioloa Tick virus | | 74.2% (n = 23/31) | |
| | | | Scerri virus | | 25.8% (n = 8/31) | |
| | | | Butlers Creek Tick virus | | 22.6% (n = 7/31) | |
| | | | O'Hara Headland virus | | 3.2% (n = 1/31) | |

**Abbreviations.** Life stages: m, male; f, female; nymph and larvae as stated.
States and territories: ACT, Australian Capital Territory; NSW, New South Wales; NT, Northern Territory; QLD, Queensland; SA, South Australia; TAS, Tasmania; VIC, Victoria; WA, Western Australia.
Detection methods: PCR, polymerase chain reaction; cPCR, conventional PCR; nPCR, nested PCR; qPCR, quantitative (real-time) PCR; RT-PCR, reverse-transcription PCR; HRM-qPCR, high-resolution melt quantitative PCR; NGS, next-generation sequencing; WGS, whole-genome sequencing; ELISA, enzyme-linked immunosorbent assay; CPE, cytopathic effect.

Genetic markers: 16S rRNA, 16S ribosomal RNA; 18S rDNA, 18S ribosomal DNA; rRNA, ribosomal RNA; com1, Coxiella outer-membrane protein gene.

Taxonomic and other terms: sp., species (singular); spp., species (plural); nov., novel/new (e.g. sp. nov., new species); cf. (c.f.), confer (tentative identification); Ca., Candidatus (provisionally named taxon); OTU, operational taxonomic unit; str., strain; AUSBR, Australian Borrelia lineage; n, number.
